# A first view on the unsuspected intragenus diversity of N‐glycans in *Chlorella* microalgae

**DOI:** 10.1111/tpj.14718

**Published:** 2020-03-17

**Authors:** Réka Mócsai, Rudolf Figl, Leander Sützl, Silvia Fluch, Friedrich Altmann

**Affiliations:** ^1^ Department of Chemistry Vienna (BOKU) Vienna Austria; ^2^ Department of Food Technology University of Natural Resources and Life Sciences Vienna (BOKU) Vienna Austria; ^3^ Ecoduna AG Bruck an der Leitha Austria

**Keywords:** microalgae, N‐glycans, *Chlorella vulgaris*, *Chlorella sorokiniana*, *Chlorella pyrenoidosa*, arabinose, methylated sugar

## Abstract

*Chlorella* microalgae are increasingly used for various purposes such as fatty acid production, wastewater processing, or as health‐promoting food supplements. A mass spectrometry‐based survey of N‐glycan structures of strain collection specimens and 80 commercial *Chlorella* products revealed a hitherto unseen intragenus diversity of N‐glycan structures. Differing numbers of methyl groups, pentoses, deoxyhexoses, and *N*‐acetylglucosamine culminated in *c.* 100 different glycan masses. Thirteen clearly discernible glycan‐type groups were identified. Unexpected features included the occurrence of arabinose, of different and rare types of monosaccharide methylation (e.g. 4‐*O*‐methyl‐*N*‐acetylglucosamine), and substitution of the second *N*‐acetylglucosamine. Analysis of barcode ITS1–5.8S–ITS2 rDNA sequences established a phylogenetic tree that essentially went hand in hand with the grouping obtained by glycan patterns. This brief prelude to microalgal N‐glycans revealed a fabulous wealth of undescribed structural features that finely differentiated *Chlorella*‐like microalgae, which are notoriously poor in morphological attributes. In light of the almost identical N‐glycan structural features that exist within vertebrates or land plants, the herein discovered diversity is astonishing and argues for a selection pressure only explicable by a fundamental functional role of these glycans.

## Significance Statement

The protein N‐glycosylation machinery of green algae has hitherto been considered as being essentially similar to that of land plants. The current work reveals a plethora of yet unknown N‐glycan structures only within the *Chlorella* clade – an economically relevant branch – with considerable repercussions on taxonomy and functional significance.

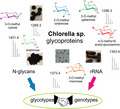

## Introduction

Green microalgae from the *Chlorella* clade are grown on a large scale for various purposes including protein‐rich animal feed (Guccione *et al.*, 2014; Bleakley and Hayes, 2017; Grossmann *et al.*, 2019), as there are indications that algae may be grown on various types of sewage or sludge (Markou *et al.*, 2018; Eladel *et al.*, 2019). Some studies have indicated a beneficial effect of these products on the intestinal health of domestic animals (Kang *et al.*, 2013); and *Chlorella* is already used as vegan protein source in human nutrition (Panahi *et al.*, 2016; Wells *et al.*, 2017). Critical for vegetarian health, microalgae are a source of vitamin B_12_. Although some green algae appear to be cobalamin autotrophic, often the true source of this vitamin are the associated bacteria (Helliwell, 2017). Various microalgae including *Chlorella* species are sources of carotenoids (Ambati *et al.*, 2018; Sun *et al.*, 2018) and unsaturated fatty acids; both highly in demand in the fish farming industry (Doughman *et al.*, 2007; Winwood, 2013; Kiesenhofer and Fluch, 2018). A vast selection of *Chlorella* food supplements has recently emerged, in which products are often promoted with extensive health benefit claims. For many of these *Chlorella* products no further definition of the species is provided, whereas some products claim to contain *C. sorokiniana*, many *C. vulgaris*, and most of the products with a full species name are designated as *C. pyrenoidosa*. Unfortunately, taxonomists have long abandoned the latter classification (Huss *et al.*, 1999; Champenois *et al.*, 2015). The pertinent database www.algaebase.com lists the name *C. pyrenoidosa* as a ‘homotypic or heterotypic synonym’ and this is just one example of the deplorable situation of *Chlorella* taxonomy.

Attempts to use microalgae for recombinant protein expression have instigated interest in the protein glycosylation potential of these organisms (Mathieu‐Rivet *et al.*, 2014; Yusibov *et al.*, 2016). The green algae *Chlamydomonas reinhardtii* has been shown to partly express smaller oligomannosidic glycans with *O*‐methyl groups and xylose in the plant‐typical β1,2‐linkage to the β‐mannose but also in β1,4‐linkage to an outer arm mannose (Mathieu‐Rivet *et al.*, 2013; Lucas *et al.*, 2020). Markedly, the apparently innocuous Man5 glycan (Man_5_GlcNAc_2_) was shown to exhibit an unusual linear structure that is not a substrate for *N*‐acetylglucosaminyltransferase I (Vanier *et al.*, 2017). Mass spectrometric evidence for N‐glycans with terminal GlcNAc has, however, been obtained for the green alga *Botryococcus* (Schulze *et al.*, 2017). Of the widely used genus *Chlorella*, to date the N‐glycans of only one member of the genus *Chlorella*, *C. vulgaris*, have been characterized. These were shown to contain regular oligomannosidic glycans with up to six *O*‐methyl groups (Mócsai *et al.*, 2018).

During this study on *C. vulgaris* glycans, the requirement for more material instigated the purchase of commercial *Chlorella* tablets. The N‐glycans of this sample (code C‐1, see Table S1) came as a surprise because of the never before seen major matrix assisted laser desorption ionization‐time of flight mass spectrometry (MALDI‐TOF MS) [M+Na]^+^ peak at *m*/*z* 1343.46. Indeed, this C‐1 *Chlorella* product was labelled *C. pyrenoidosa* rather than *C. vulgaris*. Together with the marked observation that species from the same genus were capable of constructing highly different N‐glycans, we realized that the species name of this product as *C. pyrenoidosa* was problematic as this species designation had been abrogated (Champenois *et al.*, 2015) and cannot be found in the PubMed taxonomy database (www.ncbi.nlm.nih.gov/pubmed). The producer of this product unfortunately could not name a representative culture collection strain. The hope of locating a corresponding sample and finally a live organism led us to purchase many more commercial products as well as several culture collection strains previously submitted under the name *C. pyrenoidosa*. The live strains and, even more so, the commercial samples opened a window into an unexpected, fascinating world of hitherto unseen structural variety of N‐glycans.

Here, an initial overview of the spectacular diversity of the structural features of N‐glycans of commercial *Chlorella* products and culture collection strains is presented. Thirteen clearly discernible groups were defined by MALDI‐TOF MS. In several cases, glycans with identical mass were shown to be comprised of quite different N‐glycan structures. To assess the genetic relationships of these samples and to evaluate the validity of the glycan‐based classification, DNA‐based barcoding was undertaken using the rRNA ITS1–5.8S–ITS2 region (Bock *et al.*, 2011b; Hadi *et al.*, 2016). Because of the already enormous amount of data held by this first introduction of the N‐glycan patterns and their characteristic differences, we deliberately refrain from an attempt at closer structural determination with the exception of the GlcNAc‐transferase I product Man5Gn. A detailed structural analysis of two samples (‘Hel’ and ‘Kei’) can, however, be found in a separate work (Mócsai *et al.*, 2020).

## Results

### Establishing glyco‐pattern groups of commercial products

**Figure 1:**
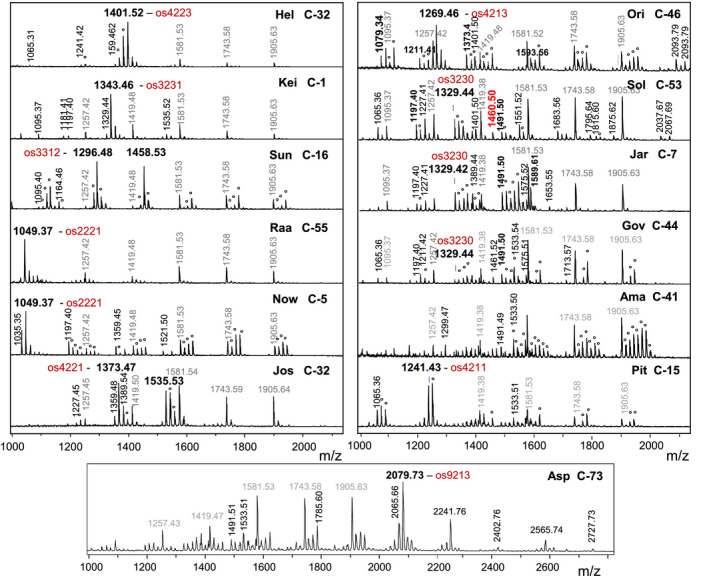
Matrix assisted laser desorption ionization‐time of flight mass spectrometry (MALDI‐TOF MS) spectra of 13 glycan patterns of *Chlorella* products. Values given are the monoisotopic mass of the [M+Na]^+^ ions of underivatized, reducing glycans, the glyco‐group (e.g. ‘Hel’) and the particular product (e.g. C‐32; see Table S1). Oligomannosidic glycans are annotated in grey. Composition of the primary oligo‐saccharide (os) is indicated by the number of hexoses, HexNAcs, pentoses, and methyl groups. A deoxyhexose amounts to a methylated pentose. Peaks resulting from methylation are labelled with an circle.

The N‐glycans of 80 commercial *Chlorella* products and several type collection strains were analyzed by MALDI‐TOF MS, which immediately demonstrated a large variety of glycan patterns and compositions made up of varying numbers of hexoses, HexNAcs, pentoses, and methyl groups (or deoxyhexoses) (Figure 1). The pattern of the initial C‐1 sample was only observed for one other product (C‐54, in which the ‘C’ stands for *Chlorella* followed by an arbitrary number; see Table S1), which could be traced to the same producer. Although 23 products were labelled as *C. vulgaris*, none exhibited a ‘standard’ *C. vulgaris* pattern with nothing else than methylated oligomannosidic glycans. The complexity of many of the glycan patterns after some contemplation resolved into several recurring patterns that will be discussed in this paper. Distinctive glycans were further characterized by MALDI‐TOF tandem mass spectrometry (MS/MS) and chromatography on porous graphitic carbon (PGC) with mass spectrometric detection. PGC is a stationary phase with excellent selectivity for the shape of isomeric glycans (Pabst *et al.*, 2012) and has been previously used to demonstrate that *C. vulgaris* contains the regular Man5 isomer as opposed to the linear version observed with *C. reinhardtii* (Vanier *et al.*, 2017; Mócsai *et al.*, 2018). Monosaccharide analysis of purified compounds was primarily conducted by GC‐MS and ambiguous cases were resolved by reversed‐phase high performance liquid chromatography (RP‐HPLC) of hypermethylated monosaccharides (Windwarder *et al.*, 2016). These experiments substantiated that the N‐glycan patterns of these samples, which were allegedly all derived from *Chlorella* strains, were not just variations in the relative proportions of the very same glycans.

It is certainly feasible that the commercial samples were mixtures of strains. It is common practice, especially in Southeast Asia, to cultivate microalgae in open tanks or ponds, where contaminants cannot be avoided (Goers *et al.*, 2010). It nevertheless seems that, in most cases, a dominant strain was responsible for the characteristic glycan pattern. This notion was supported by analyses of the ITS1–5.8S–ITS2 ribosomal DNA, which is often used for microalgae identification (Bock *et al.*, 2011a; Hadi *et al.*, 2016). Indeed, not all candidates of a glyco‐group yielded an unambiguous sequence read for the primary PCR product. In most glyco‐groups at least one sample gave a readable sequence that was regarded as representative. As this sample had the very same glycan pattern as the other group members, this DNA sequence was taken as representative of the glyco‐group.

The spectra revealed *c.* 100 different mass values that largely have not yet been associated with N‐glycan structures. An obvious next step would be to determine the structures that underlie each of the observed masses. Understandably, this first initial glimpse into the complex‐type N‐glycans of *Chlorella* cannot determine all these several dozen structures. In this paper we will discuss the characteristic and distinctive features of the so‐far identified *Chlorella* glycan patterns, which are summarized in Table 1. The patterns were arbitrarily given a three letter code, whose choice was largely based on phonetic elegance (Tables 1 and S1).

**Table 1:**
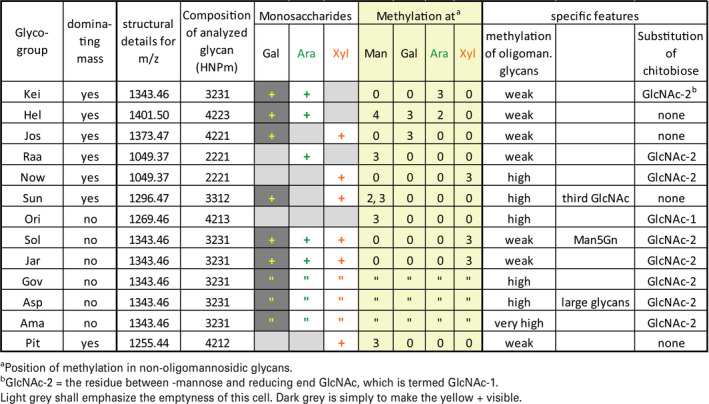
Summary of structural features of the 13 *Chlorella* glyco‐groups. Composition of glycans is given as the number of hexoses, *N*‐acetylhexosamines, pentoses, and methyl groups (whereby a deoxyhexose would occur as pentose plus methyl group). Mass values represent sodium adducts of underivatized reducing glycans. Structural identity of the *m*/*z* = 1343.46 glycans in ‘Jar’, ‘Gov’, ‘Asp’, and ‘Ama’ was deduced from their identical elution on a PGC column of this and several other glycans of these samples

### Glycan pattern ‘Kei’

**Figure 2:**
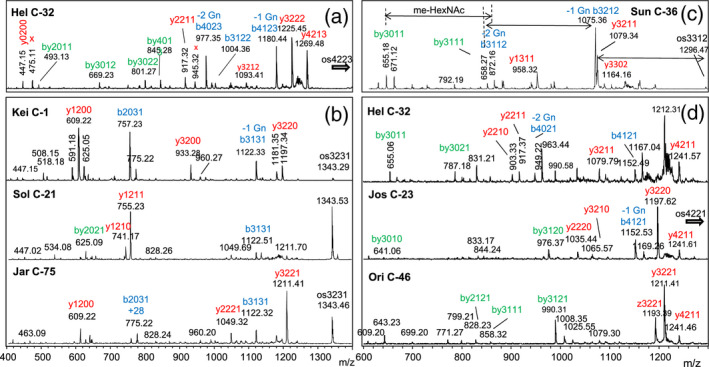
MALDI‐LIFT spectra of selected glycans of the ‘Hel’, ‘Kei’, ‘Sol’, ‘Jar’, ‘Sun’, ‘Jos’, and ‘Ori’ glycan groups. Panels (a–d) show spectra obtained from the parent masses 1401.5, 1343.5, 1296.4, and 1373.5 respectively. Fragments were labelled according to the number of hexoses, HexNAcs, pentoses, and methyl groups, whereby y‐fragments are shown in red, b‐fragments in blue, and by‐fragments in green. Fragments indicating the loss of the first and second GlcNAc of the chitobiose core are indicated by ‘‐1 Gn’ and ‘‐2 Gn’. In (c), mass increments for methylated HexNAc are shown by arrows.

**Figure 3:**
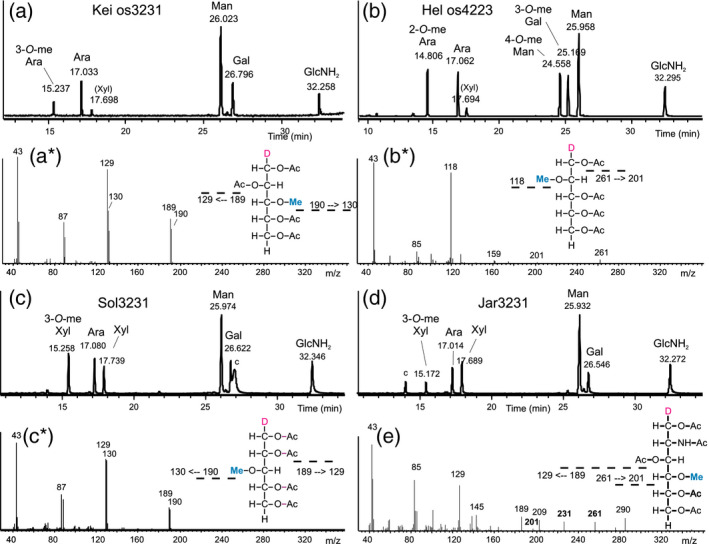
Monosaccharide analysis of selected glycan groups. Panels (a–d) show the results of GC‐MS analysis of the highly enriched glycans with *m*/*z* = 1343.5 from Kei‐C‐1, 1401.5 from Hel‐C‐32, and 1343.5 from both Sol‐C‐39 and Jar‐C‐75 respectively. (a*, b*, and c*) are the fragment spectra of peaks at 15.2, 14.8, and 15.2 min showing 3‐*O*‐methyl‐arabinose and 2‐*O*‐methyl‐arabinose and 3‐*O*‐methyl‐xylose respectively. Ambiguity between 3‐*O*‐methyl‐arabinose and 3‐*O*‐methyl‐xylose was resolved by hypermethylation (Figure S1). Panel (e) depicts the spectrum of 4‐*O*‐methyl‐*N*‐acetylglucosamine in sample Sun C‐36 (see also Figure S8).

This group exhibited a [M+Na]^+^ peak at *m*/*z* 1343.46 that exceeds the well known MMXF^3^ (Man_3_GlcNAc_2_XylFuc) glycan that occurs in all land plants by exactly the mass of a pentose. Based on observations of N‐glycans with two xylose residues in *Chlamydomonas reinhardtii* (Mathieu‐Rivet *et al.*, 2013), a premature assumption was made that the glycan was the known MMXF^3^ configuration plus an additional xylose, which may possible sit at the second GlcNAc (Levy‐Ontman *et al.*, 2011). The MALDI‐LIFT‐TOF/TOF spectrum of *m*/*z* = 1343.4 (os3231 = Hex3HexNAc2Pen3met1, whereby a deoxyhexose would be annotated as Pen1met1) in sample C‐1 already brought a surprise. The loss of 221 mass units (y_1_‐ion) clearly indicated that this glycan was not substituted at GlcNAc‐1, as is the case in ‘higher’ plant N‐glycans (Figure 2) (Strasser, 2016) whereas the LIFT spectrum did not rule out substitution of the second GlcNAc. The total lack of a fragment without two GlcNAc residues, together with the presence of the complementary y‐ and b‐ions at 609.2 and 757.2 Da, implied the substitution of GlcNAc‐2, possibly by galactose (Figure 2). The *m*/*z* = 1343.4 peak was isolated by preparative hydrophilic interaction chromatography (HILIC)‐LC and was found to contain neither xylose nor fucose but rather arabinose, 3‐*O‐*methyl‐arabinose, and galactose (Figure 3). Note that this os3231 N‐glycan lacked terminal GlcNAc, but nevertheless contained galactose. Therefore, this sample contained an N‐glycan with a very new and unusual structure. The exact structure of this glycan was determined by nuclear magnetic resonance (NMR) as detailed elsewhere (Mócsai *et al.*, 2020).

### Glycan pattern ‘Hel’

These samples contained a main glycan peak at *m*/*z* = 1401.5 (os4223) preceded by peaks with 14 Da mass differences, an indication of methylation. The first and smallest of these peaks had a mass of 1373.5 and an os4221 composition, which also occurred in other groups (‘Jos’ and ‘Ori’). The LIFT spectrum of the main peak of Hel‐C‐32 contained strong b‐ions arising from the consecutive loss of two GlcNAc residues and hence indicated an unsubstituted chitobiose core (Figure 2). Monosaccharide analysis of the isolated *m*/*z* = 1401.5 peak revealed a composition of three mannoses (one being 4‐*O*‐methylated), two arabinoses (one being 2‐*O*‐methylated) and a 3‐*O*‐methyl galactose in addition to two GlcNAc residues (Figure 3). These data argue for an overall architecture fundamentally different from that of the major Kei‐C‐1 glycan.

This glycan pattern was also found in type collection strains seen as prototypes of the species *C. sorokiniana* (discussed later in this paper).

### Glycan pattern ‘Jos’

The major glycan in Jos‐C‐23 shared an *m*/*z* = 1373.5 with the ‘Hel’ samples, which opened the possibility that the ‘Jos’ pattern differed only by relative abundance of the same components. The LIFT spectrum of this peak, however, exposed clear differences (Figure 2d). An even clearer distinction came from monosaccharide analysis of this os4221 from Jos‐C‐23, which contained two xylose and a 3‐*O*‐methyl galactose residue in addition to mannose and GlcNAc (Figure S2). The sample Jos‐C‐24 was included in this small group due the occurrence of *m*/*z* = 1373.5, even though the dominant methylation of oligomannosidic N‐glycans and the occurrence of peaks at *m*/*z* = 1211.3, 1414.4, and 1428.4 may argue for a separate group that may be called ‘Wel’ if this pattern turns out as arising from one alga strain rather than just being a mixture.

### Glycan pattern ‘Ori’

‘Ori’ samples also contained a rather large peak of *m*/*z* = 1373.5 (os4221) in addition to a dominant *m*/*z* = 1269.5. The *m*/*z* = 1373.5 peak of Ori C‐46 might at first glance have resulted from contamination of this product with ‘Jos’ algae during the production stage. However, the LIFT spectrum clearly argued against this assumption (Figure 2d). The prototypical *m*/*z* = 1269.5 peak (os4213) contained 3‐*O*‐methyl mannose and emerged only in PNGase A digests but not by PNGase F, which does not cut N‐glycans with core‐1,3‐linked fucose (Tretter *et al.*, 1991). This may well relate to the fact that the reducing GlcNAc loss of 221 mass units only occurred consecutive to loss of – in this case – 146.1 mass units (Figure S3). Permethylation of a HILIC fraction rich in *m*/*z* = 1269.3 determined that the 146.1 increment was caused by a deoxyhexose (Figure S3), which materialized as fucose by GC‐MS as well as by LC‐MS (Figure S4).

It may be added that Ori C‐46 also contained an *m*/*z* = 1211.4 that: (i) was not preceded by a 1197.4 peak (see below); (ii) was released by PNGase F (unlike the MMXF^3^ plant glycan with the same mass); and (iii) had a LIFT spectrum different from both MMXF^3^ and the isobaric glycan from Sol‐C‐21 (Figure S5).

DNA experiments showed that none of the products of the ‘Ori’ group was pure enough to consider that it originated from one strain (see section on ‘DNA‐based classification of *Chlorella* products and strains’). This aroused suspicion that some samples of the ‘Ori’ group stemmed from more than just one algal strain and was related to the observation of various larger glycans in just some of these samples (C‐28, C‐46, C‐82; see Data S1). ‘Ori’ may thus be a catchment basin that will eventually be split up into further glyco‐groups.

### Glycan patterns ‘Raa’ and ‘Now’

The eye‐catching trait of both of these patterns was the dominant *m*/*z* = 1049.4 peak. The oligomannosidic glycans were not methylated with the exception of sample C‐5. This turned out to be more than just a marginal variation, as ESI‐MS/MS revealed a different assembly of the isobaric main glycans (Figure S6). In fact, while ‘Raa’ *m*/*z* = 1049.4 contained arabinose and methylated mannose, the ‘Now’ isobar had methylated xylose (Figure S7). The MALDI‐LIFT peaks at 579.2 and 828.3 mass units occurred in both Raa‐C‐60 and Now‐C‐5 and indicated that, in both samples, a pentose was linked to the second GlcNAc. However, the pentose was an arabinose in the ‘Raa’ sample and a xylose in the ‘Now’ sample.

### Glycan patterns ‘Sun’

This further glyco‐group with just a few dominating masses was characterized by large peaks with masses indicating the presence of a third *N*‐acetylhexosamine that rendered this group unique (Figure 1). The LIFT spectrum of *m*/*z* = 1296.5 in Sun C‐16 showed a free reducing GlcNAc (loss of 211.1 mass units). A fragment with *m*/*z* = 1079.1 indicated loss of a methylated GlcNAc and therefore the 872.1 fragment pointed at an entirely unsubstituted chitobiose core (Figure 2c). Monosaccharide analysis of the *m*/*z* = 1296.5 revealed it to contain 4‐*O*‐methyl GlcNAc, xylose, galactose and three versions of mannose (2‐*O*‐methyl‐, 3‐*O*‐methyl, and unmethylated mannose) (Figures 3e and S8). The number of methyl groups and hexoses in this os3312 necessitated this glycan to be a mixture of variants with different mannose methylations. Methyl GlcNAc has, to our knowledge, not been found before.

The only two samples in this group differed from each other by the intensity of the *m*/*z* = 1003.4 peak (o3205) and the occurrence of *m*/*z* = 1884.7 (os5332) (see section on ‘Spectra collection’ at the end of Data S2).

### Glycan pattern ‘Sol’

**Figure 4:**
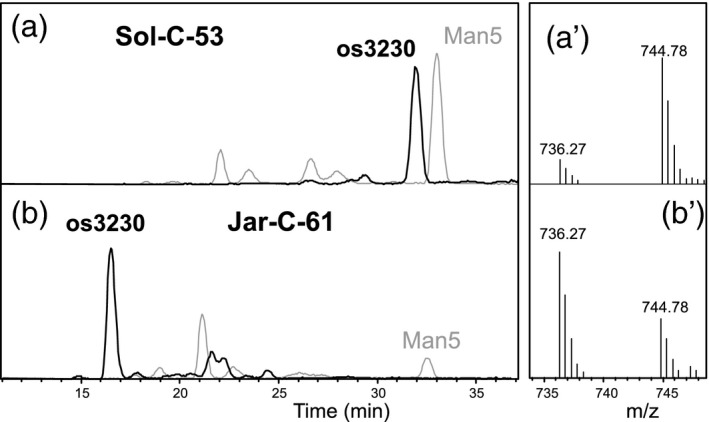
Elution behaviour of the pentose‐containing glycan os3230 on porous graphitized carbon‐liquid chromatography‐electrospray ionization‐mass spectrometry (PGC‐LC‐ESI‐MS). Panels (a) and (b) show the extracted ion chromatograms for *m*/*z* = 736.2 (os3230, doubly charged) and *m*/*z* = 1237.4 (os5200) for Sol‐C‐53 and Jar‐C‐61 respectively. For os5200, the later eluting peak represents the regular Man5 structure. It must be added that the elution profiles for this glycan in the ‘Gov’, ‘Ama’, ‘Asp’, and ‘Pit’ glyco‐groups resembled that of the ‘Jar’ sample (Figure S9). Panels (a´) and (b´) demonstrate the differing ratios of [MH_2_]^2+^ and [MHNH_4_]^2+^ ions.

More than half of all samples analyzed exhibited a highly complex N‐glycan pattern characterized by series of glycans with more or less *O*‐methyl groups starting at *m*/*z* = 1197.4, 1329.4 and 1491.5, which corresponds to os3220, os3230, and os4230 respectively. An obvious feature that distinguished these samples was the methylation of oligomannosidic glycans (Figure 1). Another, somewhat hidden, distinction was the presence of a peak at *m*/*z* = 1460.5. Distinguishing the ‘Sol’ group, that is samples with Man5Gn, from the otherwise similar ‘Jar’ group turned out to be germane, whereas the similarity of the very obvious pentose series was deceptive, as shown in this paper. The monosaccharide composition of os3231 likewise indicated the identity of the ‘Sol’ and the ‘Jar’ group, as both Sol‐C‐39 and Jar‐C‐75 contained galactose, arabinose, xylose, and 3‐*O*‐methyl xylose (Figure 3). However, the MALDI‐LIFT spectra of os3231 deviated considerably and both samples obviously exhibited structures that deviated from the isobaric ‘Kei’ glycan (Figure 2). A common feature of all three os3231 spectra was the absence of a peak that would have indicated an unsubstituted GlcNAc‐2. The difference between ‘Sol’ and ‘Jar’ was corroborated by the PGC retention times (Figure 4). Therefore, the *m*/*z* = 1329.4 glycans in these groups are striking examples of structural differences despite identical numbers of hexose, pentose, and methyl residues. Noteworthy, samples from the ‘Gov’ and ‘Ama’ glyco‐group gave exactly the same PGC elution profiles as ‘Jar’ (Figure S9). 

These samples also contained *m*/*z* = 1211.3 glycans as in the common plant N‐glycan MMXF^3^. LIFT spectra of the three isobaric glycans displayed large differences and this ruled out structural identity (Figure S5).

The characteristic feature of the ‘Sol’ group, the peak at *m*/*z* = 1460.5, could be the very Man5Gn glycan that was the switch point from oligomannosidic to complex‐type glycans. To scrutinize the nature of this peak, core α1,6‐fucosyltransferase was utilized, assuming it to be an ultimately rigorous examiner of the structure of this compound. An HILIC‐enriched fraction of Sol‐C‐3 was incubated with human core α1,6‐fucosyltransferase in the presence or absence of GDP‐fucose (Figure S10). The peak observed at *m*/z 1460.5 was converted to a product at *m*/*z* 1606.5. At least for the ‘Sol’ glyco‐group, this observation therefore proved the existence of relevant parts of the common complex‐type N‐glycan biosynthesis machinery in *Chlorella*.

### Glycan pattern ‘Jar’

This pattern was seemingly identical to the ‘Sol’ glycan pattern, apart from the lack of *m*/*z* = 1460.5 (Figure 1). The 1343.5 = os3231 glycan from Jar‐C‐75 contained galactose, arabinose, xylose, and 3‐*O*‐methyl xylose, just like that from Sol‐39. PGC‐LC and LIFT spectra, however, revealed fundamental differences (Figures 3 and 4).

### Glycan patterns ‘Gov’, ‘Ama’, ‘Asp’, and ‘Pit’

These four patterns may be seen as subtypes of ‘Jar’ as they all contained more or less prominent peaks of *m*/*z* = 1329.5, 1343.5, 1491.5, and their methylated variants. Glycans of these ‘Jar’ type series from all these four groups behaved just like that from ‘Jar’ samples on a PGC column including the distinctive low ability to form ammonium adducts and LIFT spectra (Figure 4). They exhibited, however, very obvious, additional traits (Figure 1). At this point, we should re‐emphasize that the glycans observed in these products may indeed be derived from different algal strains. However, each profile exhibited features that were characteristic of a specific *Chlorella* strain, irrespective of possible additions from another strain.

The ‘Gov’ pattern differed from ‘Jar’ by the pronounced O‐methylation of oligomannosidic N‐glycans with up to three methyl groups per glycan. In contrast, the oligomannosidic N‐glycans of the ‘Ama’ group contained up to seven methyl groups.

Products that exhibited an extremely high methylation with up to seven methyl groups per glycan were collected in the ‘Ama’ group.

Several products contained large peaks for glycans of *m*/*z* = 2079.7, 2241.8, 2565.7, and methylation variants thereof. The mass values of these ‘Asp’ group glycans can be translated into compositions of os9213, os[10]213, and os[12]213. The presence of methyl groups may be taken as a hint that these large glycans were of algal, and not fungal, origin.

The ‘Pit’ glyco‐group distinguished itself from the above samples by dominant characteristic peaks at *m*/*z* = 1241.4 and 1255.4, which translated into compositions of os4211 and os4212. These small glycans contained methylated mannose, xylose, no galactose, and an unsubstituted chitobiose and are therefore distinguished from other xylose‐containing glycans (Figure S11).

Glycans such as *m*/*z* = 1329.5 and 1491.5 of all these four groups discussed in this paper behaved just like those from ‘Jar’ samples on a PGC column (Figure S9).

### Orphan glycan patterns

A few samples and live strains gave glycan patterns that did not fit nicely into any of the above categories (Figure S12). As these samples came as singular cases only, we did not assign further group names. These ‘orphans’, however, are a reminder that the current study almost certainly describes only a part of the fascinating variety of N‐glycan structures in the *Chlorella* clade.

### DNA‐based classification of *Chlorella* products and strains

Can a single genus give rise to so many diverse N‐glycans? To answer this question, we selected the ITS1–5.8S–ITS2 rRNA gene to serve as genetic barcode and performed phylogenetic analysis as described previously (Bock *et al*., 2011a,b; Hadi *et al.*, 2016). PCR primers were constructed to bind to two highly conserved sections flanking the ITS1–5.8S–ITS2 DNA strand. The design enabled us to amplify ITS1–5.8S–ITS2 rDNA from almost every *Chlorella* species, as well as other green algae. The resulting ITS1–5.8S–ITS2 fragment was rather short (~850 bp) in length and highly variable in sequence, and was therefore considered a useful target to investigate genetic variation within closely related species.

**Figure 5:**
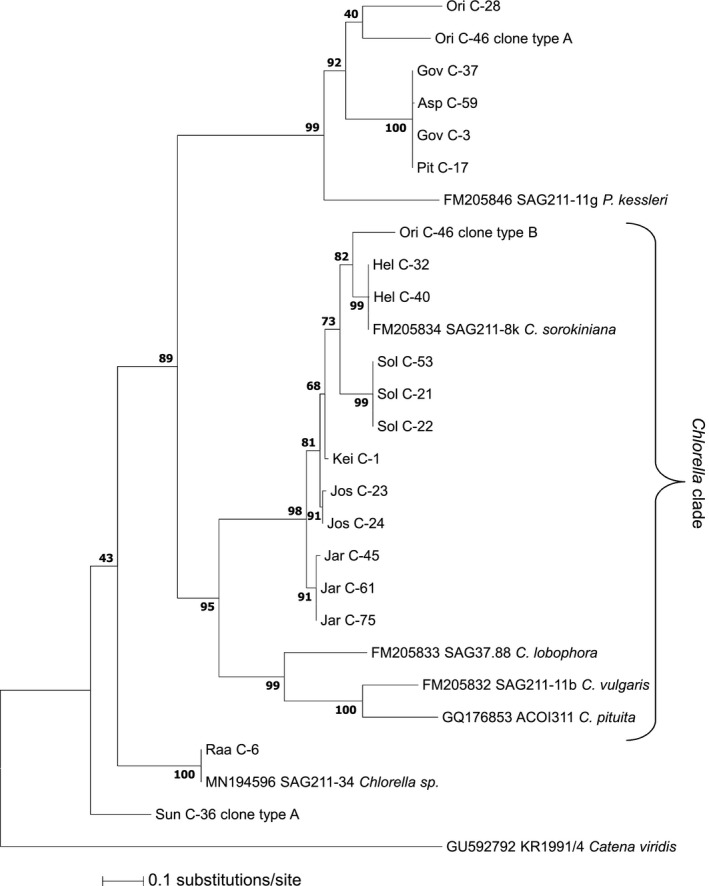
Molecular phylogeny of the ITS1–5.8S–ITS2 rDNA sequences. Relevant culture collection strains and commercial *Chlorella* products were compared with *Catena viridis* as the outgroup. The tree was rooted on midpoint. Numbers at nodes represent normalized bootstrap values of 1000 replicates in per cent. GenBank accession numbers and culture collection strain numbers are given for all database entries. Product sequences are documented in Data S1.

Extraction of genomic DNA proved to be a difficult task from some of the commercial products. In many cases low yield, high fragmentation of the extracted genomic DNA or ambiguous sequencing results hindered the experiments. Only 19 products could be included in the downstream analysis. Nevertheless, PCR products of candidates from most of the major glyco‐groups were obtained. Here, 17 PCR products gave unambiguous sequencing results, indicating a strong prevalence for one single species per product. However, direct sequencing of the PCR products of samples Sun C‐36 and Ori C‐46 resulted in chromatograms with superimposed peaks in the ITS1 and ITS2 regions, indicating a mixture of genes from different species. PCR fragments were therefore transformed into *E. coli* and sequenced again from single colony picks. Sun C‐36 resulted in two different types of sequences, termed type A and type B. Out of the 15 clones, the majority (93.3%) were of sequence type A (C‐36‐A) and 6.7% were type B (C‐36‐B), confirming the presence of a mixed population. The strong dominance of the Sun C‐36‐A DNA sequence implied that the characteristic structure with three GlcNAc residues found in this sample, originated from this population. BLAST results of C‐36‐A showed that this species may be related to *Micractinium pusillum* (Table S2), a species within the Chlorellaceae family, but relatively distant from *Chlorella.* For Ori C‐46, three distinct sequences of types A, B, and C could be identified with a rather balanced ratio of 1.5 to 1.2 to 1 respectively. The major sequence Ori C‐46‐A is expected to be a member of the ‘Ori’ group, because of its close sequence relationship to Ori C‐28 in the phylogenetic tree (Figure 5). The second sequence type of this sample (Ori C‐46‐B) showed sequence similarities to the *C. sorokiniana* type strain (SAG211‐8k). The minor clones Sun C‐36‐B and Ori C‐46‐C both showed sequence similarity to *Scenedesmus* species (Table S2). This could be a sign of contamination by indigenous green algae on the production sites. Additionally, both sequences clustered well outside the *Chlorella* clade in an initial phylogenetic analysis and were removed from the alignment as a result. Overall, the ITS1–5.8S–ITS2 DNA sequences and the performed BLAST searches revealed all commercial products included in the phylogenetic analysis to essentially consist of organisms that belong to the Chlorellaceae family even though some samples, especially the ‘Raa’ group, appear as rather remote from the *Chlorella* clade (Figure 5).

The phylogenetic tree constructed with ITS1–5.8S–ITS2 fragments displayed substantial accordance with the grouping obtained by analysis of N‐glycans (Figure 5). The glyco‐groups ‘Hel’, ‘Kei’, ‘Jos’, ‘Jar’, and ‘Sol’ and clone Ori C‐46‐B showed a close relationship to the type strain *C. sorokiniana* (SAG211‐8k) despite rather divergent glycan patterns. Glyco‐groups ‘Ori’, ‘Gov’, ‘Asp’, and ‘Pit’ (but surprisingly not ‘Jar’) formed a clade separate from the *Chlorella* clade. A BLAST search of these samples (Table S2) revealed close homology to various *Dictyosphaerium* species, which are part of the Chlorellaceae family.

‘Raa’ and the culture collection strain SAG211‐34 had an identical glycan pattern as well as an identical ITS1–5.8S–ITS2 sequence. The rDNA sequence of the SAG211‐34 live strain – currently falsely termed *Chlorella sorokiniana* in the SAG database – has been submitted to the GenBank database under accession number MN194596.

### N‐glycan patterns of strain collection *Chlorella* species

With the aim of finding strains with glycan patterns matching those of commercial products, for example that of sample C‐1, we ordered and grew a number of strain collection lines recently assigned as *C. pyrenoidosa* (and now *C. vulgaris*, *C. sorokiniana* or just *Chlorella* sp.). As mentioned above, the glycan patterns of *C. sorokiniana* lines SAG 211‐8k, SAG 211‐32, and UTEX 1230 perfectly matched those of products placed in the ‘Hel’ group (Figure S13).

MALDI‐TOF and TOF/TOF MS revealed kinship of lines FACHB‐31, 211‐31, and 211‐34 with samples from the ‘Raa’ group (Figure S14). The latter two lines contained additional peaks such as glycans with three HexNAc residues (*m*/*z* = 1884.7 and 2046.71 = os 5332 and os6332 respectively).

With regard to the obvious question of how far growth conditions might influence the glycan patterns or even structures meant that results of preliminary experiments are mentioned here. First, N‐limitation did not change the pattern of *C. vulgaris* SAG 211‐11b. Second, continuous cultivation for over 3 years did not result in a change in SAG 211‐11b and SAG 211‐8k. Most markedly, no change was observed when these two strains were grown heterotrophically in the dark with glucose as the energy source.

## Discussion

Commercial samples of *Chlorella* displayed an astounding plethora of different mass patterns, assembly details and, hence, structures of asparagine‐linked glycans. A combination of MALDI‐TOF MS and MS/MS, component analysis, and chromatography revealed numerous previously unknown and unidentified features. These included the occurrence of arabinose and galactose on glycans without antennary GlcNAc, and various types of *O*‐methylation including that of *N*‐acetylglucosamine (Table 1). Identical masses differed in composition and structure to the point that the N‐glycan structural diversity appeared to be unparalleled in land plants or higher animals. Thirteen clearly different glycan patterns could be identified, but only two of these – ‘Hel’ and ‘Raa’ – could be matched to culture collection strains. Some of the glycan patterns, certainly the ‘Ori’ glyco‐group, may be derived from different algal strains. The general observation of characteristic traits was not devalued by this caveat, which will be resolved in future research.

Ranging from fish, birds, mammals through to humans, the N‐glycans from vertebrate animals differ by only a few details. These include GlcNAc‐transferase VI (Brockhausen *et al.*, 1989), α1,3‐galactosyltransferase (Galili, 2005), and the ability to form *N*‐glycolneuraminic acid (Varki, 2001). Land plants from ferns to gymnosperms to mono‐ and dicotyledonic angiosperms all exhibit the same N‐glycan architecture (Lerouge *et al.*, 1998; Strasser, 2016). By far, the microalgal species *Chlorella* alone exceeded the N‐glycan diversity of these large groups of organisms. This paper is not the forum to discuss whether land plants and microalgae have a common ancestor that later resulted in diversification of N‐glycosylation or whether land plants are the descendants of a particular microalgal glyco‐type that ‘hit the evolutionary jackpot’ by being able to develop into embryophytes. One hint may come from the presence of substantial amounts of the Man5Gn structure in the ‘Sol’ group. This group contains the structure of the common nexus of complex‐type N‐glycan processing, which was substantiated by qualifying as a substrate for core α1,6‐fucosyltransferase. Traces of the product and homologue genes for the key enzyme of N‐glycan processing have already been observed in *C. vulgaris* (Mócsai *et al.*, 2018). Nevertheless, this situation could still be the result of horizontal gene transfer and/or the echo of a common origin of eukaryotic N‐glycosylation. Strong support, however, can be seen in the high similarity between the putative *Chlorella sorokiniana* GlcNAc‐transferase I (A0A2P6TDC6) and the C‐terminal half of citrus and coral orthologues (A0A067FLW9 and A0A2B4RWY4; error probabilities of 10^−94^ and 10^−68^ respectively). Of note, it has been reported that the genome of *C. reinhardtii* lacks a corresponding gene and a BLAST search returned no match similarities (Vanier *et al.*, 2017).

This work will not speculate on repercussions of its findings for taxonomy of *Chlorella*‐like microalgae. What can be deduced with certainty, however, is that the algal N‐glycan structures do provide solid criteria for strain characterization and probably taxonomy. In most cases, the distinction between glyco‐groups is unambiguous and it appears conceivable to assume that the acquisition of certain glycosyl or methyl transferases is by far a greater evolutionary step and a less haphazard event than the random exchange of a base in the inoperable internal transcribed spacer (ITS) rDNA. The need for additional criteria for strain characterization is exemplified by the essentially random naming of products as either *C. vulgaris* or *C. pyrenoidosa* (Table S1).

Recently, our group reported the N‐glycan structures of *C. vulgaris*‐type strains. These exclusively contained oligomannosidic structures with 0–5 methyl groups (Mócsai *et al.*, 2018). One‐quarter of the commercial samples were designated as *C. vulgaris*, however none presented an N‐glycan pattern that completely matched the *C. vulgaris*‐type strains. At least for the products that showed some similarity, it was possible to speculate that the chosen growth conditions led to a temporal, or ultimate, silencing of the genes responsible for synthesizing ‘complex‐type’ glycans. The clearly differing ITS rDNA sequences, however, negated this idea. Notably, ITS rDNA sequences and glyco‐groups showed a very high degree of consistency, even in this very initial and cursory trial. Therefore, DNA‐based tests can serve as cost‐effective proxies for glycan pattern analyses in the context of product quality assurance. Nevertheless, growth conditions could influence the glycan pattern. Growing *Chlorella* strains in nitrogen‐depleted, heterotrophic, or photoautotrophic conditions, however, did not cause any perceivable change in their glycan pattern.

Notably, of the 80 products investigated, 23 each were named *C. vulgaris* or *C. pyrenoidosa*, but these names were randomly distributed over all glyco‐groups (Table S1). In line with this, no DNA homologues to *C. vulgaris*‐type strain SAG 211‐11b were found in any of the samples. To understand this unclear situation, it has to be mentioned that, in many algae culture collections, *Chlorella vulgaris* is a name given to many different, mostly unreviewed, isolates for which no sequence information is available.

Undoubtedly, N‐glycan patterns will prove useful for strain classification, product characterization and health‐relevant issues such as quality control and in tracking the origin of *Chlorella* products. The methodology for this assessment will require simplification, possibly through the use of antibodies or the identification of specific genetic markers. We postulate that glycan patterns will constitute an important pillar of microalgal differentiation in addition to classical ITS‐based taxonomy (Bock *et al.*, 2011a) also relevant to the extended group of genetic markers (Zou *et al.*, 2016).

The phylogenetic tree constructed with the help of ITS1–5.8S–ITS2 rDNA sequences basically reflected the grouping obtained via glycan patterns (Figure 5), thereby substantiating the significance of N‐glycan analysis for the phylogenic characterization of *Chlorella* and presumably other microalgae. This forecast arose from the finding of fucose in *Scenedesmus*‐related samples, which in turn supports the notion of a large phylogenetic distance between these and the *Chlorella‐*like samples.

Finally, the question of why this variety of structures and underlying glyco‐enzymes could evolve in *Chlorella* remains to be answered. *Chlorella*, or at least the recently genome‐sequenced *C. variabilis*, is a haploid organism with no known diploid stage (Blanc *et al.*, 2010; Eckardt, 2010). In an analogous study on *Ostreococcus* (Grimsley *et al.*, 2010), the presence of genes that in other organisms affected meiosis and disassembly of the gamete cell wall (a pre‐requisite for gamete fusion) argued for occasional sexual reproduction or the so‐called ‘cryptic’ sex (Eckardt, 2010). A broad survey of meiotic genes in Trebouxiophyceae (the class including Chlorellaceae) genomes corroborated the idea that many of these algae can adopt a diploid phase, for example *Chlamydomonas* (Fucikova *et al.*, 2015). Our speculation is that, in the natural environment, cell surface glycoproteins may be one mechanism that aids recognition of suitable partner cells among the myriad of other unicellular, often closely related, organisms. If this indeed proves to be the case, microalgal N‐glycans would thus be a splendid example for the widely appraised, but rarely realized, potential of complex oligosaccharides to act as highly versatile markers for recognition.

## Experimental Procedures

Live *Chlorella* strains were obtained from the Culture Collection of Algae at the University of Göttingen, Germany, http://sagdb.uni‐goettingen.de, the Culture Collection of Algae at the University of Texas in Austin, http://utex.org) or, for FACHB‐31, from the Freshwater Algae Culture Collection at the Institute of Hydrobiology, Wuhan, China (http://algae.ihb.ac.cn/english/Cultrues.aspx). Live strains were cultured photoautotrophically as described previously (Mócsai *et al.*, 2018). Commercial *Chlorella* preparations were purchased from retailers as specified in Table S1.

### Preparation and MALDI‐TOF analysis of N‐glycans

To a 20% slurry of *Chlorella* powder or ground tablets in 5% formic acid/pepsin (Sigma‐Aldrich, www.sigmaaldrich.com) was added an enzyme to substrate ratio of 1:1000, as in previous studies (Grass *et al.*, 2011; Mócsai *et al.*, 2018). Upon incubation at 37°C, the slurry was centrifuged and the supernatant was applied to a column filled with Dowex 50WX2 (Sigma‐Aldrich) in the H^+^ form equilibrated with 2% acetic acid. The binding fraction was eluted with high molarity ammonium acetate buffer, carbohydrate‐containing fractions (orcinol method) were pooled, concentrated, and subjected to gel filtration on Sephadex G25 fine medium. Glycopeptide fractions were digested with either PNGase F (Roche, custombiotech.roche.com) or PNGase A (Europa Bioproducts, www.europa‐bioproducts.com). The digests were once more applied to a Dowex 50WX2 column, but now the flow through containing the released glycans was collected. A final polishing was obtained by passing the aqueous samples over a small bed of C18 resin. Diluted glycan preparations were spotted onto a MALDI target plate and dried. A 2% solution of 2,5‐dihydroxybenzoic acid in 50% acetonitrile was added and the droplets were quickly dried in a vacuum desiccator to obtain small uniform crystals. Spectra were acquired with an Autoflex MALDI (Bruker, www.bruker.com) in positive reflectron mode. All spectra were re‐calibrated using oligomannosidic glycans as internal standards. Fragment spectra were obtained by laser induced fragmentation in LIFT mode.

### Chromatographic separation of glycans

Reducing N‐glycans were fractionated on a TSK‐amide 80 column (Tosoh Bioscence GmbH, www.tosohbioscience.com) as described (Grass *et al.*, 2011), except that underivatized glycans were used. Therefore, fractions were analyzed for glycans by MALDI‐TOF MS.

For analytical purposes, the glycans were reduced with NaBH_4_, desalted by passage over graphitized carbon cartridges (ThermoFisher Scientific, www.thermofisher.com) and subjected to LC‐ESI‐MS with a PGC column (0.32 μm × 150 mm) operated by an ultimate rapid separation LC (RSLC) system (ThermoFisher Scientific) connected to a Maxis 4 G Q‐TOF MS (Bruker) or an amaZone ion trap (Bruker), operating in data‐dependent acquisition mode (Pabst *et al.*, 2012). The PGC column was eluted with 65 mm ammonium formate at pH 3.0 and a gradient from 8 to 60% acetonitrile in 50 min at a flow rate of 6 µl min^−1^ at ambient temperature.

### Composition analysis

Glycans were hydrolyzed for 4 h at 100°C with 4 m trifluoroacetic acid. If pentoses were the target, 2 m acid was used. For gas chromatographic analysis, monosaccharides were reduced with NaBD_4_ (or NaBH_4_) and analyzed by GC‐MS as described (Mócsai *et al.*, 2018). Partially methylated monosaccharide standards were prepared by supplying limiting amounts of methyl iodide for methylation using NaOH (Windwarder *et al.*, 2016).

For HPLC analysis, monosaccharides were labelled with 2‐aminobenzoic acid (AA) (Anumula, 1995; Windwarder *et al.*, 2016) and separated on a core‐shell reversed‐phase column (Windwarder *et al.*, 2016).

For sugars, when GC‐MS analysis did not unequivocally identify the respective epimer (e.g. in the case of a 3‐*O*‐methyl‐arabinose and 3‐*O*‐methyl‐xylose with essentially identical elution times or for *N*‐acetylhexosamines), the epimeric nature of methyl sugars was determined using hypermethylated anthranilic acid‐labelled sugars (Windwarder *et al.*, 2016).

### DNA extraction and ITS1–5.8S–ITS2 rDNA sequencing

Total genomic DNA was extracted from 30 mg dry algal powder or fresh cells for in‐house cultivated SAG 211‐34 using a QIAGEN DNeasy^®^ Plant Mini Kit (Qiagen, www.qiagen.com) according to the producer’s instructions. The ITS1–5.8S–ITS2 gene was amplified by PCR reaction with primers binding to the flanking regions of 18S and 26S rRNA genes, resulting in a DNA fragment of *c.* 950 bp. These primers were designed to bind to all published ‘*Chlorella*’ sequences including *C. vulgaris*, *C. sorokiniana*, *C. miniata*, *C. variabilis*, *C. rotunda*, *C. pituita*, *C. lewinii*, *C. luteoviridis*, but were found to bind to other green algae as well (e.g. *Scenedesmus*, *Parachlorella*, *Pseudochloris*, and *Auxenochlorella*). The sequences of the primers were as follows 5′→3′ TGCCTAGTAAGCGCAAGTCA (forward) and 5′→3′ TTCCTCCGCTTATTGATATGC (reverse). All PCR reactions were performed using Accu*Taq* LA DNA polymerase (Sigma), according to the manufacturer’s instructions, in a total volume of 20 µl using 1.5% DMSO. Thirty cycles were performed, each consisting of a denaturation step of 45 sec at 95°C, an annealing step of 45 sec at 52°C and an extension step of 120 sec at 72°C. Thermocycling was always preceded by a denaturation step of 180 sec at 95°C and followed by an extension step of 7 min at 72°C. After agarose gel electrophoresis, the PCR products were purified using the Illustra GFX PCR DNA and Gel Band Purification Kit (GE Healthcare, www.gelifesciences.com). Sanger sequencing service was provided by Microsynth AG (Switzerland, www.microsynth.ch/). Sequencing results obtained from the respective forward and reverse primer pairs were aligned, generating nucleotide sequences that can be found in the Supporting Information. When required, PCR fragments were ligated into pVT‐Bac‐His, and transformed into chemically competent *E. coli* Top10 cells, which were grown on selective agar plates. For each sample, 15 single colonies were picked, cultured overnight in 5 ml Luria–Bertani (LB)/ampicillin medium at 37°C and their plasmid DNA was extracted using a NucleoSpin^®^ Plasmid Kit (Macherey‐Nagel, www.fishersci.co.uk/gb/en/brands/IFXY1CL7/macherey‐nagel.html) following the manufacturer’s recommendations. In these cases, plasmid DNA was sent for Sanger sequencing. Sequencing results for the collection strain SAG 211‐34 were deposited in GenBank under the accession number MN194596.

### Phylogenetic analysis

All acquired ITS1–5.8S–ITS2 nucleotide sequences were aligned together with reference sequences from GenBank annotated as *Chlorella* species, using the MAFFT algorithm E‐INS‐i (Katoh and Standley, 2013). The corresponding DNA region of *Catena viridis* (GenBank accession: GU592792), a member of the *Chlorellaceae* family, was added as the outgroup. After the initial alignment, all reference sequences showing large insertion/deletion sites or misalignment in the conserved regions were deleted from the selection and the remaining sequences were re‐aligned. A maximum likelihood phylogenetic tree was produced using PhyML software (Guindon *et al.*, 2010) at the web server NGPhylogeny.fr (Lemoine *et al.*, 2019) using SPR moves to optimize tree topology and 10 random starting trees. Statistical branch support was calculated using 1000 bootstrap replications and the optimal substitution model was assessed by Smart Model Selection (Lefort *et al.*, 2017) to be GTR + G+I + F under the Akaike information criterion (AIC). Multiple sequence alignments and phylogenetic trees were visualized in the Jalview sequence analysis tool (Waterhouse *et al.*, 2009) and with the MEGA7 software (Kumar *et al.*, 2016) respectively. A comparable topology was achieved using ClustalOmega for sequence alignment (Madeira *et al.*, 2019).

## Conflict of Interest

The authors declare no conflict of interest.

## Author contributions

RM conducted most of the experimental work; RF performed monosaccharide analyses by LC‐MS; LS evaluated the phylogenetic analysis and provided statistical support; SF initiated the DNA‐based experiments; FA coordinated the work and wrote the paper.

## Supporting information


**Figure S1.** Hypermethylation of monosaccharides of algae N‐glycans.
**Figure S2.** Gas chromatography‐mass spectrometry (GC‐MS) analysis of the dominant N‐glycan from a ‘Jos’ group sample.
**Figure S3.** Characterization of the ‘Ori’ glyco‐group oligosaccharide with *m*/*z* = 1269.5 (os3312).
**Figure S4.** Hypermethylation analysis of mixtures of monosaccharides.
**Figure S5.** MALDI‐TOF/TOF fragment spectrum of *m*/*z* 1211.3 from two algae and the typical plant complex‐type N‐glycan MMXF^3^.
**Figure S6.** Tandem mass spectrometry (MS/MS) spectra of the major glycan of the ‘Raa’ and the ‘Now’ glyco‐group.
**Figure S7.** Monosaccharide analysis by gas chromatography‐mass spectrometry (GC‐MS) of os2221 from glyco‐groups ‘Raa’ and ‘Now’.
**Figure S8.** Monosaccharide analysis os3312 with three HexNAc residues from glyco‐group ‘Sun’.
**Figure S9.** Chromatographic behaviour of pentose‐containing N‐glycans.
**Figure S10.** Fucosylation of the Man5Gn structure from the ‘Sol’ glyco‐group.
**Figure S11.** LIFT spectrum and composition analysis for the ‘Pit’ glyco‐group.
**Figure S12.** Matrix assisted laser desorption ionization‐time of flight mass spectrometry (MALDI‐TOF MS) spectra of N‐glycans of uncategorized *Chlorella* products.
**Figure S13.** Matrix assisted laser desorption ionization‐time of flight mass spectrometry (MALDI‐TOF MS) spectra of N‐glycans of collection strains with a ‘Hel’ glycan pattern.
**Figure S14.** Matrix assisted laser desorption ionization‐time of flight mass spectrometry (MALDI‐TOF MS) spectra of N‐glycans of collection strains with a ‘Raa’ glycan pattern.

**Table S1.** Commercial products used in the present study.
**Table S2.** BLAST results for genomic ITS1–5.8S–ITS2 rRNA sequences obtained from selected *Chlorella* products.

**Data S1.** Nucleotide sequences of the ITS1–5.8S–ITS2 rRNA gene with flanking regions of 18S and 26S rDNA.

**Data S2.** MALDI‐TOF MS spectra of N‐glycans of all samples considered in this study.

## Data Availability

All data are reported within the paper or in the Supporting Information.

## References

[tpj14718-bib-0001] Ambati, R.R. , Gogisetty, D. , Aswathnarayana Gokare, R. , Ravi, S. , Bikkina, P.N. , Su, Y. and Lei, B. (2018) *Botryococcus* as an alternative source of carotenoids and its possible applications – an overview. Crit. Rev. Biotechnol. 38, 541–558. 28936877 10.1080/07388551.2017.1378997

[tpj14718-bib-0002] Anumula, K.R. (1995) Rapid quantitative determination of sialic acids in glycoproteins by high‐performance liquid chromatography with a sensitive fluorescence detection. Anal. Biochem. 230, 24–30. 8585625 10.1006/abio.1995.1432

[tpj14718-bib-0003] Blanc, G. , Duncan, G. , Agarkova, I. ***et al*** **.** (2010) The *Chlorella* variabilis NC64A genome reveals adaptation to photosymbiosis, coevolution with viruses, and cryptic sex. Plant Cell, 22, 2943–2955. 20852019 10.1105/tpc.110.076406 PMC2965543

[tpj14718-bib-0004] Bleakley, S. and Hayes, M. (2017) Algal proteins: extraction, application, and challenges concerning production. Foods, 6, 33. 10.3390/foods6050033 PMC5447909 28445408

[tpj14718-bib-0005] Bock, C. , Krienitz, L. and Pröschold, T. (2011a) Taxonomic reassessment of the genus *Chlorella* (Trebouxiophyceae) using molecular signatures (barcodes), including description of seven new species. Fottea, 11, 293–312.

[tpj14718-bib-0006] Bock, C. , Proschold, T. and Krienitz, L. (2011b) Updating the genus dictyosphaerium and description of mucidosphaerium gen. nov. (trebouxiophyceae) based on morphological and molecular data1. J. Phycol. 47, 638–652. 27021993 10.1111/j.1529-8817.2011.00989.x

[tpj14718-bib-0007] Brockhausen, I. , Hull, E. , Hindsgaul, O. , Schachter, H. , Shah, R.N. , Michnick, S.W. and Carver, J.P. (1989) Control of glycoprotein synthesis. Detection and characterization of a novel branching enzyme from hen oviduct, UDP‐N‐acetylglucosamine:GlcNAc beta 1–6 (GlcNAc beta 1–2)Man alpha‐R (GlcNAc to Man) beta‐4‐N‐acetylglucosaminyltransferase VI. J. Biol. Chem. 264, 11211–11221. 2525556

[tpj14718-bib-0008] Champenois, J. , Marfaing, H. and Pierre, R. (2015) Review of the taxonomic revision of *Chlorella* and consequences for its food uses in Europe. J. Appl. Phycol. 27, 1845–1851.

[tpj14718-bib-0009] Doughman, S.D. , Krupanidhi, S. and Sanjeevi, C.B. (2007) Omega‐3 fatty acids for nutrition and medicine: considering microalgae oil as a vegetarian source of EPA and DHA. Curr. Diabetes Rev. 3, 198–203. 18220672 10.2174/157339907781368968

[tpj14718-bib-0010] Eckardt, N.A. (2010) The *Chlorella* genome: big surprises from a small package. Plant Cell, 22, 2924. 20852020 10.1105/tpc.110.220911 PMC2965539

[tpj14718-bib-0011] Eladel, H. , Abomohra, A.E. , Battah, M. , Mohmmed, S. , Radwan, A. and Abdelrahim, H. (2019) Evaluation of *Chlorella* sorokiniana isolated from local municipal wastewater for dual application in nutrient removal and biodiesel production. Bioprocess Biosyst. Eng. 42, 425–433. 30465129 10.1007/s00449-018-2046-5

[tpj14718-bib-0012] Fucikova, K. , Pazoutova, M. and Rindi, F. (2015) Meiotic genes and sexual reproduction in the green algal class Trebouxiophyceae (Chlorophyta). J. Phycol. 51, 419–430. 26986659 10.1111/jpy.12293

[tpj14718-bib-0013] Galili, U. (2005) The alpha‐gal epitope and the anti‐Gal antibody in xenotransplantation and in cancer immunotherapy. Immunol. Cell Biol. 83, 674–686. 16266320 10.1111/j.1440-1711.2005.01366.x

[tpj14718-bib-0014] Goers, M. , Schumann, R. , Hepperle, D. and Karsten, U. (2010) Quality analysis of commercial *Chlorella* products used as dietary supplement in human nutrition. J. Phycol. 22, 265–276.

[tpj14718-bib-0015] Grass, J. , Pabst, M. , Kolarich, D. , Poltl, G. , Leonard, R. , Brecker, L. and Altmann, F. (2011) Discovery and structural characterization of fucosylated oligomannosidic N‐glycans in mushrooms. J. Biol. Chem. 286, 5977–5984. 21169363 10.1074/jbc.M110.191304 PMC3057827

[tpj14718-bib-0016] Grimsley, N. , Pequin, B. , Bachy, C. , Moreau, H. and Piganeau, G. (2010) Cryptic sex in the smallest eukaryotic marine green alga. Mol. Biol. Evol. 27, 47–54. 19734297 10.1093/molbev/msp203

[tpj14718-bib-0017] Grossmann, L. , Hinrichs, J. and Weiss, J. (2019) Solubility and aggregation behavior of protein fractions from the heterotrophically cultivated microalga *Chlorella* protothecoides. Food Res. Int. 116, 283–290. 30716947 10.1016/j.foodres.2018.08.037

[tpj14718-bib-0018] Guccione, A. , Biondi, N. , Sampietro, G. , Rodolfi, L. , Bassi, N. and Tredici, M.R. (2014) *Chlorella* for protein and biofuels: from strain selection to outdoor cultivation in a Green Wall Panel photobioreactor. Biotechnol. Biofuels, 7, 84. 24932216 10.1186/1754-6834-7-84 PMC4057815

[tpj14718-bib-0019] Guindon, S. , Dufayard, J.F. , Lefort, V. , Anisimova, M. , Hordijk, W. and Gascuel, O. (2010) New algorithms and methods to estimate maximum‐likelihood phylogenies: assessing the performance of PhyML 3.0. Syst. Biol. 59, 307–321. 20525638 10.1093/sysbio/syq010

[tpj14718-bib-0020] Hadi, S.I. , Santana, H. , Brunale, P.P. , Gomes, T.G. , Oliveira, M.D. , Matthiensen, A. , Oliveira, M.E. , Silva, F.C. and Brasil, B.S. (2016) DNA barcoding green microalgae isolated from neotropical inland waters. PLoS ONE, 11, e0149284. 26900844 10.1371/journal.pone.0149284 PMC4767179

[tpj14718-bib-0021] Helliwell, K.E. (2017) The roles of B vitamins in phytoplankton nutrition: new perspectives and prospects. New Phytol. 216, 62–68. 28656633 10.1111/nph.14669

[tpj14718-bib-0022] Huss, V.A.R. , Frank, H. , Hartmann, E.C. , Hirmer, M. , Kloboucek, A. and Seidel, B.M. (1999) Biochemical taxonomy and molecular phylogeny of the genus *chlorella* sensu lato (Chlorophyta). J. Phycol. 35, 587–598.

[tpj14718-bib-0023] Kang, H. , Salim, H. , Akter, N. , Kim, D. , Kim, J. , Bang, H. , Kim, M. , Na, J. , Hwangbo, J. and Choi, H.C. (2013) Effect of various forms of dietary *Chlorella* supplementation on growth performance, immune characteristics, and intestinal microflora population of broiler chickens. J. Appl. Poultry Res. 22, 100–108.

[tpj14718-bib-0024] Katoh, K. and Standley, D.M. (2013) MAFFT multiple sequence alignment software version 7: improvements in performance and usability. Mol. Biol. Evol. 30, 772–780. 23329690 10.1093/molbev/mst010 PMC3603318

[tpj14718-bib-0025] Kiesenhofer, D.P. and Fluch, S. (2018) The promises of microalgae‐still a long way to go. FEMS Microbiol. Lett. 365, 1–3. 10.1093/femsle/fnx257 29228181

[tpj14718-bib-0026] Kumar, S. , Stecher, G. and Tamura, K. (2016) MEGA7: Molecular Evolutionary Genetics Analysis version 7.0 for bigger datasets. Mol. Biol. Evol. 33, 1870–1874. 27004904 10.1093/molbev/msw054 PMC8210823

[tpj14718-bib-0027] Lefort, V. , Longueville, J.E. and Gascuel, O. (2017) SMS: Smart Model Selection in PhyML. Mol. Biol. Evol. 34, 2422–2424. 28472384 10.1093/molbev/msx149 PMC5850602

[tpj14718-bib-0028] Lemoine, F. , Correia, D. , Lefort, V. , Doppelt‐Azeroual, O. , Mareuil, F. , Cohen‐Boulakia, S. and Gascuel, O. (2019) NGPhylogeny.fr: new generation phylogenetic services for non‐specialists. Nucleic Acids Res. 47, W260–W265. 31028399 10.1093/nar/gkz303 PMC6602494

[tpj14718-bib-0029] Lerouge, P. , Cabanes‐Macheteau, M. , Rayon, C. , Fischette‐Laine, A.C. , Gomord, V. and Faye, L. (1998) N‐glycoprotein biosynthesis in plants: recent developments and future trends. Plant Mol. Biol. 38, 31–48. 9738959

[tpj14718-bib-0030] Levy‐Ontman, O. , Arad, S.M. , Harvey, D.J. , Parsons, T.B. , Fairbanks, A. and Tekoah, Y. (2011) Unique N‐glycan moieties of the 66‐kDa cell wall glycoprotein from the red microalga Porphyridium sp. J. Biol. Chem. 286, 21340–21352. 21515680 10.1074/jbc.M110.175042 PMC3122194

[tpj14718-bib-0031] Lucas, P.‐L. , Mathieu‐Rivet, E. , Song, P.C.T. ***et al*** (2020) Multiple xylosyltransferases heterogeneously xylosylate protein N‐linked glycans in *Chlamydomonas reinhardtii* . Plant J. in press. 10.1111/tpj.14620 31777161

[tpj14718-bib-0032] Madeira, F. , Park, Y.M. , Lee, J. ***et al*** **.** (2019) The EMBL‐EBI search and sequence analysis tools APIs in 2019. Nucleic Acids Res. 47, W636–W641. 30976793 10.1093/nar/gkz268 PMC6602479

[tpj14718-bib-0033] Markou, G. , Wang, L. , Ye, J. and Unc, A. (2018) Using agro‐industrial wastes for the cultivation of microalgae and duckweeds: contamination risks and biomass safety concerns. Biotechnol. Adv. 36, 1238–1254. 29673973 10.1016/j.biotechadv.2018.04.003 PMC7125918

[tpj14718-bib-0034] Mathieu‐Rivet, E. , Scholz, M. , Arias, C. ***et al*** **.** (2013) Exploring the N‐glycosylation pathway in *Chlamydomonas reinhardtii* unravels novel complex structures. Mol. Cell Proteomics, 12, 3160–3183. 23912651 10.1074/mcp.M113.028191 PMC3820931

[tpj14718-bib-0035] Mathieu‐Rivet, E. , Kiefer‐Meyer, M.C. , Vanier, G. , Ovide, C. , Burel, C. , Lerouge, P. and Bardor, M. (2014) Protein N‐glycosylation in eukaryotic microalgae and its impact on the production of nuclear expressed biopharmaceuticals. Front. Plant Sci. 5, 359. 25183966 10.3389/fpls.2014.00359 PMC4135232

[tpj14718-bib-0036] Mócsai, R. , Figl, R. , Troschl, C. , Strasser, R. , Svehla, E. , Windwarder, M. , Thader, A. and Altmann, F. (2018) N‐glycans of the microalga *Chlorella* vulgaris are of the oligomannosidic type but highly methylated. Sci. Rep. 9, 331. 10.1038/s41598-018-36884-1 PMC6344472 30674946

[tpj14718-bib-0037] Mócsai, R. , Blaukopf, M. , Svehla, E. , Kosma, P. and Altmann, F. (2020) The N‐glycans of *Chlorella* sorokiniana and a related strain contain arabinose but have strikingly different structures. Glycobiology, in press. 10.1093/glycob/cwaa012 32039451

[tpj14718-bib-0038] Pabst, M. , Grass, J. , Toegel, S. , Liebminger, E. , Strasser, R. and Altmann, F. (2012) Isomeric analysis of oligomannosidic N‐glycans and their dolichol‐linked precursors. Glycobiology, 22, 389–399. 22038479 10.1093/glycob/cwr138

[tpj14718-bib-0039] Panahi, Y. , Darvishi, B. , Jowzi, N. , Beiraghdar, F. and Sahebkar, A. (2016) *Chlorella vulgaris*: a multifunctional dietary supplement with diverse medicinal properties. Curr. Pharm. Des. 22, 164–173. 26561078 10.2174/1381612822666151112145226

[tpj14718-bib-0040] Schulze, S. , Urzica, E. , Reijnders, M. ***et al*** **.** (2017) Identification of methylated GnTI‐dependent N‐glycans in *Botryococcus brauni* . New Phytol. 215, 1361–1369. 28737213 10.1111/nph.14713

[tpj14718-bib-0041] Strasser, R. (2016) Plant protein glycosylation. Glycobiology, 26, 926–939. 26911286 10.1093/glycob/cww023 PMC5045529

[tpj14718-bib-0042] Sun, X.M. , Ren, L.J. , Zhao, Q.Y. , Ji, X.J. and Huang, H. (2018) Microalgae for the production of lipid and carotenoids: a review with focus on stress regulation and adaptation. Biotechnol. Biofuels, 11, 272. 30305845 10.1186/s13068-018-1275-9 PMC6171298

[tpj14718-bib-0043] Tretter, V. , Altmann, F. and Marz, L. (1991) Peptide‐N4‐(N‐acetyl‐beta‐glucosaminyl)asparagine amidase F cannot release glycans with fucose attached alpha 1–3 to the asparagine‐linked N‐acetylglucosamine residue. Eur. J. Biochem. 199, 647–652. 1868849 10.1111/j.1432-1033.1991.tb16166.x

[tpj14718-bib-0044] Vanier, G. , Lucas, P.L. , Loutelier‐Bourhis, C. ***et al*** **.** (2017) Heterologous expression of the N‐acetylglucosaminyltransferase I dictates a reinvestigation of the N‐glycosylation pathway in *Chlamydomonas reinhardtii* . Sci. Rep. 7, 10156. 28860654 10.1038/s41598-017-10698-z PMC5578997

[tpj14718-bib-0045] Varki, A. (2001) Loss of N‐glycolylneuraminic acid in humans: mechanisms, consequences, and implications for hominid evolution. Am. J. Phys. Anthropol. Suppl. 33, 54–69. 10.1002/ajpa.10018 PMC7159735 11786991

[tpj14718-bib-0046] Waterhouse, A.M. , Procter, J.B. , Martin, D.M. , Clamp, M. and Barton, G.J. (2009) Jalview Version 2 – a multiple sequence alignment editor and analysis workbench. Bioinformatics, 25, 1189–1191. 19151095 10.1093/bioinformatics/btp033 PMC2672624

[tpj14718-bib-0047] Wells, M.L. , Potin, P. , Craigie, J.S. , Raven, J.A. , Merchant, S.S. , Helliwell, K.E. , Smith, A.G. , Camire, M.E. and Brawley, S.H. (2017) Algae as nutritional and functional food sources: revisiting our understanding. J. Appl. Phycol. 29, 949–982. 28458464 10.1007/s10811-016-0974-5 PMC5387034

[tpj14718-bib-0048] Windwarder, M. , Figl, R. , Svehla, E. , Mocsai, R.T. , Farcet, J.B. , Staudacher, E. , Kosma, P. and Altmann, F. (2016) "Hypermethylation" of anthranilic acid‐labeled sugars confers the selectivity required for liquid chromatography‐mass spectrometry. Anal. Biochem. 514, 24–31. 27640150 10.1016/j.ab.2016.09.008

[tpj14718-bib-0049] Winwood, R.J. (2013) Recent developments in the commercial production of DHA and EPA rich oils from micro‐algae. OCL, 20, 1–5.

[tpj14718-bib-0050] Yusibov, V. , Kushnir, N. and Streatfield, S.J. (2016) Antibody production in plants and green algae. Annu. Rev. Plant Biol. 67, 669–701. 26905655 10.1146/annurev-arplant-043015-111812

[tpj14718-bib-0051] Zou, S. , Fei, C. , Song, J. , Bao, Y. , He, M. and Wang, C. (2016) Combining and comparing coalescent, distance and character‐based approaches for barcoding microalgaes: a test with *Chlorella*‐like species (Chlorophyta). PLoS ONE, 11, e0153833. 27092945 10.1371/journal.pone.0153833 PMC4841637

