# Supplementary material for: A first view on the unsuspected intragenus diversity of N‐glycans in Chlorella microalgae
Source: Plant J. 2020 Mar 17;103(1):184–96. doi: 10.1111/tpj.14718 (PMC7383745; doi:10.1111/tpj.14718)
Supplement: Supplementary file 1 — Figure S1. Hypermethylation of monosaccharides of algae N‐glycans. Figure S2. Gas chromatography‐mass spectrometry (GC‐MS) analysis of the dominant N‐glycan from a ‘Jos’ group sample. Figure S3. Characterization of the ‘Ori’ glyco‐group oligosaccharide with m/z = 1269.5 (os3312). Figure S4. Hypermethylation analysis of mixtures of monosaccharides. Figure S5. MALDI‐TOF/TOF fragment spectrum of m/z 1211.3 from two algae and the typical plant complex‐type N‐glycan MMXF3. Figure S6. Tandem mass spectrometry (MS/MS) spectra of the major glycan of the ‘Raa’ and the ‘Now’ glyco‐group. Figure S7. Monosaccharide analysis by gas chromatography‐mass spectrometry (GC‐MS) of os2221 from glyco‐groups ‘Raa’ and ‘Now’. Figure S8. Monosaccharide analysis os3312 with three HexNAc residues from glyco‐group ‘Sun’. Figure S9. Chromatographic behaviour of pentose‐containing N‐glycans. Figure S10. Fucosylation of the Man5Gn structure from the ‘Sol’ glyco‐group. Figure S11. LIFT spectrum and composition analysis for the ‘Pit’ glyco‐group. Figure S12. Matrix assisted laser desorption ionization‐time of flight mass spectrometry (MALDI‐TOF MS) spectra of N‐glycans of uncategorized Chlorella products. Figure S13. Matrix assisted laser desorption ionization‐time of flight mass spectrometry (MALDI‐TOF MS) spectra of N‐glycans of collection strains with a ‘Hel’ glycan pattern. Figure S14. Matrix assisted laser desorption ionization‐time of flight mass spectrometry (MALDI‐TOF MS) spectra of N‐glycans of collection strains with a ‘Raa’ glycan pattern. [file TPJ-103-184-s001.docx]

**Supporting Information for**

**A first view on the unsuspected intra-genus diversity of N-glycans in *Chlorella* microalgae**

Réka Mócsai^a^, Rudolf Figl, Leander Sützl^b^, Silvia Fluch^c^, Friedrich Altmann^a,1^

^a^ Department of Chemistry, and ^b^ Department of Food technology of the University of Natural Resources and Life Sciences, Vienna (BOKU), Vienna, Austria; ^c^ Ecoduna AG, Bruck an der Leitha, Austria

**Supporting Figures**

**Figure S1. Hypermethylation of monosaccharides of algae N-glycans.** This technique was applied to verify epimeric nature of methylated sugars. For the sample Kei C-1, the AA-labelled peak at 26.8 min (**A**) was isolated assuming it to be the methylated pentose. Upon hypermethylation, RP-LC-ESI-MS confirmed this assumption and disclosed the compound as methyl-arabinose (**B**).

**Figure S2 GC-MS analysis of the dominant N-glycan from a ‘Jos’ group sample.** The monosaccharides of the glycan with *m/z* = 1373.4 (os4221) from the ‘Jos’ group glycans were analysed as alditol acetates showing the presence of methylated galactose. Panel **A** is the MALDI-TOF MS spectrum of the HILIC fraction with highly enriched os4221. Panel **B** shows the GC-MS run of the alditol acetates derived thereof and panel **C** identifies the peak at 25.1 min as a 3-*O-*methyl hexose (-galactose by retention time). Contaminating non-sugar peaks are marked with a “c”.

**Figure S3**  **Characterization of the ‘Ori’ glyco-group oligosaccharide with *m/z* = 1269.5 (os3312)**. Panel **A** shows the MALDI-TOF/TOF fragment pattern with the prominent loss of 146.1 mass units. Panel **B** depicts the MALDI spectra of underivatized (upper spectrum) and of permethylated (bottom spectrum) glycans from a HILIC fraction rich in os3312. A methyl-pentose would have given *m/z* = 1535.76. The actually measured mass is consistent with the presence of a deoxyhexose.

**Figure S4.**  **Hypermethylation analysis of mixtures of monosaccharides**. The entire mixture of AA-labelled monosaccharides was “hyper”-methylated with deuteromethyl ioded and analyzed by reversed-phase LC-ESI-MS on an ion trap. Completely deuteromethylated sugars are found with *m/z* = 374.3, 388.3, 421.3 and 437.3 for pentoses, deoxyhexoses, hexoses and hexosmaines, respectively. Naturally methylated sugars are lighter by 3 mass units and elute a tiny bit later than the the fully deuterated sugar. For the ‘Now’, ‘Sol’, and ‘Jar’ samples the ambiguity between 3-O-methyl arabinose and 3-O-methyl-xylose could be resolved. The methylated aminosugar in ‘Sun’ could be identified as methyl-GlcNAc despite obvious problems with stochiometric release of this building block (similar to the GC-MS analysis). The ‘Ori’ sample contained fucose and also methyl-mannose – the latter due to the presence of residual methylated oligomannosidic glycans in this HILIC fraction.

**Figure S5.**  **MALDI-TOF/TOF fragment spectrum of m/z 1211.3 from two algae and the typical plant complex-type N-glycan MMXF^3^.** Fragment types are emphasized by different colours and the b-fragment indicating a non-substituted reducing GlcNAc is labelled with “-1 Gn”. Supposed y-, b-, or by-ions are shown in different colors with the respective assumed monomer compositions.

**Figure S6 MS/MS spectra of the major glycan of the ‘Raa’ and the ‘Now’ glyco-group.** The MALDI-LIFT spectrum (**A**) only shows marginal differences between *m/z* = 1049.3 peaks of the two samples with exception of the peak at 903.3. The ESI-MS spectra, albeit with another ‘Raa’ sample are very different.

**Figure S7 Monosaccharide analysis by GC-MS of os2221 from glyco-groups ‘Raa’ and ‘Now’.** GC-MS chromatograms of the glycans with identical mass from samples Raa-C-64 and Now-C-5. ‘c’ stands for non-sugar impurities.

**Figure S8 Monosaccharide analysis os3312 with three HexNAc residues from glyco-group ‘Sun’.** Panel **A** is the MALDI-TOF MS spectrum of the relevant HILIC fraction. Panel **B** shows the GC-MS chromatogram and panel **C** the fragment pattern of the peak at 31.18 min, which represents a 3- or 4-*O*-methyl-HexNAc. The 4-linkage was established by reduction with BH_4_^-^, which resulted in an unambiguous 260 mass units fragment. The epimeric nature as being 4-*O*-methyl-GlcNAc was verified by HPLC of hypermethylated monosaccharides (Figure S4).

**Figure S9 Chromatographic behaviour of pentose containing N-glycans.** Extension of Figure 4 of the main text.

**Figure S10 Fucosylation of the Man5Gn structure from the ‘Sol’ glyco-group.** A fraction of the glycans of Sol C-53 that was enriched in the MALDI peak *m/z* =1460.5 was incubated for 3 h with GDP-fucose in the absence (**A**) or presence (**B**) of recombinant human α1,6-fucosyltransferase. Incubation mixtures were analyzed by PGC-LC-ESI-MS using an ion trap mass spectrometer. Panel **C** shows the positive mode MS/MS spectrum of the presumed Man5GnF^6^ peak (*m/z* = 792.8) wherein the fragment at *m/z* = 1217.5 is particularily indicative of core fucosylation.

**Figure S11**  **LIFT spectrum and composition analysis for the ‘Pit’ glyco-group.** The upper panel shows the fragment spectra of the major N-glycans. The y-fragments inidicated the presence of terminal pentose and methyl-hexose. The GC-MS chromatogram in the lower panel identifies these residues as xylose and 3-*O*-methyl-mannose.

**Figure S12 MALDI-TOF MS spectra of N-glycans of uncategorized *Chlorella* products.**

**Figure S13. MALDI-TOF MS spectra of N-glycans of collection strains with a ‘Hel’ glycan pattern.**

**Figure S14. MALDI-TOF MS spectra of N-glycans of collection strains with a ‘Raa’ glycan pattern.**
