# Supplementary material for: A first view on the unsuspected intragenus diversity of N‐glycans in Chlorella microalgae
Source: Plant J. 2020 Mar 17;103(1):184–96. doi: 10.1111/tpj.14718 (PMC7383745; doi:10.1111/tpj.14718)
Supplement: Supplementary file 2 — Table S1. Commercial products used in the present study. Table S2. BLAST results for genomic ITS1–5.8S–ITS2 rRNA sequences obtained from selected Chlorella products. [file TPJ-103-184-s002.docx]

**Supporting Information for**

**A first view on the unsuspected intra-genus diversity of N-glycans in *Chlorella* microalgae**

Réka Mócsai^a^, Rudolf Figl, Leander Sützl^b^, Silvia Fluch^c^, Friedrich Altmann^a,1^

^a^ Department of Chemistry, and ^b^ Department of Food technology of the University of Natural Resources and Life Sciences, Vienna (BOKU), Vienna, Austria; ^c^ Ecoduna AG, Bruck an der Leitha, Austria

**Table S1. Commercial products used in the present study**

Continued on following page

**Table S1** continued

**Table S2. BLAST results for genomic ITS1-5.8S-ITS2 rRNA sequences obtained from selected *Chlorella* products.** Note that except from two matches with Scenedesmus sp. all other homologs belong to the *Chlorellaceae* family.

| Product | Closest match species | Identity (%) | NCBI nucleotide accession Nr |
| --- | --- | --- | --- |
| Kei C-1 | Chlorella sorokiniana UTEX 2805 | 97.3 | KJ676109 |
| Hel C-32 | Chlorella sorokiniana UTEX 1665 | 100.0 | KJ676113 |
| Hel C-40 | Chlorella sorokiniana UTEX 1665 | 99.9 | KJ676113 |
| Jos C-23 | Chlorella sorokiniana isolate SM12_1 | 98.2 | KM514859 |
| Jos C-24 | Chlorella sorokiniana isolate SM12_1 | 98.4 | KM514859 |
| Raa C-6 | Auxenochlorella pyrenoidosa isolate 1_2 | 100.0 | KM514847 |
| Sun C-36 cloneA | Micractinium pusillum CCAP 248/5 | 99.5 | FM205836 |
| Sun C-36 cloneB | Scenedesmus sp. MKB | 100.0 | KM873329 |
| Ori C-28 | Marasphaerium gattermannii CCAP 222/24 | 92.6 | GQ477057 |
| Ori C-46 cloneA | Dictyosphaerium sp. YN12-2 | 97.3 | MF664515 |
| Ori C-46 cloneB | Chlorella sorokiniana NIES 2173 | 93.1 | AB731602 |
| Ori C-46 cloneC | Scenedesmus rubescens CCALA 475 | 92.5 | JX513884 |
| Sol C-21 | Chlorellales sp. Isolate LBA 50 | 99.5 | KT308085 |
| Sol C-22 | Chlorellales sp. Isolate LBA 50 | 99.5 | KT308085 |
| Sol C-53 | Chlorellales sp. Isolate LBA 50 | 99.5 | KT308085 |
| Jar C-45 | Chlorella sp. IFRPD 1018 | 99.8 | AB260898 |
| Jar C-61 | Chlorella sp. IFRPD 1018 | 99.8 | AB260898 |
| Jar C-75 | Chlorella sp. IFRPD 1018 | 99.8 | AB260898 |
| Gov C-3 | Dictyosphaerium sp. CCAP 222/25 | 98.2 | GQ176862 |
| Gov C-37 | Dictyosphaerium sp. CCAP 222/25 | 98.2 | GQ176862 |
| Asp C-59 | Dictyosphaerium sp. CCAP 222/25 | 97.9 | GQ176862 |
| Pit C-17 | Dictyosphaerium sp. CCAP 222/25 | 98.2 | GQ176862 |
