# Supplementary material for: A first view on the unsuspected intragenus diversity of N‐glycans in Chlorella microalgae
Source: Plant J. 2020 Mar 17;103(1):184–96. doi: 10.1111/tpj.14718 (PMC7383745; doi:10.1111/tpj.14718)
Supplement: Supplementary file 3 — Data S1. Nucleotide sequences of the ITS1–5.8S–ITS2 rRNA gene with flanking regions of 18S and 26S rDNA. [file TPJ-103-184-s003.docx]

**Supporting Information for**

**A first view on the unsuspected intra-genus diversity of N-glycans in *Chlorella* microalgae**

Réka Mócsai^a^, Rudolf Figl, Leander Sützl^b^, Silvia Fluch^c^, Friedrich Altmann^a,1^

^a^ Department of Chemistry, and ^b^ Department of Food technology of the University of Natural Resources and Life Sciences, Vienna (BOKU), Vienna, Austria; ^c^ Ecoduna AG, Bruck an der Leitha, Austria

**Supplementary Data:**

**Nucleotide sequences of ITS1-5.8S-ITS2 rRNA gene with flanking regions of 18S and 26S rDNA**

>Kei_C1

ACACACCGCCCGTCGCTCCTACCGATTGGGTGTGCTGGTGAAGTGTTCGGATTGGCGACCGGGGGCGGTCTCCGCTCTCGGCCGCCGAGAAGTTCATTAAACCCTCCCACCTAGAGGAAGGAGAAGTCGTAACAAGGTTTCCGTAGGTGAACCTGCGGAAGGATCATTGAATCGATCGAATCCACACCGGTAACCACACGTCGCCCCTGTGGTGCATTCGCCGACCTCCGGCGTTTCACCCTGGCGTCGGCCCCTGGGCTGGGGCTCTCACGAGCCGCTTTCCAGGTCCGACGGGCGCCTCCCTTGGGCTCACCCCCTGGGGCTGGCGTCGGCCAAAACCCCTGTATCCAACCCTTTTTTTAACACACCCCAAACCACAACCAACTCTGAAGCATCTTTGGTGGCCCGGCCCCGTGCCGTCCACTCCAAACCAAAGACAACTCTCAACAACGGATATCTTGGCTCCCGTATCGATGAAGAACGCAGCGAAATGCGATACGTAGTGTGAATTGCAGAATTCCGTGAACCATCGAATCTTTGAACGCAAATTGCGCCCGAGGCTTCGGCCGAGGGCATGTCTGCCTCAGCGTCGGTTTACACCCTCGCCCTCCCCCACCCTGTGTGGTGTGGTTGGTGCGGATCTGGCCCTCCCGGCTCCGCTCTCCTTGAGCGTCCGGGTTGGCTGAAGTGGAGAGGCTTGAGCATGGACCCCGTTTGTAGGGCAATGGCTTGGTAGGTAGGCACCCCCTACGCAGCCTGCCGTTGCCCGAGGGGACTTTGCTGGAGGCCCAGCAGGAATCCGGCTGTCTTTGGCAGCCGGACTACTCACTCATTCGACCTGAGCTCAGGCAAGA

>Hel_C32

ACACACCGCCCGTCGCTCCTACCGATTGGGTGTGCTGGTGAAGTGTTCGGATTGGCGACCGGGGGCGGTCTCCGCTCTCGGCCGCCGAGAAGTTCATTAAACCCTCCCACCTAGAGGAAGGAGAAGTCGTAACAAGGTTTCCGTAGGTGAACCTGCGGAAGGATCATTGAATCGATCGAATCCACACCGGTAACCACACTGTCGCCCTCGGCGGTGCATTCTCTGGCTTCGGCTGGGTTTCACCCCGAGCGTCGGCCCCTGGGTTGGGGTTCTCACGAGCCGCTCTCCAGGTCCGGCGGGCGCCTCCCTTGGGCTCACCCCCTGGGGCTGTCGTCGGCCAAAACCCCTGTATCCAACCTTTTTTTTAACACACCCCAAACCACAACCAACTCTGAAGCATCTTTGGTGGCCCGGCCCCGTGCCGTCCACTCCAAACCAAAGACAACTCTCAACAACGGATATCTTGGCTCCCGTATCGATGAAGAACGCAGCGAAATGCGATACGTAGTGTGAATTGCAGAATTCCGTGAACCATCGAATCTTTGAACGCAAATTGCGCCCGAGGCTTCGGCCGAGGGCATGTCTGCCTCAGCGTCGGTTTACACCCTCGCCCTCCCCCCCTGTGGGGGGCGGTGCGGACCTGGCCCTCCCGGCTCCGCTCTCTCCCGAGCGTCCGGGTTGGCTGAAGCACAGAGGCTTGAGCATGGACCCCGTTTGTAGGGCAATGGCTTGGTAGGTAGGCACCCCCTACGCAGCCTGCCGTTGCCCGAGGGGACTTTGCTGGAGGCCCAGCAGGAATCCGGCCCTTCCCGGCCGGACTACTCACTCATTCGACCTGAGCTCAGGCAAGA

>Hel_C40

ACACACCGCCCGTCGCTCCTACCGATTGGGTGTGCTGGTGAAGTGTTCGGATTGGCGACCGGGGGCGGTCTCCGCTCTCGGCCGCCGAGAAGTTCATTAAACCCTCCCACCTAGAGGAAGGAGAAGTCGTAACAAGGTTTCCGTAGGTGAACCTGCGGAAGGATCATTGAATCGATCGAATCCACACCGGTAACCACACTGTCGCCCTCGGCGGTGCATTCTCTGGCTTCGGCTGGGTTTCACCCCGAGCGTCGGCCCCTGGGTTGGGGTTCTCACGAGCCGCTCTCCAGGTCCGGCGGGCGCCTCCCTTGGGCTCACCCCCTGGGGCTGTCGTCGGCCAAAACCCCTGTATCCAACCTTTTTTTTAACACACCCCAAACCACAACCAACTCTGAAGCATCTTTGGTGGCCCGGCCCCGTGCCGTCCACTCCAAACCAAAGACAACTCTCAACAACGGATATCTTGGCTCCCGTATCGATGAAGAACGCAGCGAAATGCGATACGTAGTGTGAATTGCAGAATTCCGTGAACCATCGAATCTTTGAACGCAAATTGCGCCCGAGGCTTCGGCCGAGGGCATGTCTGCCTCAGCGTCGGTTTACACCCTCGCCCTCCCCCCCTGTGGGGGGCGGTGCGGACCTGGCCCTCCCGGCTCCGCTCTCTCCCGAACGTCCGGGTTGGCTGAAGCACAGAGGCTTGAGCATGGACCCCGTTTGTAGGGCAATGGCTTGGTAGGTAGGCACCCCCTACGCAGCCTGCCGTTGCCCGAGGGGACTTTGCTGGAGGCCCAGCAGGAATCCGGCCCTTCCCGGCCGGACTACTCACTCATTCGACCTGAGCTCAGGCAAGA

>Jos_C23

ACACACCGCCCGTCGCTCCTACCGATTGGGTGTGCTGGTGAAGTGTTCGGATTGGCGACCGGGGGCGGTCTCCGCTCTCGGCCGCCGAGAAGTTCATTAAACCCTCCCACCTAGAGGAAGGAGAAGTCGTAACAAGGTTTCCGTAGGTGAACCTGCGGAAGGATCATTGAATCGATCGAATCCACACCGGTAACCAAACGTCGCCCCCCTGTGGTGCATTCTCCGGACATCCGGCGTTTCACCCTGGGCGTCGGCCCCTGGGCTGGGGCTCTCACGAGCCGCTTTCTAGGTCCGACGGGCGCCTCCCTTGGGCTCACCCCCCGGGGCTGGCGTCGGCCAAAACCCCTGTATCCAACCCTTTTTTAACACACCCCAAACCACAACCAACTCTGAAGCATCTTTGGTGGTCCGGCCTCGTGCCGTCCACTTCAAACCAAAGACAACTCTCAACAACGGATATCTTGGCTCCCGTATCGATGAAGAACGCAGCGAAATGCGATACGTAGTGTGAATTGCAGAATTCCGTGAACCATCGAATCTTTGAACGCAAATTGCGCCCGAGGCTTCGGCCGAGGGCATGTCTGCCTCAGCGTCGGTTTACACCCTCGCCCTCCCCCACCCTGTGTGGTGGGGTGTTGGTGCGGATCTGGCCCTCCCGGCTCCGCTCTGATGAGCGTCCGGGTTGGCTGAAGTGCAGAGGCTTGAGCATGGACCCCGTTTGTAGGGCAATGGCTTGGTAGGTAGGCACCCCCTACGCAGCCTGCCGTTGCCCGAGGGGACTTTGCTGGAGGCCCAGCAGGAATCCGGCTGTTTCGGCAGCCGGACTACTCACTCATCGAC

>Jos_C24

ACACACCGCCCGTCGCTCCTACCGATTGGGTGTGCTGGTGAAGTGTTCGGATTGGCGACCGGGGGCGGTCTCCGCTCTCGGCCGCCGAGAAGTTCATTAAACCCTCCCACCTAGAGGAAGGAGAAGTCGTAACAAGGTTTCCGTAGGTGAACCTGCGGAAGGATCATTGAATCGATCGAATCCACACCGGTAACCAAACGTCGCCCCCCTGTGGTGCATTCTCCGGACATCCGGCGTTTCACCCTGGGCGTCGGCCCCTGGGCTGGGGCTCTCACGAGCCGCTTTCTAGGTCCGACGGGCGCCTCCCTTGGGCTCACCCCCCGGGGCTGGCGTCGGCCAAAACCCCTGTATCCAACCCTTTTTTAACACACCCCAAACCACAACCAACTCTGAAGCATCTTTGGTGGTCCGGCCTCGTGCCGTCCACTTCAAACCAAAGACAACTCTCAACAACGGATATCTTGGCTCCCGTATCGATGAAGAACGCAGCGAAATGCGATACGTAGTGTGAATTGCAGAATTCCGTGAACCATCGAATCTTTGAACGCAAATTGCGCCCGAGGCTTCGGCCGAGGGCATGTCTGCCTCAGCGTCGGTTTACACCCTCGCCCTCCCCCACCCTGTGTGGTGGGGTGTTGGTGCGGATCTGGCCCTCCCGGCTCCGCTCTGATGAGCGTCCGGGTTGGCTGAAGTGCAGAGGCTTGAGCATGGACCCCGTTTGTAGGGCAATGGCTTGGTAGGTAGGCACCCCCTACGCAGCCTGCCGTTGCCCGAGGGGACTTTGCTGGAGGCCCAGCAGGAATCCGGCTGTTTCGGCAGCCGGACTACTCACTCATTCGACCTGAGCTCAGGCAAGA

>Raa_C6

ACACACCGCCCGTCGCTCCTACCGATTGGGTGTGCTGGTGAAGTGTTCGGATTGGCGACCGGGTGCGGTCTCCGCTCTCGGCCGCCGAGAAGTTCATTAAACCCTCCCACCTAGAGGAAGGAGAAGTCGTAACAAGGTTTCCGTAGGTGAACCTGCGGAAGGATCATTGAATCGATCGAATCCACTCTGTGAACCAAACGTCCCCCCTTGGGTGCGGGCTTCGGTCTGCCCCAAGGCGTCGGTTCCCTGGCTGGGGTCTTCGGACCGCAGTTAGGTCCGGCGGGCGCGCCCTCTGGCGTGTCGGCCCTCGTGGCTGCCGCCAGTTGGGTTCGCTGGAAATTGTATCCAACTCAACCCACCCCAAACCACAACTTATACTGAAGCAATCGGTGAGTGCACTCTGGTGCCTCGCTCTAACCAAAGACAACTCTCAACAACGGATATCTTGGCTCCCGTATCGATGAAGAACGCAGCGAAATGCGATACGTAGTGTGAATTGCAGAATTCCGTGAACCATCGAATCTTTGAACGCAAATTGCGCCCAAGGCTTCGGCCGAGGGCATGTCTGCCTCAGCGTCGGCTTACCCCCTCGCTCCCCCTCTCCTTTGGAGTGGGTGAACGGATCTGGTTTTCCCGGCTACGTGCTTCTGCACGCCCGGGTTGACTGAAGTGTAGAGGCTTGAGCATGGACCCCGTTTGTAGGGCAATGGCTTGGTAGGTAGCTTAGCTACACCGCCTGCCGTGGTCCGAGGGGACTTTGCTGGCGGCCCAGCAGGAATTCGGGTGTTGGGTTTCCCACCCCGAAAGCTTCAAACCTTCGACCTGAGCTCAGGCAAGA

>Sun_C36_A_abundant_clone

ACACACCGCCCGTCGCTCCTACCGATTGGGTGTGCTGGTGAAGTGTTCGGATTGGCATCTGGGGGCGGTCTCCGCTTCCTGACGCCGAGAAGTTCATTAAACCCTCCCACCTAGAGGAAGGAGAAGTCGTAACAAGGTTTCCGTAGGTGAACCTGCGGAAGGATCATTGAATCGATCGAATCCACTCTGGTAACCAAACGTCCCCCCCTTGGTGGCAGGGCTTGCCTTGTCCCATGGGCGCCGGTCCCCTGGCTGGGGCCTTCGGGCCGCAGTTAGGTCCGGCGGGTGTCCCTCCGATGCTGGGGCTTTTGCCCCTCTTCGGTTGGTGATGCTGGAAATTTATATTCAACTCAACCCACCCCAAACCTCGAATTAATCTGAAGCTGTCTTGTGTCACGCCTCGGCGTAGCACTCTAACCAAAGACAACTCTCAACAACGGATATCTTGGCTCCCGTATCGATGAAGAACGCAGCGAAATGCGATACGTAGTGTGAATTGCAGAATTCCGTGAACCATCGAATCTTTGAACGCAAATTGCGCCCAAGGCTTCGGCCAAGGGCATGTCTGCCTCAGCGTCGGCTTACCCCCTCACCCTCCCAATCCCTGTGATTGGGCAGAGTGGATCTGGCCCTCCCGGCTCCGTTCCAACTTGTTGGCACGCCCGGGTCGGCTGAAGTGTAGAGGCTTGAGCATGGACCCCGTTTGTAGGGCAATGGCTTGGTAGGTAGCCTCTGGTTACATCGCCTGCCGTTGTCCGAGGGGACTTTGCTGGCGGCCCAGCAGGAATTTGGTGCGTGCGGTTCTCCGTCGCCCAAATGCTTCACACCTTCGACCTGAGCTCAGGCAAGA

> Sun_C36_B_rare_clone

ACACACCGCCCGTCGCTCCTACCGATTGGGTGTGCTGGTGAAGTGTTCGGATTGGCAGCTTAGGGTGGCAACACCTCAGGTCTGCCGAGAAGTTCATTAAACCCTCCCACCTAGAGGAAGGAGAAGTCGTAACAAGGTCTCCGTAGGTGAACCTGCGGAGGGATCATTGAATTATTAAAACCACAATGTGAACCTCAACGTTCCGTGCCCTGGCTTGCCAGTGGGGCGACATGGTCAACACCAGGTCGTACTCACAGCTGGGTGGGCATTGTTGCCTACTCAGTGGCGCCTTGGCATGATCATACACCAGTGCTAACCACTGATAAAACCAAACTCTGAAGTTTGATTGCTATTCATTGGCAATCTTAACCAAAGACAACTCTCAACAACGGATATCTTGGCTCTCGCAACGATGAAGAACGCAGCGAAATGCGATACGTAGTGTGAATTGCAGAATTCCGTGAACCATCGAATCTTTGAACGCATATTGCGCTCGAGCCTTCGGGCAAGAGCATGTCTGCCTCAGCGTCGGTTTAATCCCTCACCCCTCCCTATTATGGGTGCGTTGATCATGTGATCAGCCATTGGGGTGGATCTGGCTTCCCCAATCTCACTTGTTGCGATTGGGTTGGCTGAAGCACAGAGGCTTAAGCAAGGACCCGATATGGGCTTCAACTGGATAGGTAGCAACGGCGTATGCCGACTACACGAAGTTGTTGCTTGTGGACTTTGTTAGGAGCCGAGCAGGAACATGCCTTGTGCATGCCTAAACTTTCGACCTGAGCTCAGGCAAGG

>Ori_C28

ACACACCGCCCGTCGCTCCTACCGATTGGGTGTGCTGGTGAAGTGTTCGGATTGGCAGCCCGGGGCGGTTCCCGCTCTGGTTTGCCGAGAAGTTCATTAAACCCTCCCACCTAGAGGAAGGAGAAGTCGTAACAAGGTTTCCGTAGGTGAACCTGCGGAAGGATCATTGAATCGATCGAACCCACACCGGTAACCACACAACCCCCCCTGGCGGCACGCCCCAGGGGCGCCAGTCCCCTGGCCCGGGCCACAACCCCGGTGCCCAGGTCTGGCGGGGTGTGCCCAGCCCCCGGGCTGGGCACGCCTGGTAATTCTGTCCAACCTCAACCCATCCCAAACCCCAAACCAAACTGAAGCTCGACTGGAAGGGCGGCTCTCAGCAGCCCCCCGACCACAAACCAAAGACAACTCTCAACAACGGATATCTTGGCTCCCGTATCGATGAAGAACGCAGCGAAATGCGATACGTAGTGTGAATTGCAGAATTCCGTGAACCATCGAATCTTTGAACGCAAATTGCGCCCGAGGCTCCGGCCAAGGGCATGCCTGCCTCAGCGTCGGCTCACACCCCTTGCCCCCCCCACCTGCTGGGGGGAGCAGACCTGGCACCCTCGGGCCAGCCTGGATTGGCTCTCCAGTCCAGCTGTGCCCGGGCCTGCTGAAGTGCAGAGGCTTGAGCATGGACCCCGTTTGCAGGGCAATGGCTTGGTAGGCTGGCCTTCACGGCTGAGCACCGCCTGCCGTTGCCTGAGGGGACTTTGCTGGGAGCCCAGCAGGAATTGGGGGCAGCCCTCACCGGCCCCCCAACCCTCTCACTTCGACCTGAGCTCAGGCAAGA

>Ori_C46_A

ACACACCGCCCGTCGCTCCTACCGATTGGGTGTGCTGGTGAAGTGTTCGGATTGGCAACCGGGGGCGGTCTCCGCTCCGGGTTGCTGAGAAGTTCATTAAACCCTCCCACCTAGAGGAAGGAGAAGTCGTAACAAGGTTTCCGTAGGTGAACCTGCGGAAGGATCATTGAATCGATCGAATCCACACCGGTAACCAACCTACCCCCCCTGGCCTCAACCCCCAGGGGCGCCAGTCCCCTGGCCCCGGCCCCCTGCCCCGTGCAGGGCCCGGGTGCCCAGGTCTGGCGGGGTGGCCCTCGGGCTGCCTGGTAATTGTCCAACCTCAACACACCCCAAACACCTAACCACACTGAAGCAATCGGAGCGGCGGCCTCGGCCCCCAATCCACAAACCAAAGACAACTCTCAACAACGGATATCTTGGCTCCCGTATCGATGAAGAACGCAGCGAAATGCGATACGTAGTGTGAATTGCAGAATTCCGTGAACCATCGAATCTTTGAACGCAACTTGCGCCCGCGGCTCCGGCCAAGGGCATGTCTGCCTCAGCGTCGGCACACACCCCTCGCCCCCCCCACCGGGTGGGGAGTGGACCTGGCACCCCCAGGCCTCGGCCAGCCCTCACCGGCTGTCGCTGGCCTGGGTCTGCTGAAGTGCAGAGGCTTGAGCATGGACCCCGTTTGCAGGGCAATGGCTTGGTAGGTAGGCGCCAGCCTGCACCCCGCCTGCCGTTGCCTGAGGGGACTTTGCTGGGAGCCCAGCAGGAATTGGGGCCCGCCCCGGCGGCCCCAACCCCTTCTCACTTCGACCTGAGCTCAGGCAAGA

>Ori_C46_B

ACACACCGCCCGTCGCTCCTACCGATTGGGTGTGCTGGTGAAGTGTTCGGATTGGCGACCGGGGGCGGTCTCCGCTCTCGGCCGCCGAGAAGTTCATTAAACCCTCCCACCTAGAGGAAGGAGAAGTCGTAACAAGGTTTCCGTAGGTGAACCTGCGGAAGGATCATTGAATCGATCGAATCCACACCGGTAACCACACTGTCGCCCTGGGTCGGGTGCGCACCTCTGCGTGCTGCCCGGCCCAGCGCCGGCCCCTGGGCTGGGGCTCTCACGAGCCGCTTCTCAGGTCCGGCGGGCGTCTCCCTTGGGCTCACCCCCCGGGGCTGCCGTCGGCCAAAACCCCTGTATCCAACCCCCTTTTTTAACACACCCCAAACCACAACCAACTCTGAAGCATCTTTGGTGGCCCGGCCCCGTGCCGTCCACTCCAAACCAAAGACAACTCTCAACAACGGATATCTTGGCTCCCGTATCGATGAGGAACGCAGCGAAATGCGATACGTAGTGTGAATTGCAGAATTCCGTGAACCATCGAATCTTTGAACGCAAATTGCGCCCGAGGCTTCGGCCGAGGGCATGCCTGCCTCAGCGTCGGTTTACACCCTCGCCCTCCCCCACCGCTTGGCTGGGGTGCTGGTGCGGATCTGGCCCTCCCGGCTCCGGCCCTGCCTTGTGCAGGGGCGCCCGGGTTGGCTGAAGCCCAGAGGCTTGAGCATGGACCCCGTTTGCAGGGCAATGGCTTGGTAGGTAGGCACCCCCTACGCAGCCTGCCGTTGCCCGAGGGGTCTTTGCTGGAGGCCCAGCAGGAATTCGGCCCTCACCGGCCGAACCACTCACTCATTCGACCTGAGCTCAGGCAAGA

>Ori_C46_C

ACACACCGCCCGTCGCTCCTACCGATTGGGTGTGCTGGTGAAGTGTTCGGATTGGCAGCTTAGGGTGGCAACACCTCAGGTCTGCCGAGAAGTTCATTAAACCCTCCCACCTAGAGGAAGGAGAAGTCGTAACAAGGTCTCCGTAGGTGAACCTGCGGAGGGATCATTGAATTATTAAAACCACAATGTGAACCTCAACGTTCCGTGCCCTGGCTTGCCAGTGGGGCGACATGGTCAACACCAGGTCGTACTCACAGCTGGGTGGGCATTGTTGCCTACTCAGTGGCGCCTTGGCATGATCATACACCAGTGCTAACCACTGATAAAACCAAACTCTGAAGTTTGATTGCTATTCATTGGCAATCTTAACCAAAGACAACTCTCAACAACGGATATCTTGGCTCCCGTATCGATGAAGAACGCAGCGAAATGCGATACGTAGTGTGAATTGCAGAATTCCGTGAACCATCGAATCTTTGAACGCATATTGCGCTCGAGCCTTCGGGCAAGAGCATGTCTGCCTCAGCGTCGGTTTACACCCTCACCCCTCCCTTTCTTGGGTGTGTTGATCTTTGATCAACCATTGGGGTGGATCTGGCTTCCCCAATCTGCCTTGTAGCGGATTGGGTTGGCTGAAGCACAGAGGCTTAAGCAAGGACCCGATATGGGCTTCAACTGGATAGGTAGCAACGGCTTGTGCCGACTACACGAAGTTGTTGCCTGTGGACTTTGCTAGAGGCCAAGCAGGAACATGCTTATGCATGCCTAAACTTTCGACCTGAGCTCAGGCAAGG

>Sol_C21

ACACACCGCCCGTCGCTCCTACCGATTGGGTGTGCTGGTGAAGTGTTCGGATTGGCGACCGGGGGCGGTCTCCGCTCTCGGCCGCCGAGAAGTTCATTAAACCCTCCCACCTAGAGGAAGGAGAAGTCGTAACAAGGTTTCCGTAGGTGAACCTGCGGAAGGATCATTGAATCGATCGAATCCACACCGGTAACCACACGTCGCCCTGTGCGGTGCTGCACTCAGCCGAGTGCACTCTGCGCAGCGTCGGCCCCTGGGCTGGGGCTCTCACGAGCCGCTTTCCAGGTCCGACGGGCGCCTCCCTTGGGCTCACCCCCCGGGGCTGTCGTCGGCCAAAACCCCTGTATCCAACCCCTTTTTTTAACACACCCCAAACCACAACCAACTCTGAAGCATCTTTGGTGGCCCGGCCCCGTGCCGTCCACTCCAAACCAAAGACAACTCTCAACAACGGATATCTTGGCTCCCGTATCGATGAAGAACGCAGCGAAATGCGATACGTAGTGTGAATTGCAGAATTCCGTGAACCATCGAATCTTTGAACGCAAATTGCGCCCGAGGCTTCGGCCGAGGGCATGTCTGCCTCAGCGTCGGTTTACACCCTCGCCCTCCCCCACCCTGTGCGGTGGGGTGCAGGTGCGGACCTGGCCCTCCCGGCTCCGCCCCGCTGTTCTTCGAGCAGCGGTGGCGCCCGGGTTGGCTGAAGCACAGAGGCTTGAGCATGGACCCCGTTTGTAGGGCAATGGCTTGGTAGGTAGGCACCCCCTACGCAGCCTGCCGTTGCCCGAGGGGACTTTGCTGGAGGCCCCGCAGGAATCCGGCCCGCCTTTGCGGGCGGCCGGAGCACTCACTCATTCGACCTGAGCTCAGGCAAGA

>Sol_C22

ACACACCGCCCGTCGCTCCTACCGATTGGGTGTGCTGGTGAAGTGTTCGGATTGGCGACCGGGGGCGGTCTCCGCTCTCGGCCGCCGAGAAGTTCATTAAACCCTCCCACCTAGAGGAAGGAGAAGTCGTAACAAGGTTTCCGTAGGTGAACCTGCGGAAGGATCATTGAATCGATCGAATCCACACCGGTAACCACACGTCGCCCTGTGCGGTGCTGCACTCAGCCGAGTGCACTCTGCGCAGCGTCGGCCCCTGGGCTGGGGCTCTCACGAGCCGCTTTCCAGGTCCGACGGGCGCCTCCCTTGGGCTCACCCCCCGGGGCTGTCGTCGGCCAAAACCCCTGTATCCAACCCCTTTTTTTAACACACCCCAAACCACAACCAACTCTGAAGCATCTTTGGTGGCCCGGCCCCGTGCCGTCCACTCCAAACCAAAGACAACTCTCAACAACGGATATCTTGGCTCCCGTATCGATGAAGAACGCAGCGAAATGCGATACGTAGTGTGAATTGCAGAATTCCGTGAACCATCGAATCTTTGAACGCAAATTGCGCCCGAGGCTTCGGCCGAGGGCATGTCTGCCTCAGCGTCGGTTTACACCCTCGCCCTCCCCCACCCTGTGCGGTGGGGTGCAGGTGCGGACCTGGCCCTCCCGGCTCCGCCCCGCTGTTCTTCGAGCAGCGGTGGCGCCCGGGTTGGCTGAAGCACAGAGGCTTGAGCATGGACCCCGTTTGTAGGGCAATGGCTTGGTAGGTAGGCACCCCCTACGCAGCCTGCCGTTGCCCGAGGGGACTTTGCTGGAGGCCCCGCAGGAATCCGGCCCGCCTTTGCGGGCGGCCGGAGCACTCACTCATTCGACCTGAGCTCAGGCAAGA

>Sol_C53

ACACACCGCCCGTCGCTCCTACCGATTGGGTGTGCTGGTGAAGTGTTCGGATTGGCGACCGGGGGCGGTCTCCGCTCTCGGCCGCCGAGAAGTTCATTAAACCCTCCCACCTAGAGGAAGGAGAAGTCGTAACAAGGTTTCCGTAGGTGAACCTGCGGAAGGATCATTGAATCGATCGAATCCACACCGGTAACCACACGTCGCCCTGTGCGGTGCTGCACTCAGCCGAGTGCACTCTGCGCAGCGTCGGCCCCTGGGCTGGGGCTCTCACGAGCCGCTTTCCAGGTCCGACGGGCGCCTCCCTTGGGCTCACCCCCCGGGGCTGTCGTCGGCCAAAACCCCTGTATCCAACCCCTTTTTTTAACACACCCCAAACCACAACCAACTCTGAAGCATCTTTGGTGGCCCGGCCCCGTGCCGTCCACTCCAAACCAAAGACAACTCTCAACAACGGATATCTTGGCTCCCGTATCGATGAAGAACGCAGCGAAATGCGATACGTAGTGTGAATTGCAGAATTCCGTGAACCATCGAATCTTTGAACGCAAATTGCGCCCGAGGCTTCGGCCGAGGGCATGTCTGCCTCAGCGTCGGTTTACACCCTCGCCCTCCCCCACCCTGTGCGGTGGGGTGCAGGTGCGGACCTGGCCCTCCCGGCTCCGCCCCGCTGTTCTTCGAGCAGCGGTGGCGCCCGGGTTGGCTGAAGCACAGAGGCTTGAGCATGGACCCCGTTTGTAGGGCAATGGCTTGGTAGGTAGGCACCCCCTACGCAGCCTGCCGTTGCCCGAGGGGACTTTGCTGGAGGCCCCGCAGGAATCCGGCCCGCCTTTGCGGGCGGCCGGAGCACTCACTCATTCGACCTGAGCTCAGGCAAGA

>Jar_C45

ACACACCGCCCGTCGCTCCTACCGATTGGGTGTGCTGGTGAAGTGTTCGGATTGGCGACCGGGGGCGGTCTCCGCTCTCGGCCGCCGAGAAGTTCATTAAACCCTCCCACCTAGAGGAAGGAGAAGTCGTAACAAGGTTTCCGTAGGTGAACCTGCGGAAGGATCATTGAATCGATCGAATCCACACCGGTAACCAAACGTCGCCCCCCTGTGGTGCATTCGCCGGACCCCCGGCGTTTCACCCTGGGCGTCGGCCCCTGGGCTGGGGCTCTCACGAGCCGCTTCTCAGGTCCGACGGGCGCCTCCCTTGGGCTCACCCCCCGGGGCTGGCGTCGGCCAAAACCCCTGTATCCAACCCTTTTTTAACACACCCCAAACCACAACTCACTCTGAAGCATCTTTGGTGGTCGTGCCTCGTGCCCTTCCACTCCAAACCAAAGACAACTCTCAACAACGGATATCTTGGCTCCCGTATCGATGAAGAACGCAGCGAAATGCGATACGTAGTGTGAATTGCAGAATTCCGTGAACCATCGAATCTTTGAACGCAAATTGCGCCCGAGGCTTCGGCCGAGGGCATGTCTGCCTCAGCGTCGGTTTACACCCTCGCCCTCCCCCACCCTGCGTGGTGGGGTGCTGGTGCGGATCTGGCCCTCCCGGCTCCCCTCTCCCTCCCAGGCGAGGCTCCGGGTTGGCTGAAGCACAGAGGCTTGAGCATGGACCCCGTTTGTAGGGCAATGGCTTGGTAGGTAGGCACCCCCTACGCAGCCTGCCGTTGCCCGAGGGGACTTTGCTGGAGGCCCAGCAGGAATCCGGTCGGTCCCTGTGGCCGCCGGACCACTCACTCATCGACCT

>Jar_C61

ACACACCGCCCGTCGCTCCTACCGATTGGGTGTGCTGGTGAAGTGTTCGGATTGGCGACCGGGGGCGGTCTCCGCTCTCGGCCGCCGAGAAGTTCATTAAACCCTCCCACCTAGAGGAAGGAGAAGTCGTAACAAGGTTTCCGTAGGTGAACCTGCGGAAGGATCATTGAATCGATCGAATCCACACCGGTAACCAAACGTCGCCCCCCTGTGGTGCATTCGCCGGACCCCCGGCGTTTCACCCTGGGCGTCGGCCCCTGGGCTGGGGCTCTCACGAGCCGCTTCTCAGGTCCGACGGGCGCCTCCCTTGGGCTCACCCCCCGGGGCTGGCGTCGGCCAAAACCCCTGTATCCAACCCTTTTTTAACACACCCCAAACCACAACTCACTCTGAAGCATCTTTGGTGGTCGTGCCTCGTGCCCTTCCACTCCAAACCAAAGACAACTCTCAACAACGGATATCTTGGCTCCCGTATCGATGAAGAACGCAGCGAAATGCGATACGTAGTGTGAATTGCAGAATTCCGTGAACCATCGAATCTTTGAACGCAAATTGCGCCCGAGGCTTCGGCCGAGGGCATGTCTGCCTCAGCGTCGGTTTACACCCTCGCCCTCCCCCACCCTGCGTGGTGGGGTGCTGGTGCGGATCTGGCCCTCCCGGCTCCCCTCTCCCTCCCAGGCGAGGCTCCGGGTTGGCTGAAGCACAGAGGCTTGAGCATGGACCCCGTTTGTAGGGCAATGGCTTGGTAGGTAGGCACCCCCTACGCAGCCTGCCGTTGCCCGAGGGGACTTTGCTGGAGGCCCAGCAGGAATCCGGTCGGTCCCTGTGGCCGCCGGACCACTCACTCATTCGACCTGAGCTCA

>Jar_C75

ACACACCGCCCGTCGCTCCTACCGATTGGGTGTGCTGGTGAAGTGTTCGGATTGGCGACCGGGGGCGGTCTCCGCTCTCGGCCGCCGAGAAGTTCATTAAACCCTCCCACCTAGAGGAAGGAGAAGTCGTAACAAGGTTTCCGTAGGTGAACCTGCGGAAGGATCATTGAATCGATCGAATCCACACCGGTAACCAAACGTCGCCCCCCTGTGGTGCATTCGCCGGACCCCCGGCGTTTCACCCTGGGCGTCGGCCCCTGGGCTGGGGCTCTCACGAGCCGCTTCTCAGGTCCGACGGGCGCCTCCCTTGGGCTCACCCCCCGGGGCTGGCGTCGGCCAAAACCCCTGTATCCAACCCTTTTTTAACACACCCCAAACCACAACTCACTCTGAAGCATCTTTGGTGGTCGTGCCTCGTGCCCTTCCACTCCAAACCAAAGACAACTCTCAACAACGGATATCTTGGCTCCCGTATCGATGAAGAACGCAGCGAAATGCGATACGTAGTGTGAATTGCAGAATTCCGTGAACCATCGAATCTTTGAACGCAAATTGCGCCCGAGGCTTCGGCCGAGGGCATGTCTGCCTCAGCGTCGGTTTACACCCTCGCCCTCCCCCACCCTGCGTGGTGGGGTGCTGGTGCGGATCTGGCCCTCCCGGCTCCCCTCTCCCTCCCAGGCGAGGCTCCGGGTTGGCTGAAGCACAGAGGCTTGAGCATGGACCCCGTTTGTAGGGCAATGGCTTGGTAGGTAGGCACCCCCTACGCAGCCTGCCGTTGCCCGAGGGGACTTTGCTGGAGGCCCAGCAGGAATCCGGTCGGTCCCTGTGGCCGCCGGACCACTCACTCATTCGACCTGAGCTCAG

>Gov_C35

ACACACCGCCCGTCGCTCCTACCGATTGGGTGTGCTGGTGAAGTGTTCGGATTGGCGACCCGGGGCGGTTTCCGCCCTGGGCTGCCGAGAAGTTCATTAAACCCTCCCACCTAGAGGAAGGAGAAGTCGTAACAAGGTTTCCGTAGGTGAACCTGCGGAAGGATCATTGAATCGATCGAATCCACACCGGTAACCATCCTACCCCCCCTGGCCTAACACCCCAGGGGCGCCAGTCCCCTGGCCCGGGCCCGACCCGGTGCCCAGGTCTGGCGGGGTGGGGGCCCGTCCCCCGCCTGGTAATTTGTCCAACCTTAACACACCCCAAACGTCAAAACCAAACTGAAGCAACTGGACTGGGCGGCCCCAGCGCCCCCCATCCGCCAAACCAAAGACAACTCTCAACAACGGATATCTTGGCTCCCGTATCGATGAAGAACGCAGCGAAATGCGATACGTAGTGTGAATTGCAGAATTCCGTGAACCATCGAATCTTTGAACGCAAATTGCGCCCGCGGCTCCGGCCAAGGGCATGCCTGCCTCAGCGTCGGCTTTCACCCCCTCGCCCCCAATACATTTGGGAGCGGACCTGGCACCCTCGGGGGCCCGGCCTTTTCCAAAGGCCGCCGCCCCCGGGCCTGCTGAAGTGCAGTGGCTTGAGCATGGACCCCGTTTGCAGGGCAATGGCTTGGTAGGTAGGCCCCGGCCTGCACCCCGCCTGCCGTTGCCTGAGGGGACTTTGCTGGGAGCCTAGCAGGAATTGGGAGCCCCAGCCCTGGCGCTGGGCCCCAACCCCCCCCATATTTCGACCTGAGCTCAGGCAAGG

>Gov_C37

ACACACCGCCCGTCGCTCCTACCGATTGGGTGTGCTGGTGAAGTGTTCGGATTGGCGACCCGGGGCGGTTTCCGCCCTGGGCTGCCGAGAAGTTCATTAAACCCTCCCACCTAGAGGAAGGAGAAGTCGTAACAAGGTTTCCGTAGGTGAACCTGCGGAAGGATCATTGAATCGATCGAATCCACACCGGTAACCATCCTACCCCCCCTGGCCTAACACCCCAGGGGCGCCAGTCCCCTGGCCCGGGCCCGACCCGGTGCCCAGGTCTGGCGGGGTGGGGGCCCGTCCCCCGCCTGGTAATTTGTCCAACCTTAACACACCCCAAACGTCAAAACCAAACTGAAGCAACTGGACTGGGCGGCCCCAGCGCCCCCCATCCGCCAAACCAAAGACAACTCTCAACAACGGATATCTTGGCTCCCGTATCGATGAAGAACGCAGCGAAATGCGATACGTAGTGTGAATTGCAGAATTCCGTGAACCATCGAATCTTTGAACGCAAATTGCGCCCGCGGCTCCGGCCAAGGGCATGCCTGCCTCAGCGTCGGCTTTCACCCCCTCGCCCCCAATACATTTGGGAGCGGACCTGGCACCCTCGGGGGCCCGGCCTTTTCCAAAGGCCGCCGCCCCCGGGCCTGCTGAAGTGCAGTGGCTTGAGCATGGACCCCGTTTGCAGGGCAATGGCTTGGTAGGTAGGCCCCGGCCTGCACCCCGCCTGCCGTTGCCTGAGGGGACTTTGCTGGGAGCCTAGCAGGAATTGGGAGCCCCAGCCCTGGCGCTGGGCCCCAACCCCCCCCATATTTCGACCTGAGCTCAGGCAAGG

>Asp_C59

ACACACCGCCCGTCGCTCCTACCGATTGGGTGTGCTGGTGAAGTGTTCGGATTGGCGACCCGGGGCGGTTTCCGCCCTGGGCTGCCGAGAAGTTCATTAAACCCTCCCACCTAGAGGAAGGAGAAGTCGTAACAAGGTTTCCGTAGGTGAACCTGCGGAAGGATCATTGAATCGATCGAATCCACACCGGTAACCATCCTACCCCCCCTGGCCTAACACCCCAGGGGCGCCAGTCCCCTGGCCCGGGCCCGACCCGGTGCCCAGGTCTGGCGGGGTGGGGGCCCGTCCCCCGCCTGGTAATTTGTCCAAGCTTAACACACCCCAAACGTCAAAACCAAACTGAAGCAACTGGACTGGGCGGCCCCAGCGCCCCCCATCCGCCAAACCAAAGACAACTCTCAACAACGGATATCTTGGCTCCCGTATCGATGAAGAACGCAGCGAAATGCGATACGTAGTGTGAATTGCAGAATTCCGTGAACCATCGAATCTTTGAACGCAAATTGCGCCCGCGGCTCCGGCCAAGGGCATGCCTGCCTCAGCGTCGGCTTTCACCCCCTCGCCCCCAATACATTTGGGAGCGGACCTGGCACCCTCGGGGGCCCGGCCTTTTCCAAAGGCCGCCGCCCCCGGGCCTGCTGAAGTGCAGTGGCTTGAGCATGGACCCCGTTTGCAGGGCAATGGCTTGCTAGGTAGGCCCCGGCCTGCACCCCGCCTGCCGTTGCCTGAGGGGACTTTGCTGGGAGCCTAGCAGGAATTGGGAGCCCCAGCCCTGGCGCTGGGCCCCAACCCCCCCCATATTTCGACCTGAGCTCAGGCAAGG

>Pit_C17

ACACACCGCCCGTCGCTCCTACCGATTGGGTGTGCTGGTGAAGTGTTCGGATTGGCGACCCGGGGCGGTTTCCGCCCTGGGCTGCCGAGAAGTTCATTAAACCCTCCCACCTAGAGGAAGGAGAAGTCGTAACAAGGTTTCCGTAGGTGAACCTGCGGAAGGATCATTGAATCGATCGAATCCACACCGGTAACCATCCTACCCCCCCTGGCCTAACACCCCAGGGGCGCCAGTCCCCTGGCCCGGGCCCGACCCGGTGCCCAGGTCTGGCGGGGTGGGGGCCCGTCCCCCGCCTGGTAATTTGTCCAACCTTAACACACCCCAAACGTCAAAACCAAACTGAAGCAACTGGACTGGGCGGCCCCAGCGCCCCCCATCCGCCAAACCAAAGACAACTCTCAACAACGGATATCTTGGCTCCCGTATCGATGAAGAACGCAGCGAAATGCGATACGTAGTGTGAATTGCAGAATTCCGTGAACCATCGAATCTTTGAACGCAAATTGCGCCCGCGGCTCCGGCCAAGGGCATGCCTGCCTCAGCGTCGGCTTTCACCCCCTCGCCCCCAATACATTTGGGAGCGGACCTGGCACCCTCGGGGGCCCGGCCTTTTCCAAAGGCCGCCGCCCCCGGGCCTGCTGAAGTGCAGTGGCTTGAGCATGGACCCCGTTTGCAGGGCAATGGCTTGGTAGGTAGGCCCCGGCCTGCACCCCGCCTGCCGTTGCCTGAGGGGACTTTGCTGGGAGCCTAGCAGGAATTGGGAGCCCCAGCCCTGGCGCTGGGCCCCAACCCCCCCCATATTTCGACCTGAGCTCAGGCAAGG

>SAG2118k_Chlorella_sorokiniana_owndata

ACACACCGCCCGTCGCTCCTACCGATTGGGTGTGCTGGTGAAGTGTTCGGATTGGCGACCGGGGGCGGTCTCCGCTCTCGGCCGCCGAGAAGTTCATTAAACCCTCCCACCTAGAGGAAGGAGAAGTCGTAACAAGGTTTCCGTAGGTGAACCTGCGGAAGGATCATTGAATCGATCGAATCCACACCGGTAACCACACTGTCGCCCTCGGCGGTGCATTCTCTGGCTTCGGCTGGGTTTCACCCCGAGCGTCGGCCCCTGGGTTGGGGTTCTCACGAGCCGCTCTCCAGGTCCGGCGGGCGCCTCCCTTGGGCTCACCCCCTGGGGCTGTCGTCGGCCAAAACCCCTGTATCCAACCTTTTTTTTAACACACCCCAAACCACAACCAACTCTGAAGCATCTTTGGTGGCCCGGCCCCGTGCCGTCCACTCCAAACCAAAGACAACTCTCAACAACGGATATCTTGGCTCCCGTATCGATGAAGAACGCAGCGAAATGCGATACGTAGTGTGAATTGCAGAATTCCGTGAACCATCGAATCTTTGAACGCAAATTGCGCCCGAGGCTTCGGCCGAGGGCATGTCTGCCTCAGCGTCGGTTTACACCCTCGCCCTCCCCCCCTGTGGGGGGCGGTGCGGACCTGGCCCTCCCGGCTCCGCTCTCTCCCGAGCGTCCGGGTTGGCTGAAGCACAGAGGCTTGAGCATGGACCCCGTTTGTAGGGCAATGGCTTGGTAGGTAGGCACCCCCTACGCAGCCTGCCGTTGCCCGAGGGGACTTTGCTGGAGGCCCAGCAGGAATCCGGCCCTTCCCGGCCGGACTACTCACTCATTCGACCTGAGCTCAGGCAAGA

>SAG211_34_owndata_GenBank_ MN194596

ACACACCGCCCGTCGCTCCTACCGATTGGGTGTGCTGGTGAAGTGTTCGGATTGGCGACCGGGTGCGGTCTCCGCTCTCGGCCGCCGAGAAGTTCATTAAACCCTCCCACCTAGAGGAAGGAGAAGTCGTAACAAGGTTTCCGTAGGTGAACCTGCGGAAGGATCATTGAATCGATCGAATCCACTCTGTGAACCAAACGTCCCCCCTTGGGTGCGGGCTTCGGTCTGCCCCAAGGCGTCGGTTCCCTGGCTGGGGTCTTCGGACCGCAGTTAGGTCCGGCGGGCGCGCCCTCTGGCGTGTCGGCCCTCGTGGCTGCCGCCAGTTGGGTTCGCTGGAAATTGTATCCAACTCAACCCACCCCAAACCACAACTTATACTGAAGCAATCGGTGAGTGCACTCTGGTGCCTCGCTCTAACCAAAGACAACTCTCAACAACGGATATCTTGGCTCCCGTATCGATGAAGAACGCAGCGAAATGCGATACGTAGTGTGAATTGCAGAATTCCGTGAACCATCGAATCTTTGAACGCAAATTGCGCCCAAGGCTTCGGCCGAGGGCATGTCTGCCTCAGCGTCGGCTTACCCCCTCGCTCCCCCTCTCCTTTGGAGTGGGTGAACGGATCTGGTTTTCCCGGCTACGTGCTTCTGCACGCCCGGGTTGACTGAAGTGTAGAGGCTTGAGCATGGACCCCGTTTGTAGGGCAATGGCTTGGTAGGTAGCTTAGCTACACCGCCTGCCGTGGTCCGAGGGGACTTTGCTGGCGGCCCAGCAGGAATTCGGGTGTTGGGTTTCCCACCCCGAAAGCTTCAAACCTTCGACCTGAGCTCAGGCAAGA
