# Supplementary material for: A first view on the unsuspected intragenus diversity of N‐glycans in Chlorella microalgae
Source: Plant J. 2020 Mar 17;103(1):184–96. doi: 10.1111/tpj.14718 (PMC7383745; doi:10.1111/tpj.14718)
Supplement: Supplementary file 4 — Data S2. MALDI‐TOF MS spectra of N‐glycans of all samples considered in this study. [file TPJ-103-184-s004.pdf]

Kei

Kei C-1

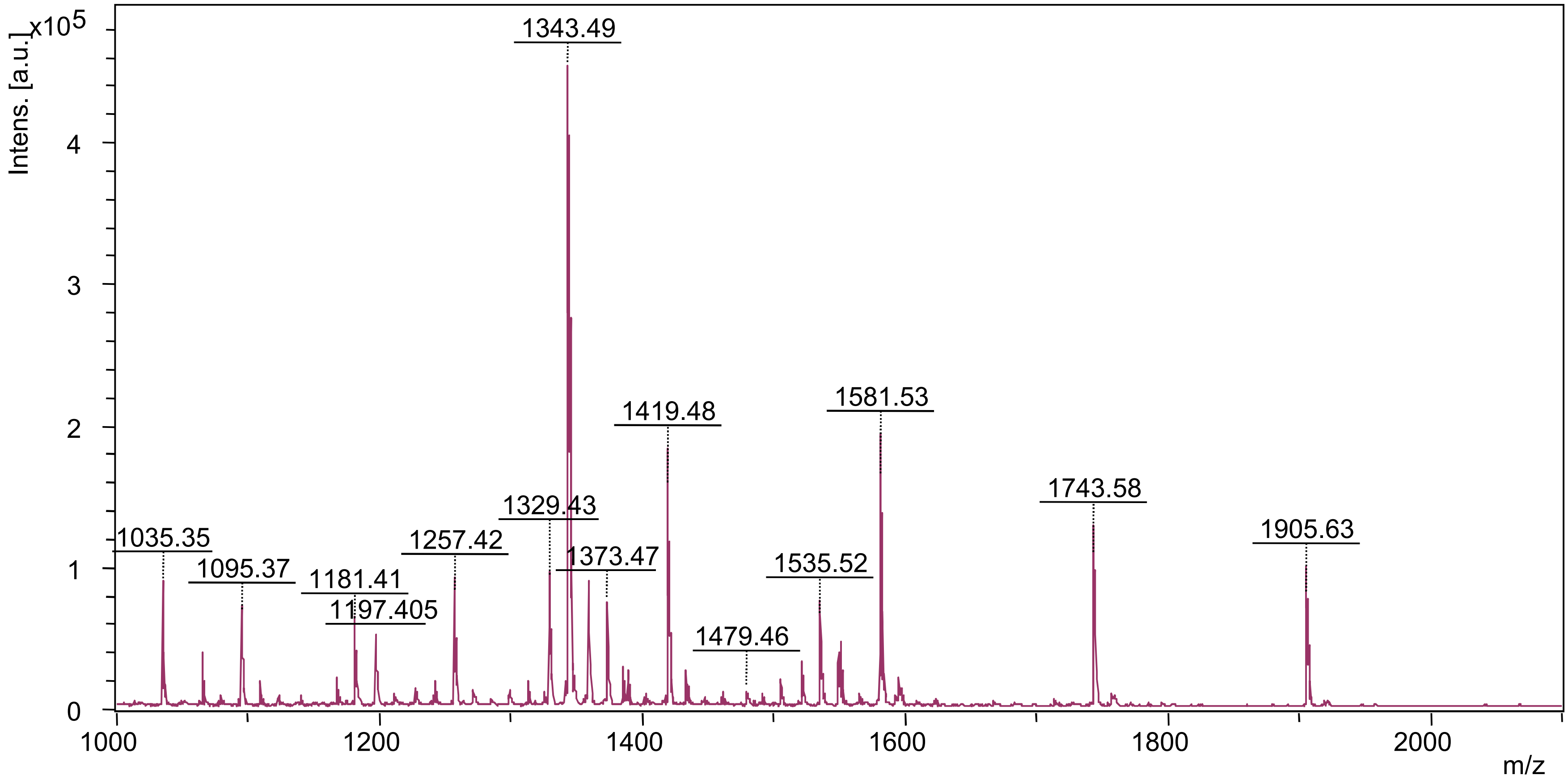

RM20170328_Chpyr_Keimling_glycans

Kei

Kei C-54

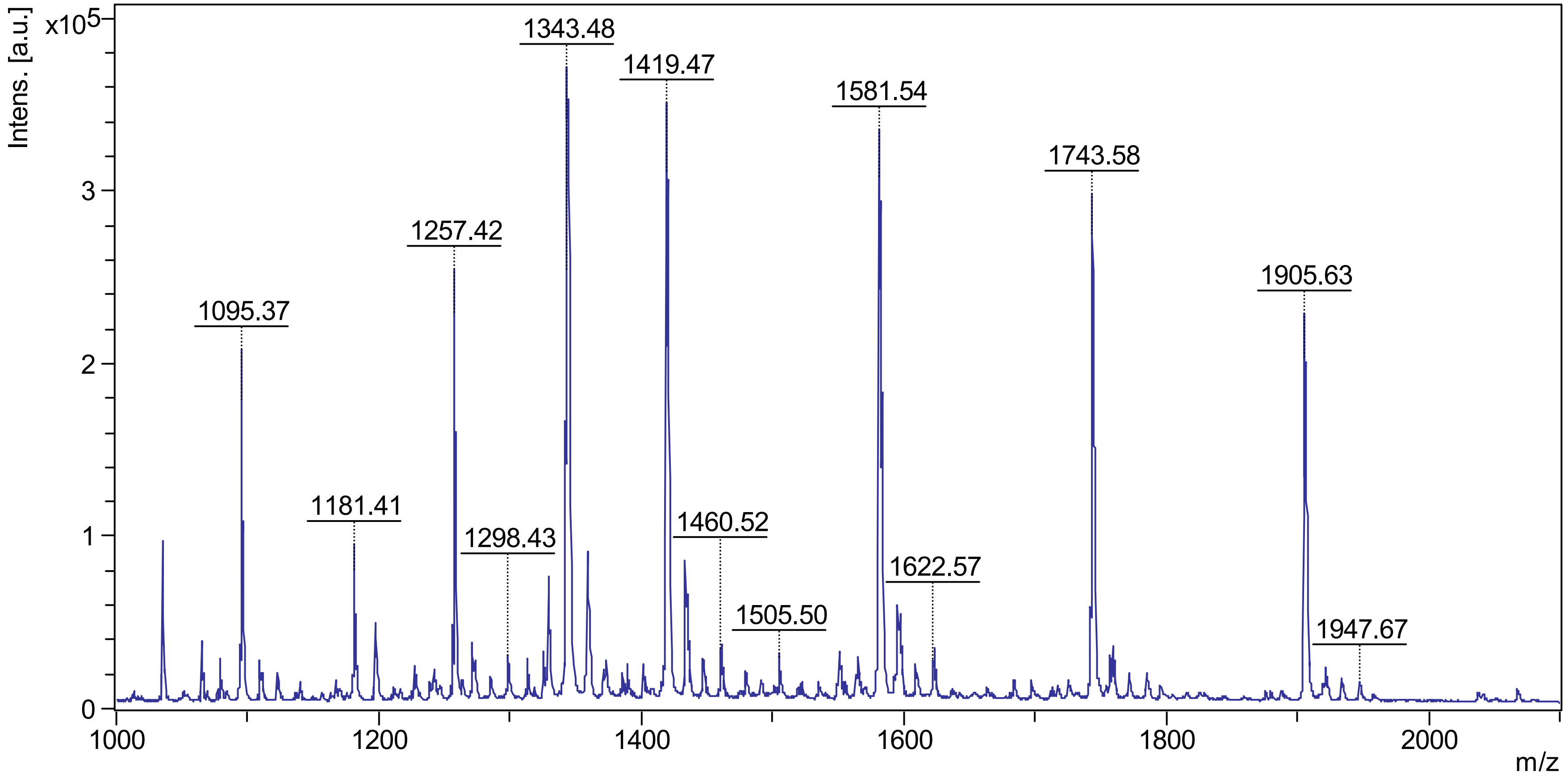

Sanatur von Alge 34

Hel

Hel C-19

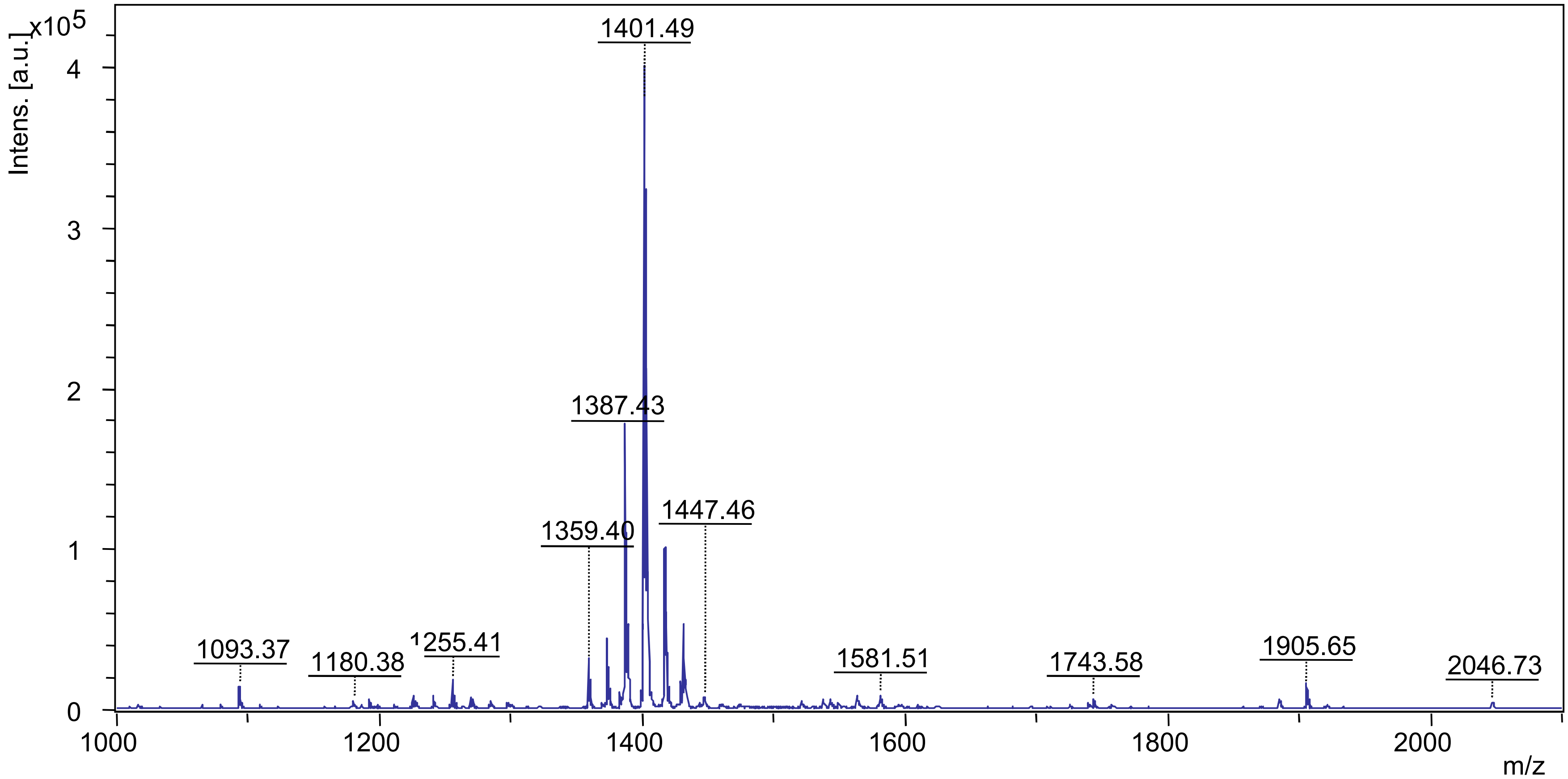

20180104_RF_RM_Ecoduna-Soro D1

Hel

Hel C-32

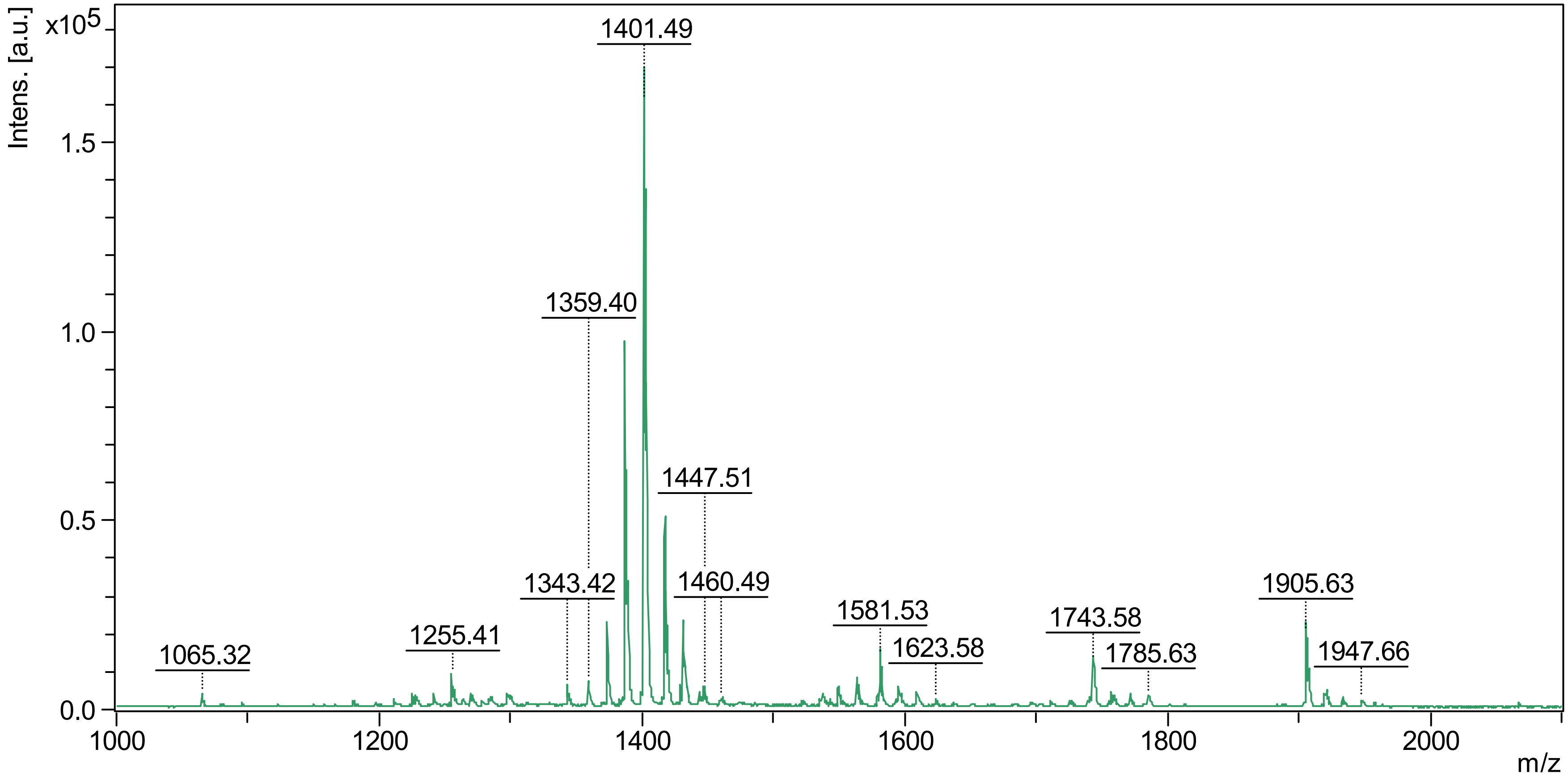

Algomed 1zu25

Hel

Hel C-33

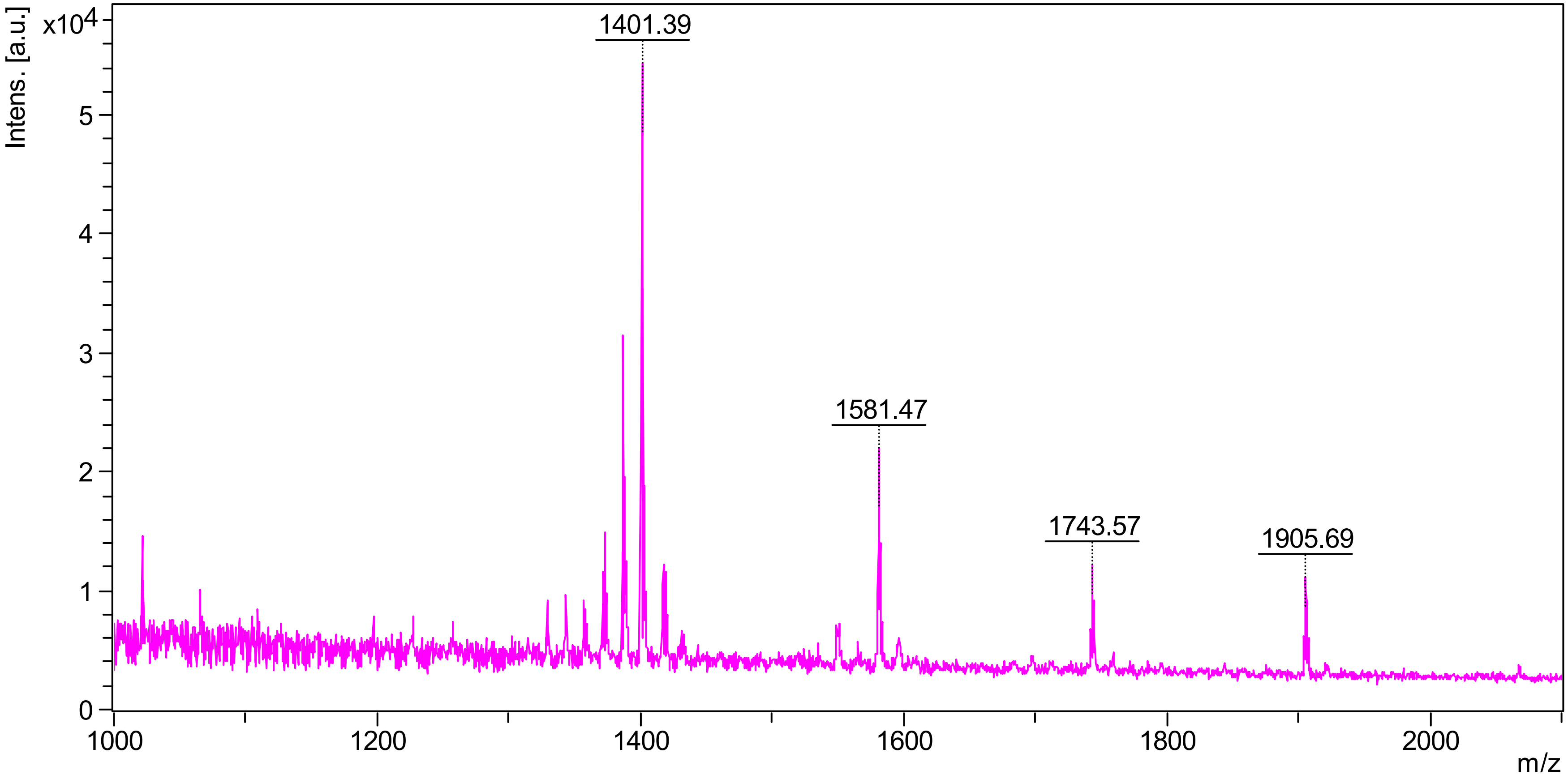

Neue Keimling Sep25_1zu5

Hel

Hel C-40

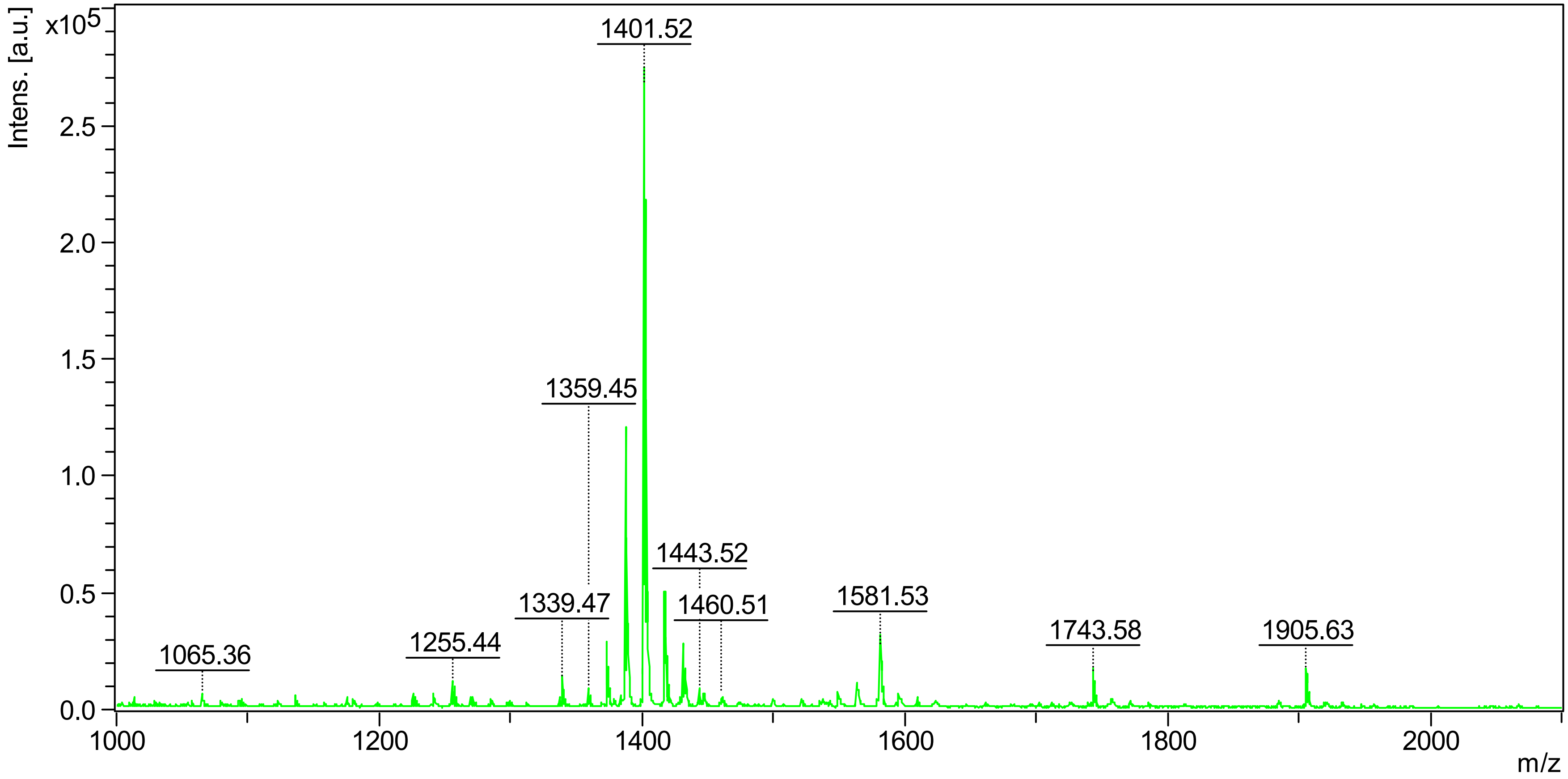

RM171212_Helga

Hel

Hel C-65

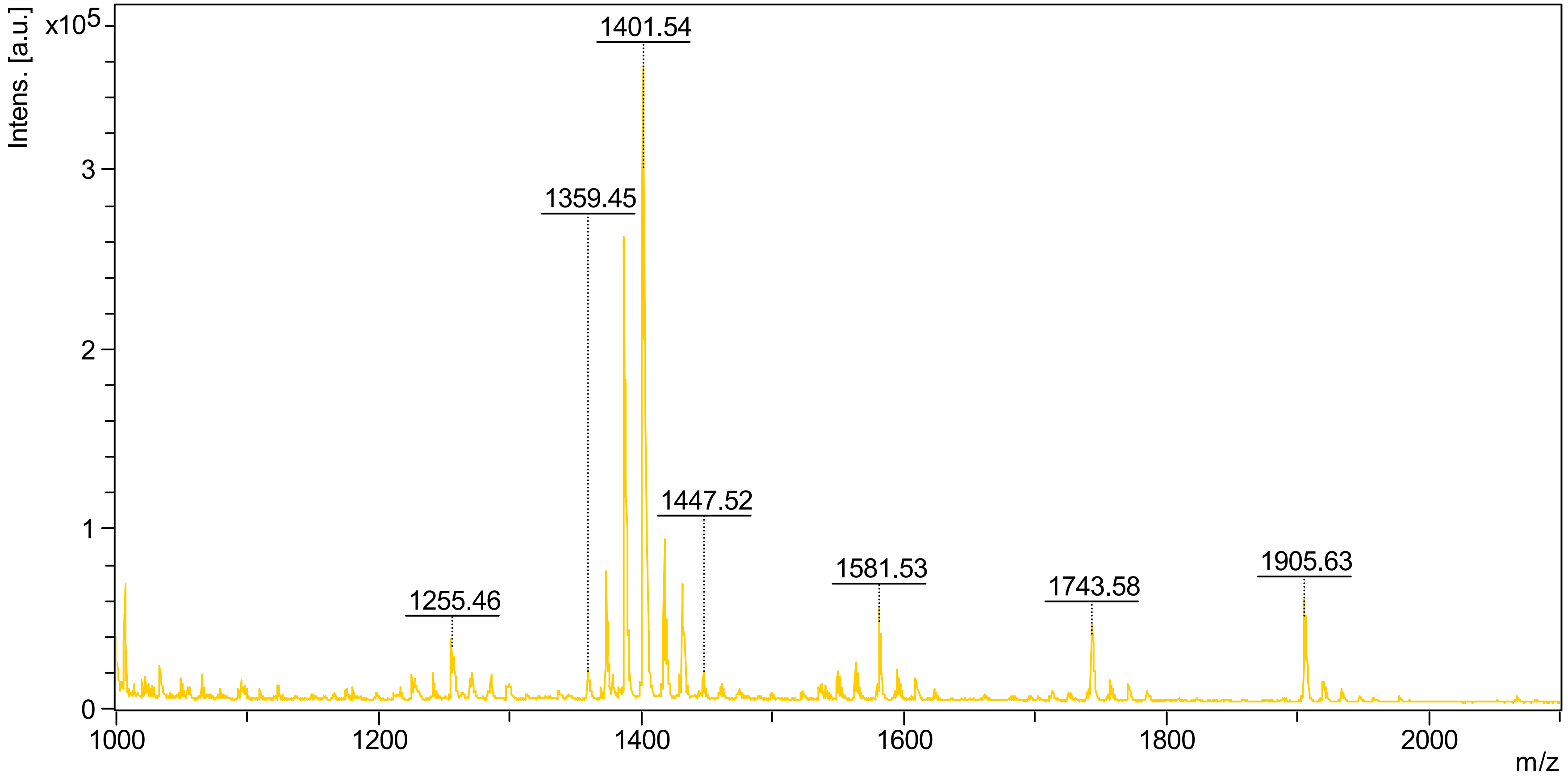

Dr Koll_Alge 45

Hel

Hel C-83

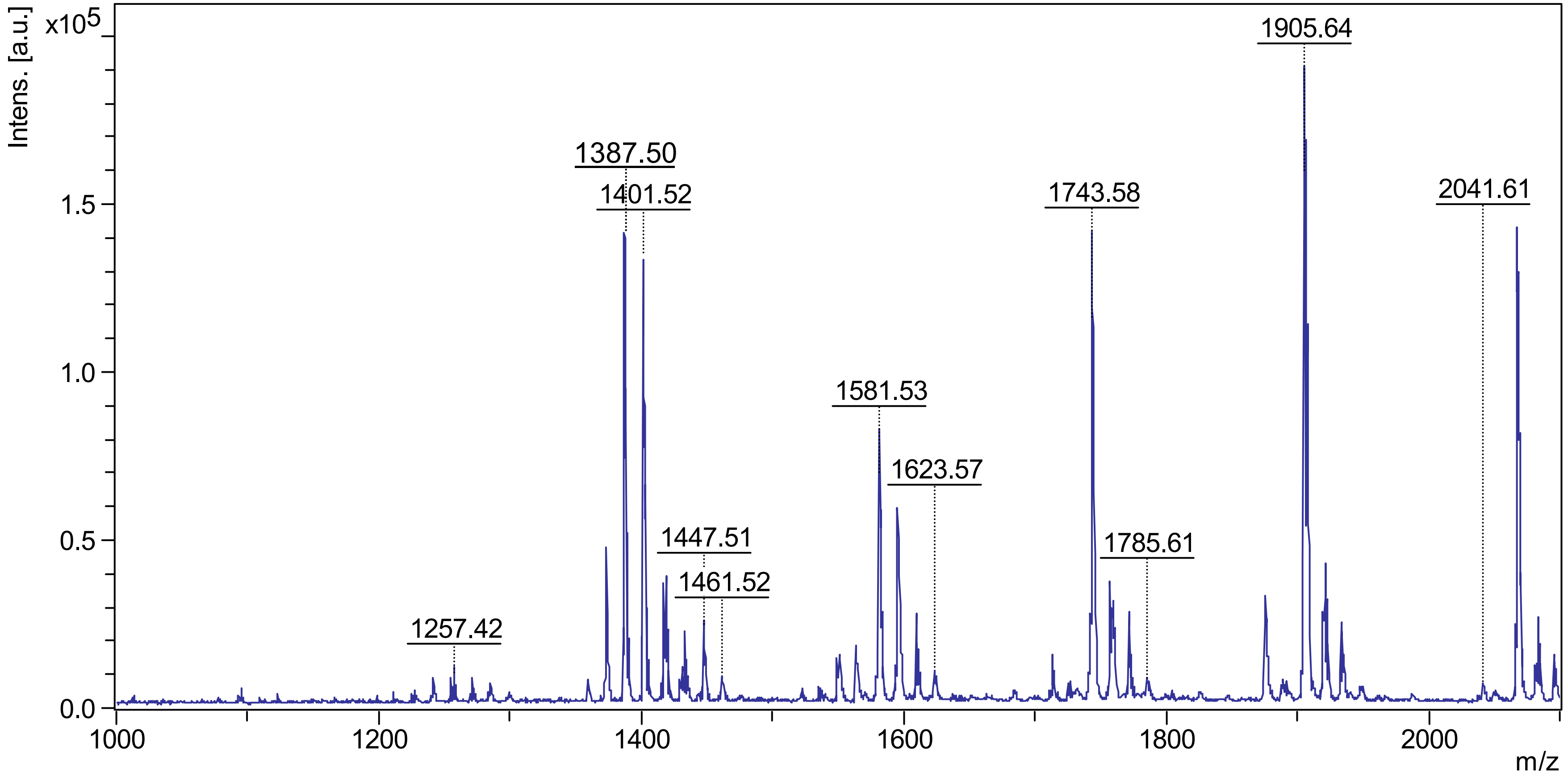

Alge 83 Phycom sorok

Jos

Jos C-23

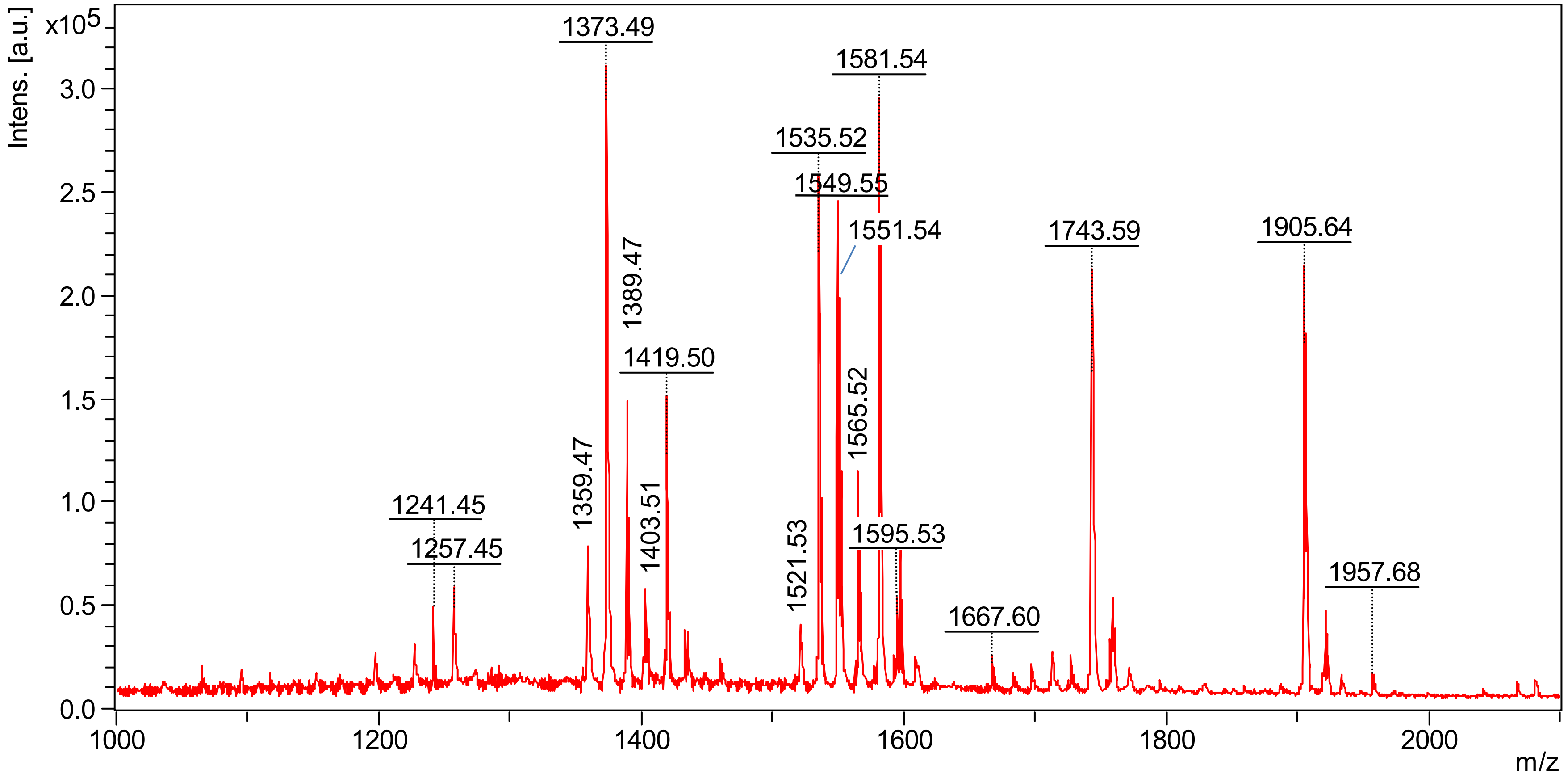

RM_171002_Josefs_new

Jos

Jos C-24

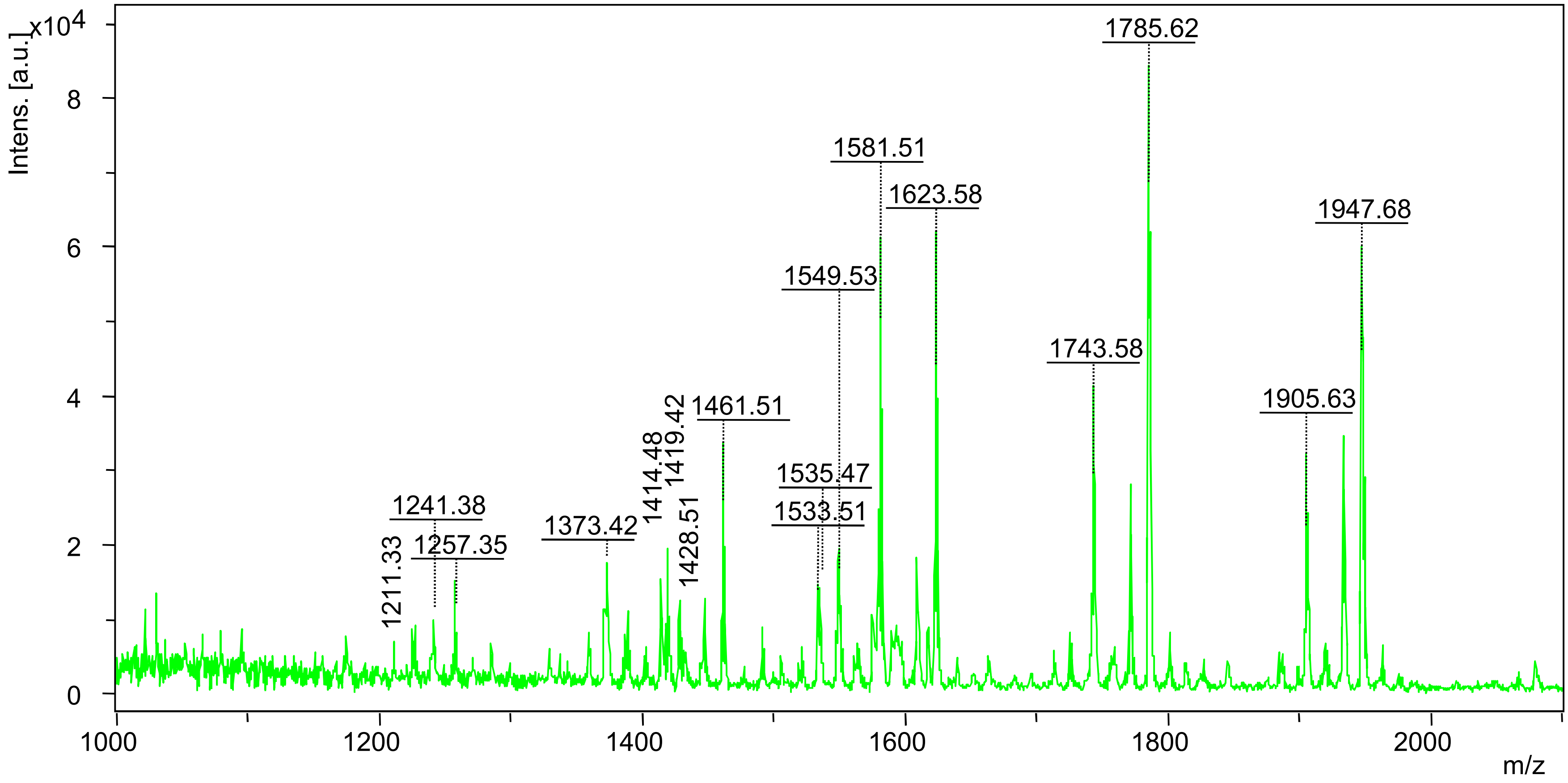

Wellnest

Jos

Jos C-29

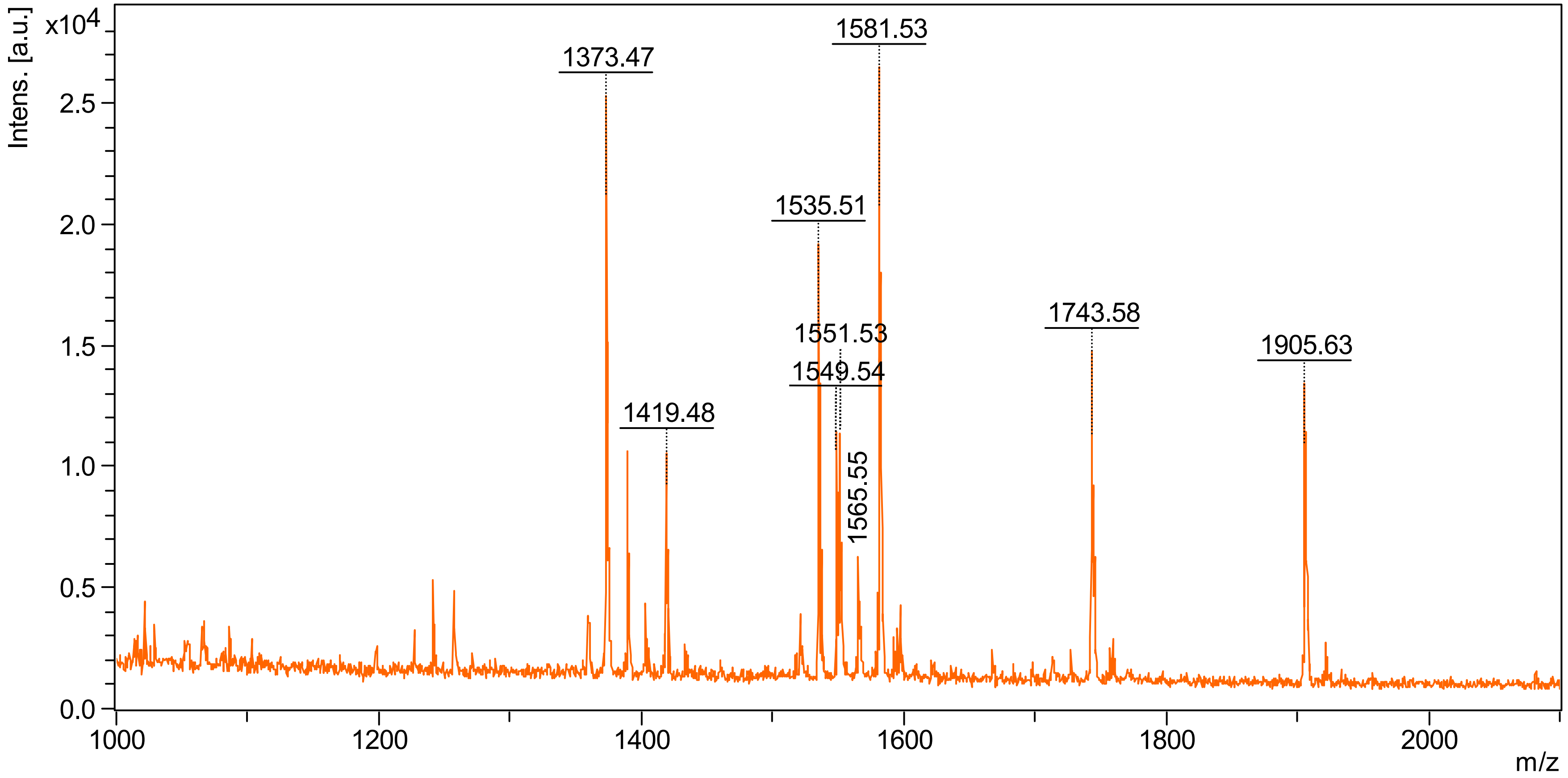

RM170914_EssentialFoods

Raa

Raa C-6

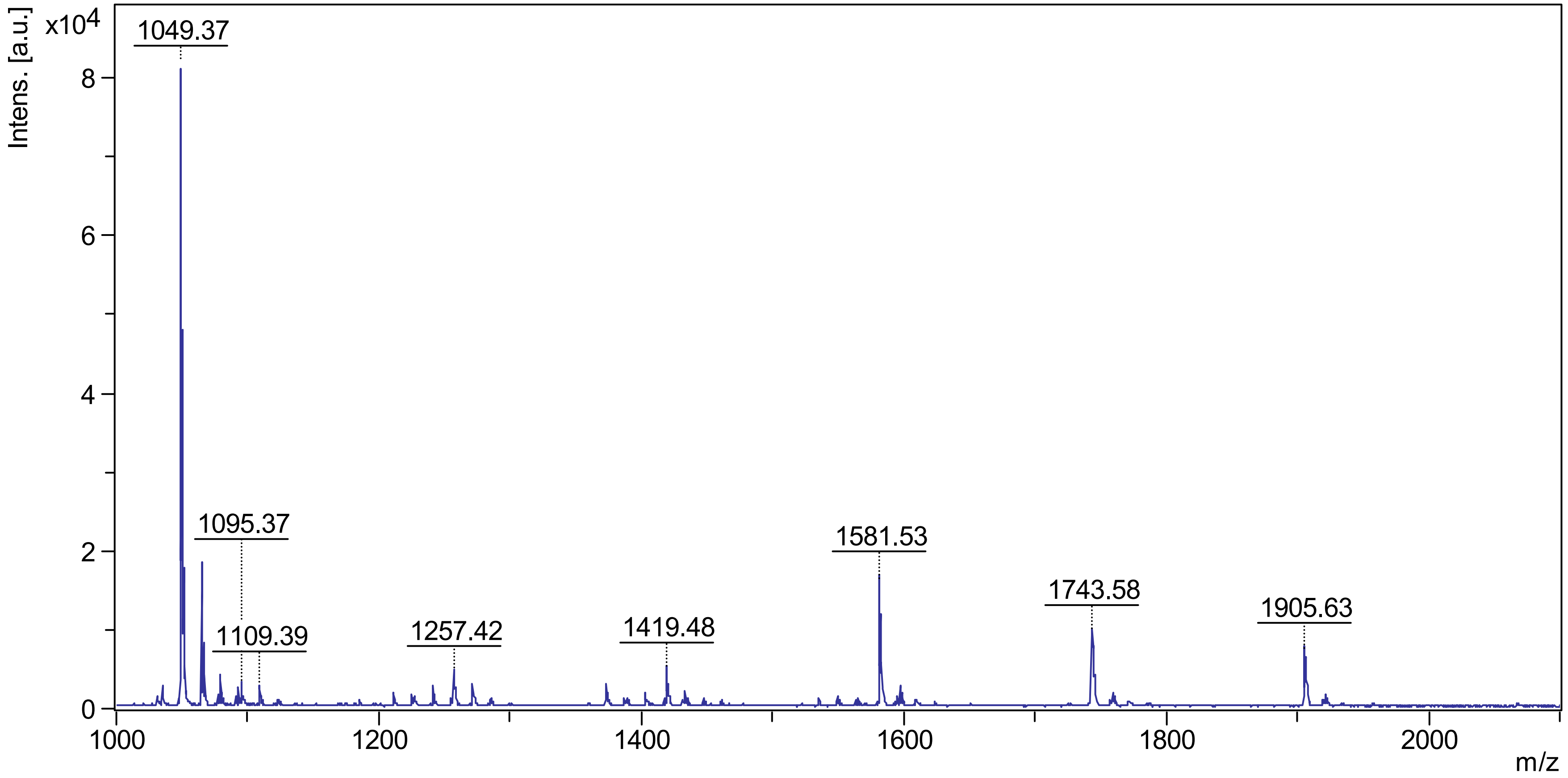

Green Foods LIFT

Raa

Raa C-55

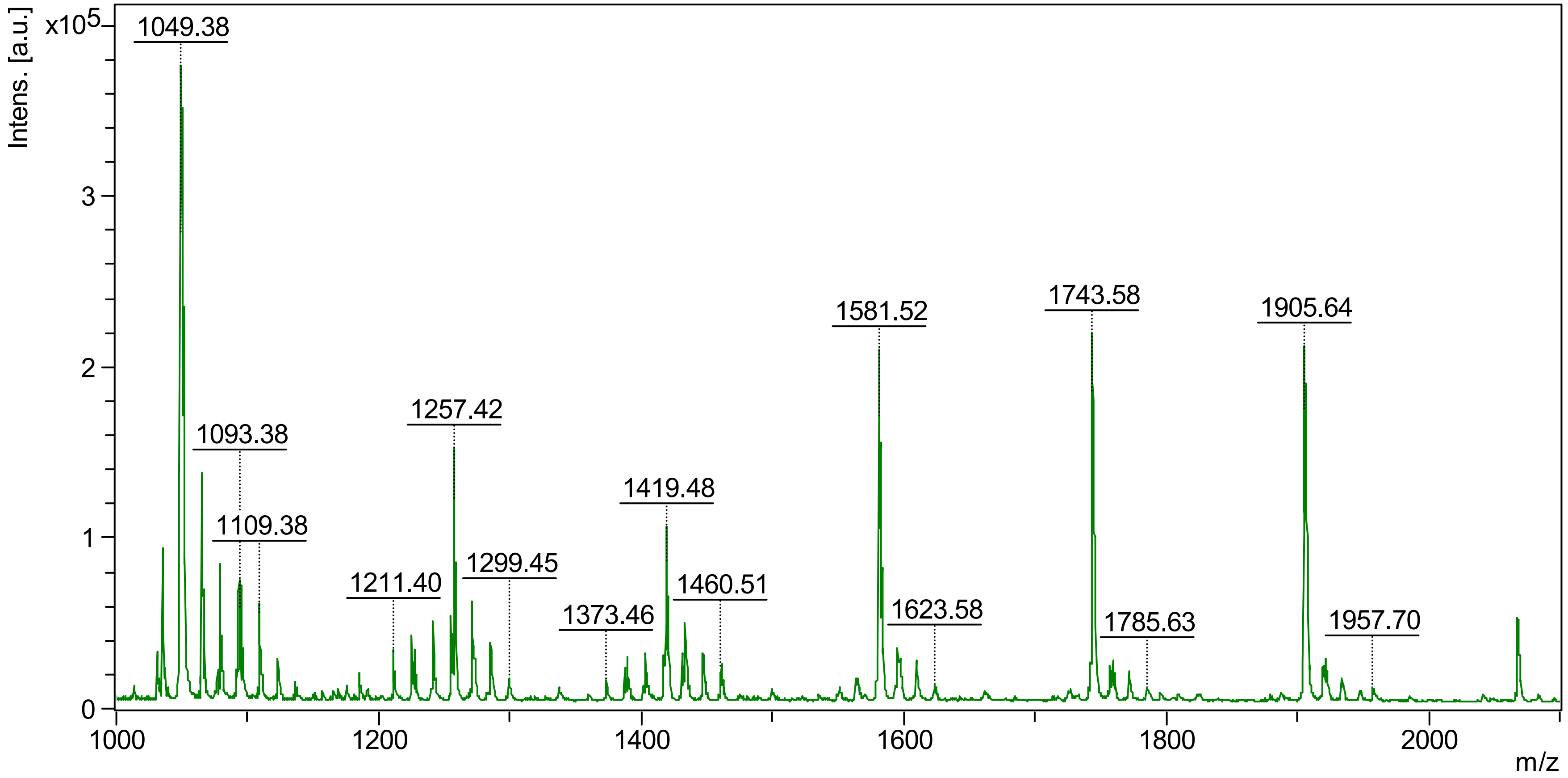

Alge 35_Raab

Raa

Raa C-60

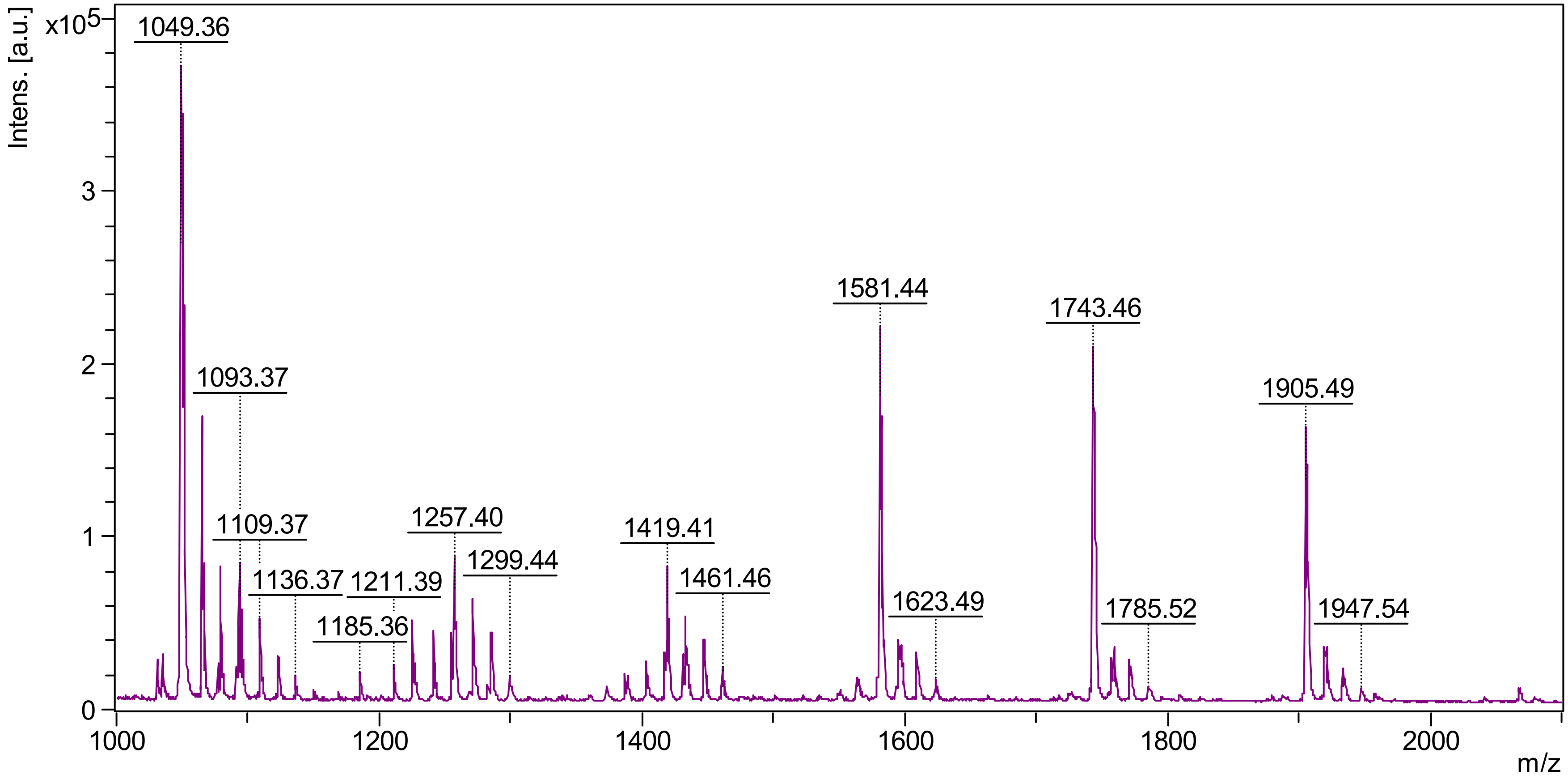

Alge 40_Ehn

Raa

Raa C-63

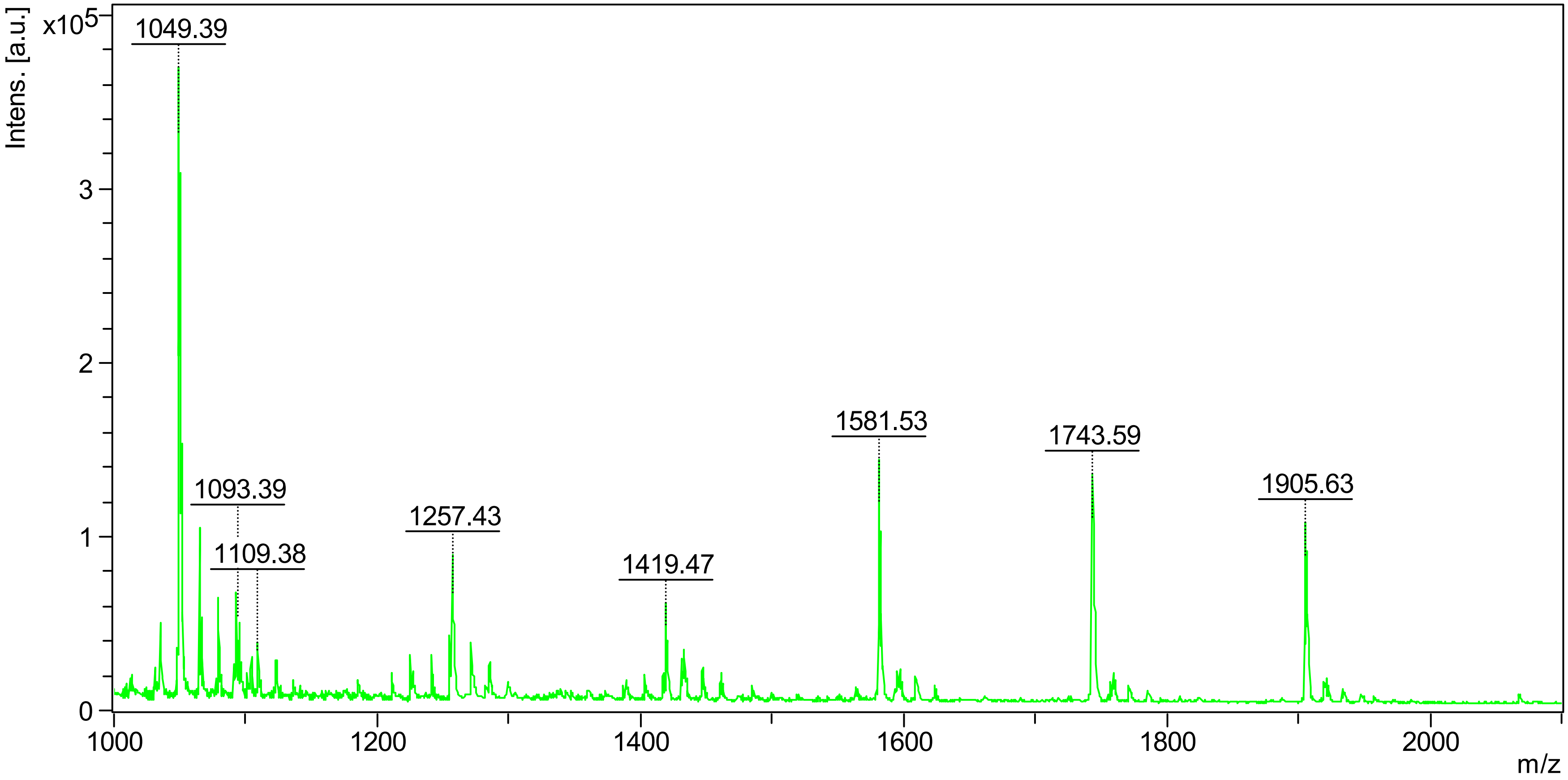

Alge 43_Bioprophyl

Raa

Raa C-64

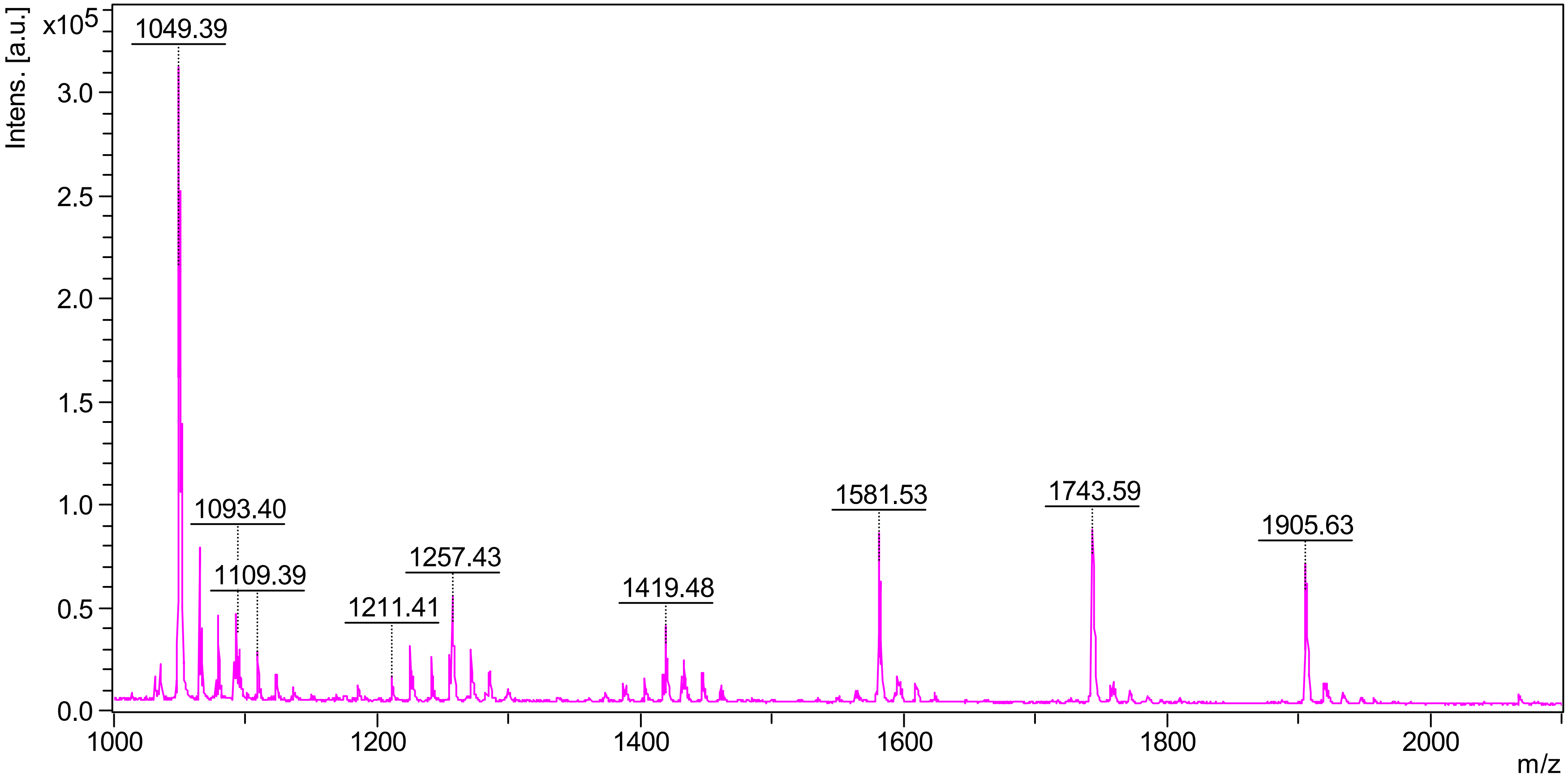

Alge 44 a Tierra Verde

Raa

Raa C-68

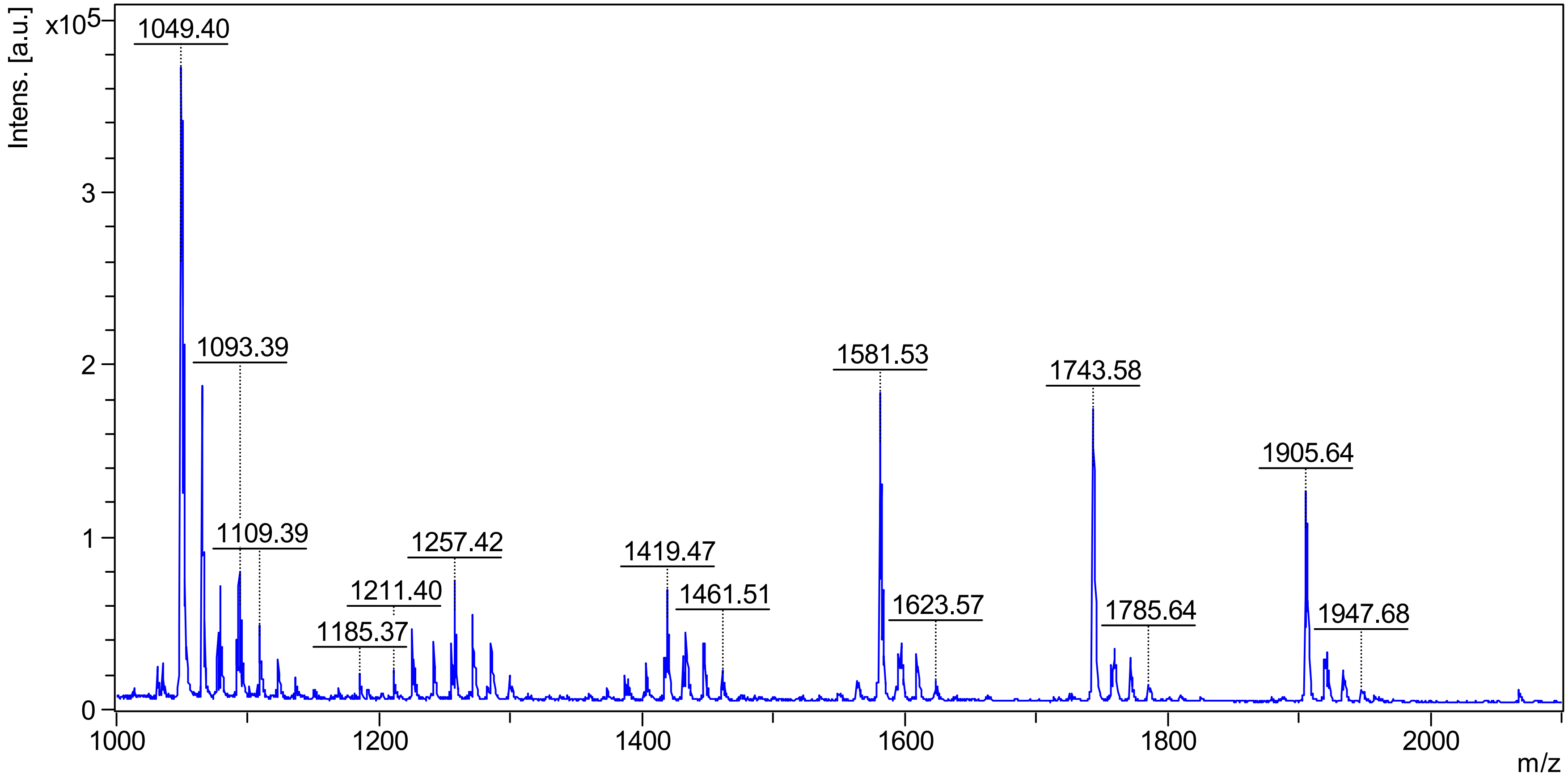

Alge 48_Medicura

Raa

Raa C-70

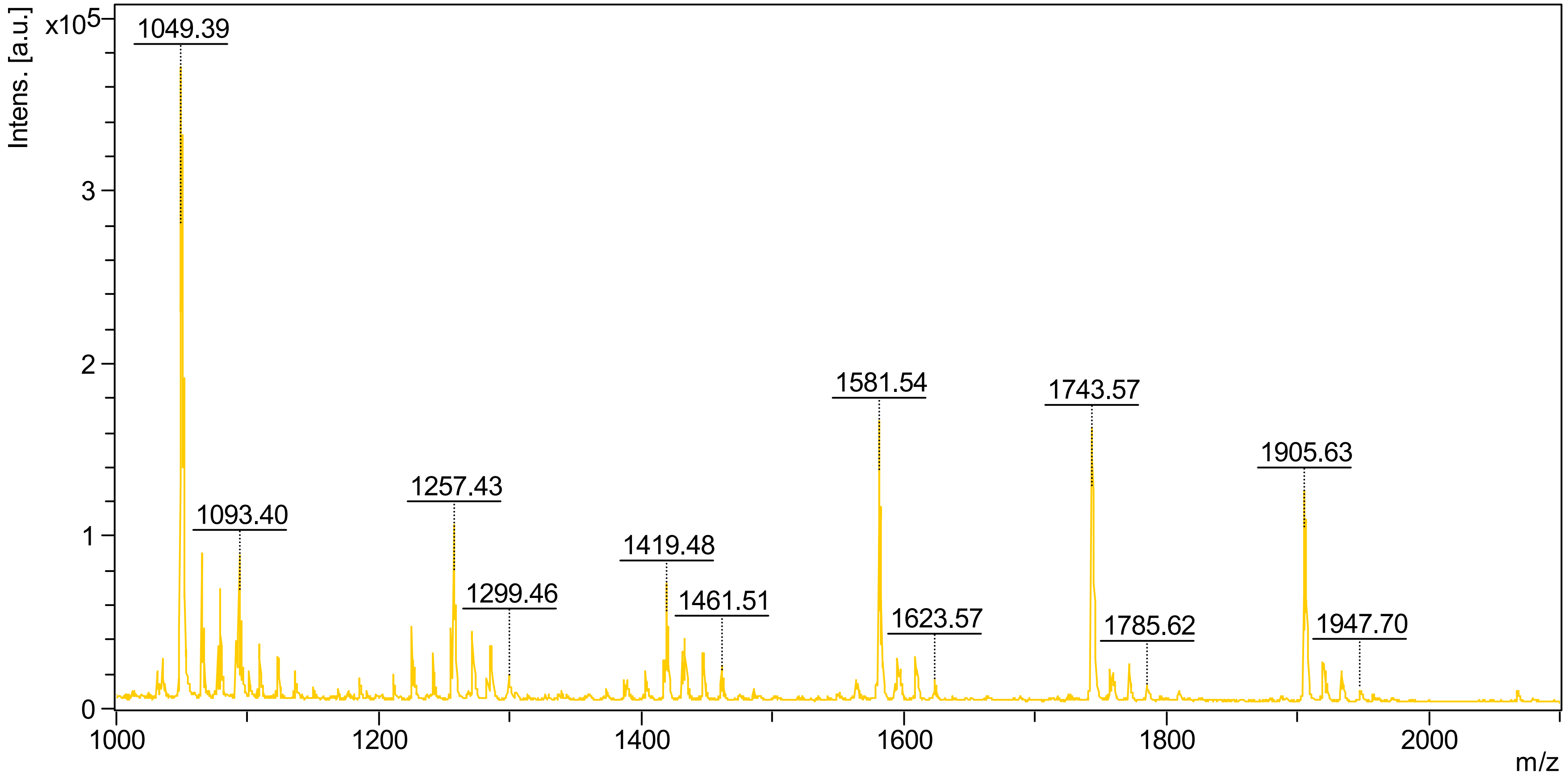

Alge 50 green foods

Raa

Raa C-76

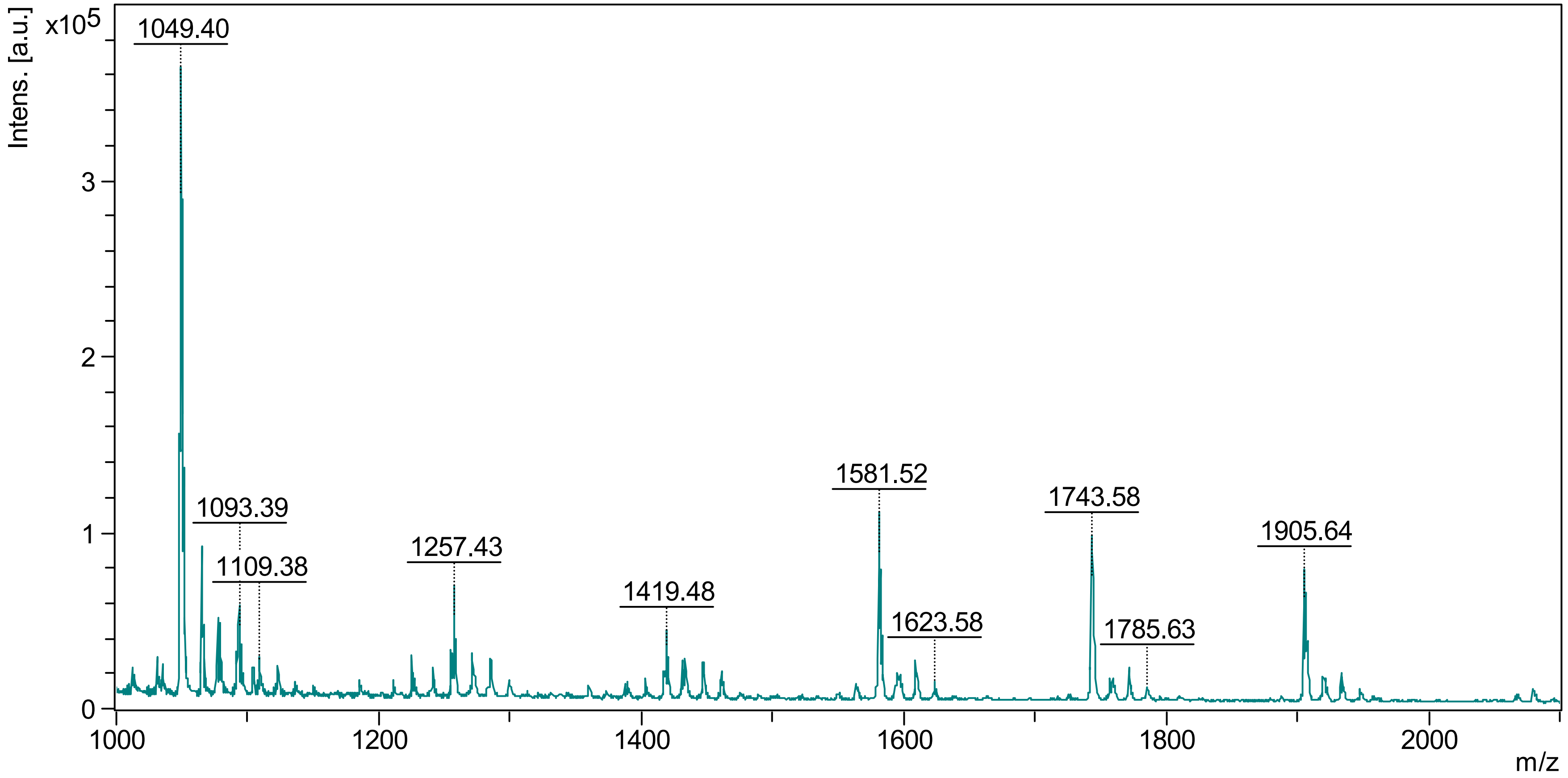

Alge 76 Amlawell

Now

Now C-5

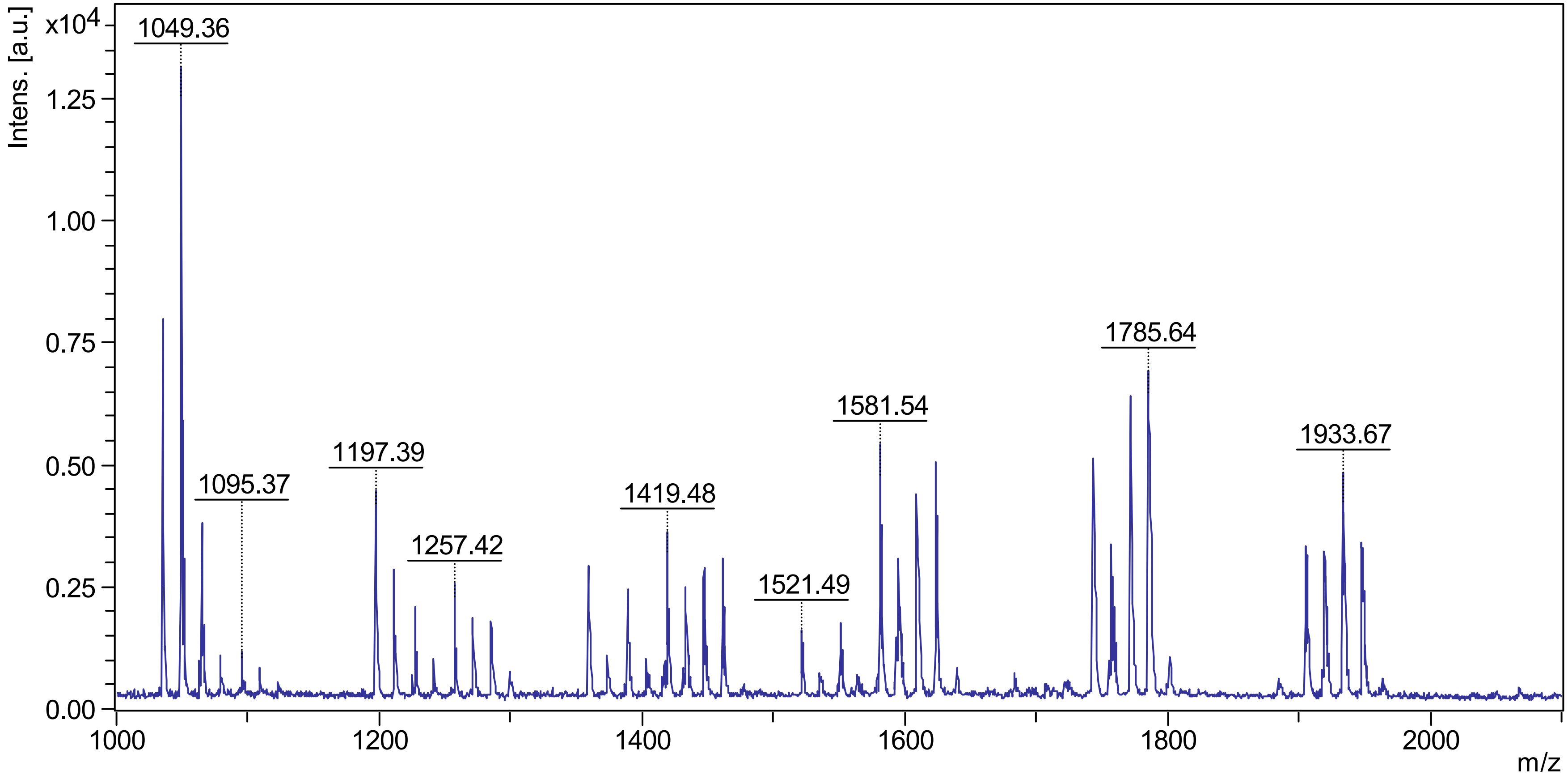

Now Foods LIFT

Sun

Sun C-16

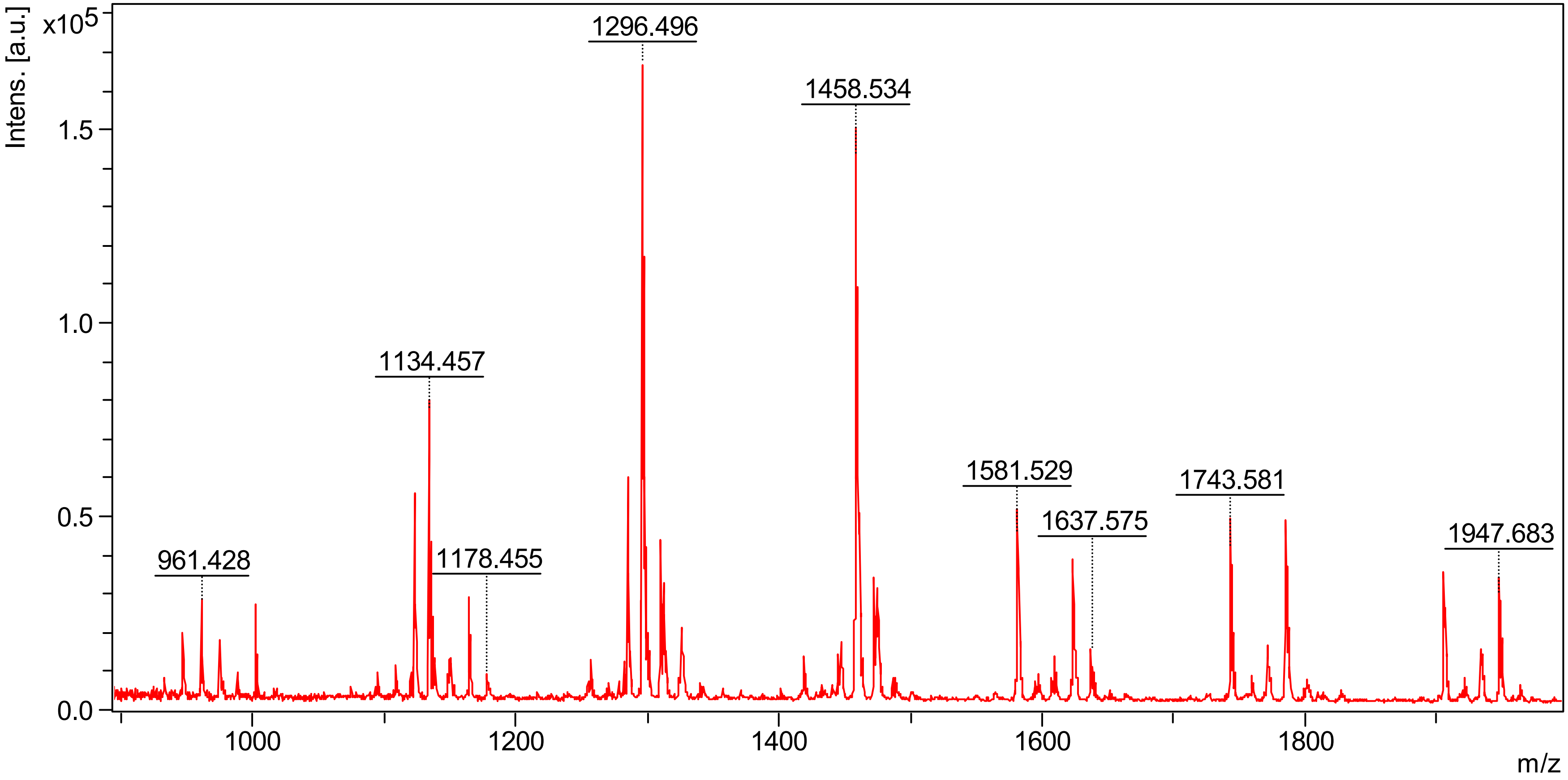

RM_171002_Sun_new

Sun

Sun C-36

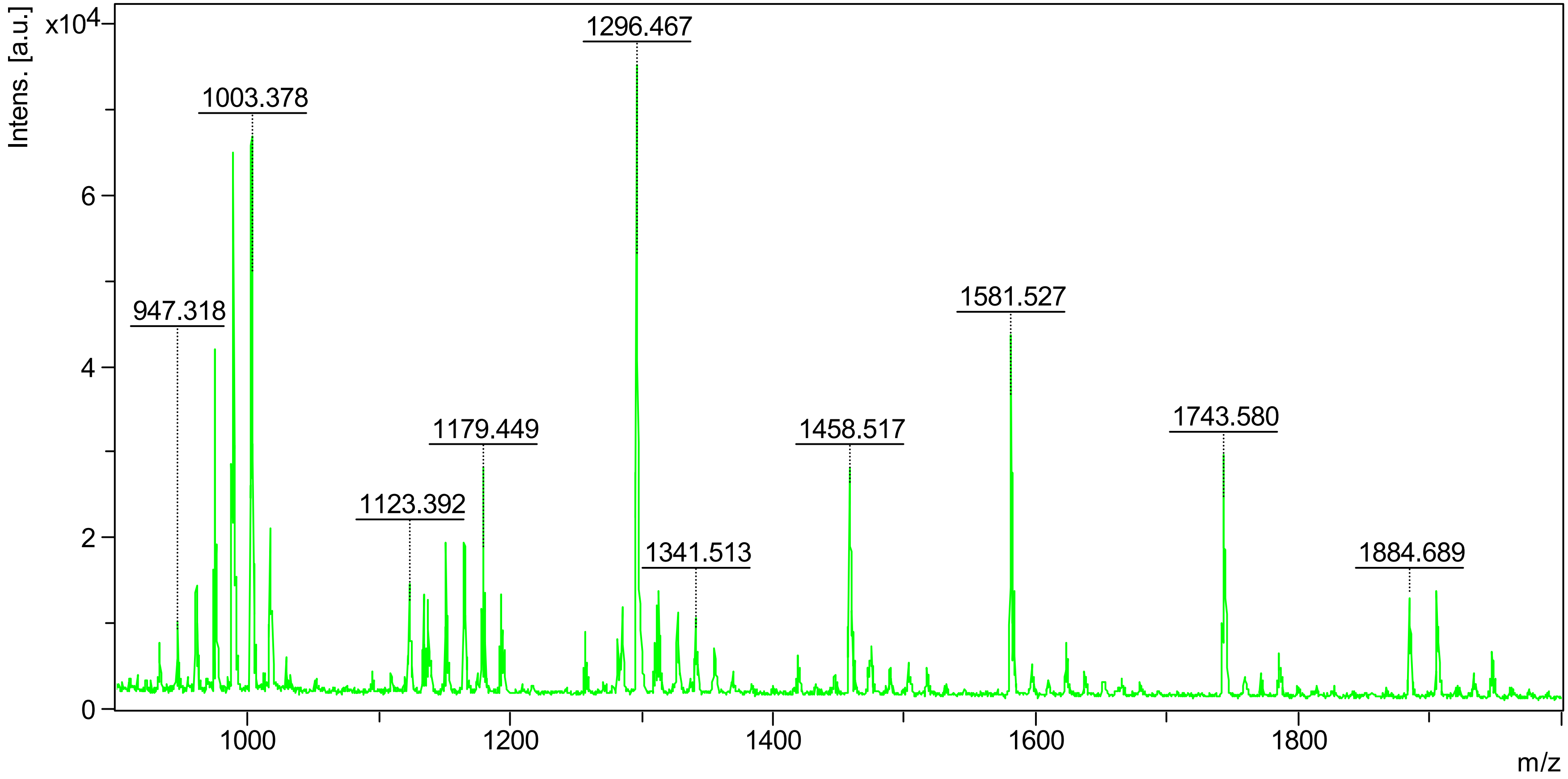

TopInForm

Ori

Ori C-9

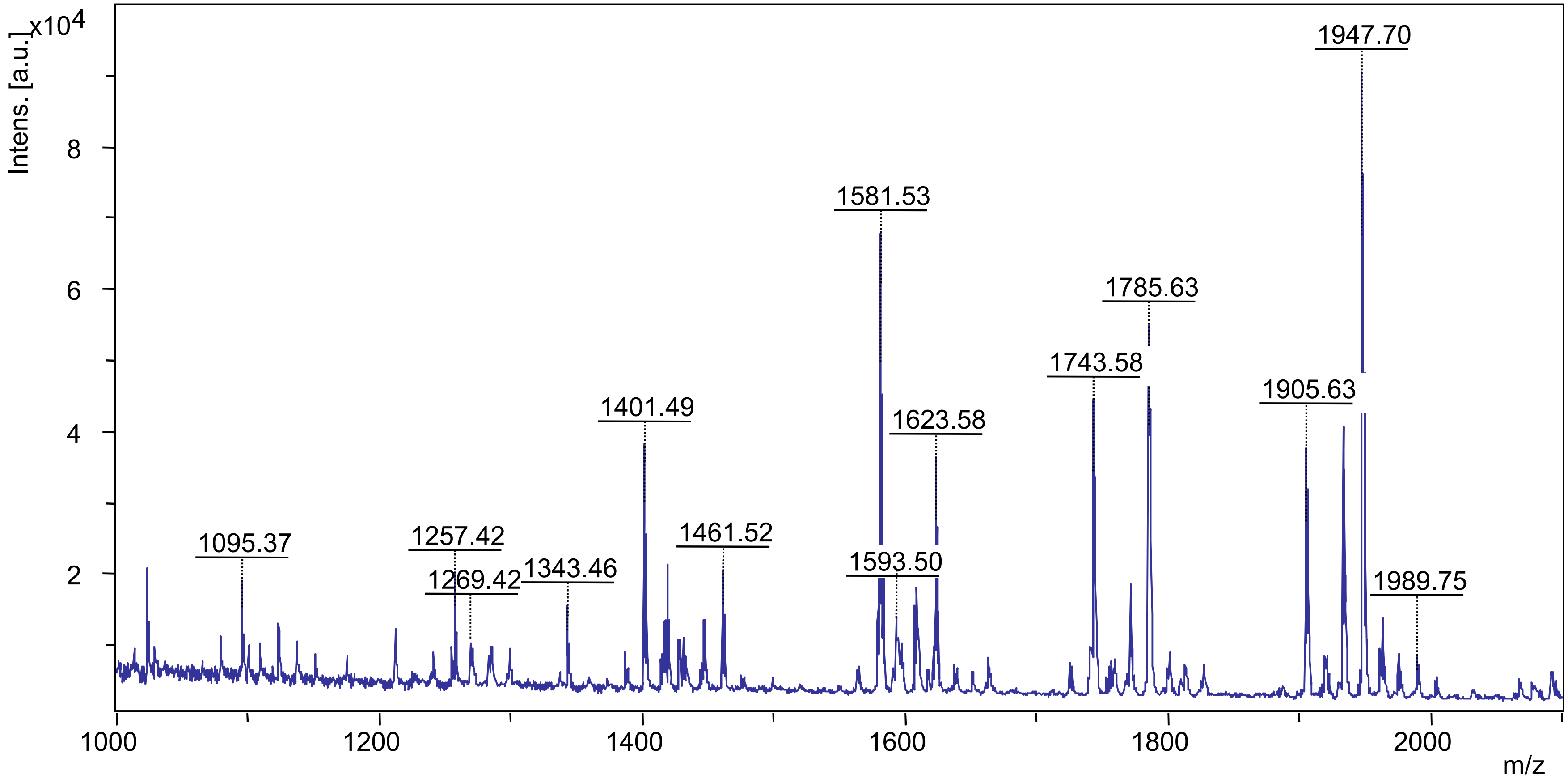

Mai Alge 9 Sunfood

Ori

Ori C-27

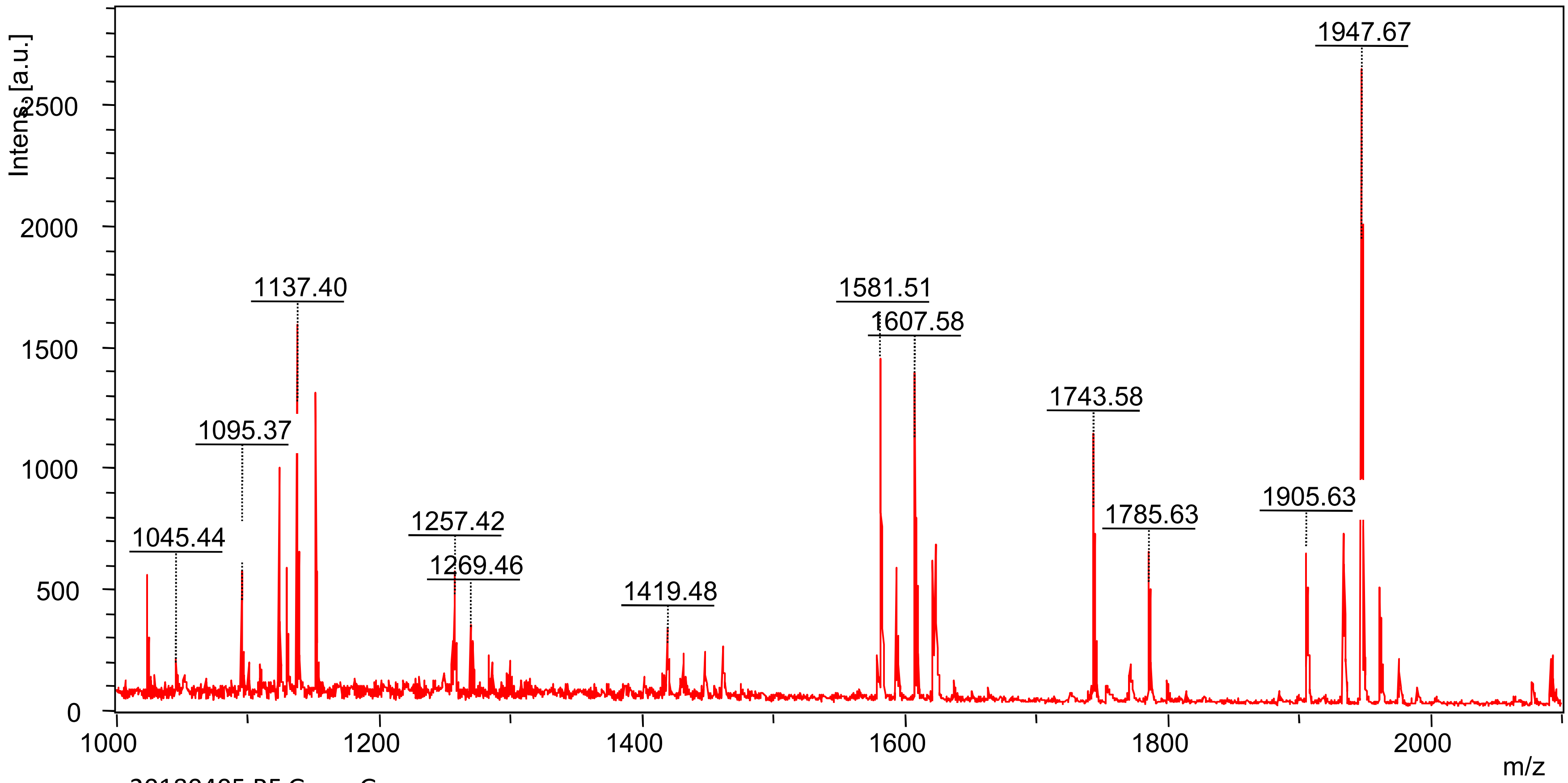

20180405 RF GreenGem

Ori

Ori C-28

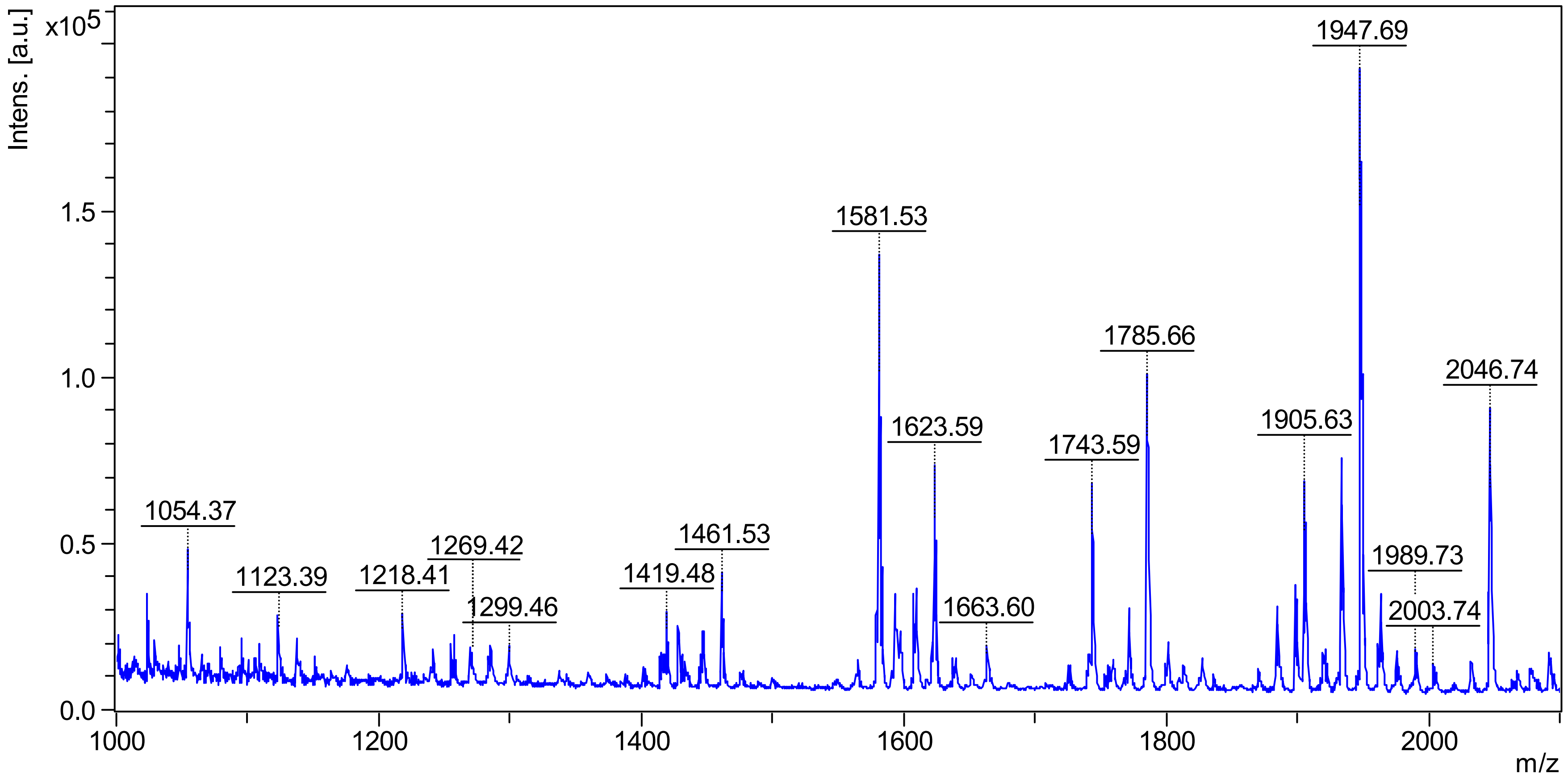

Bio-Insel wh

Ori

Ori C-31

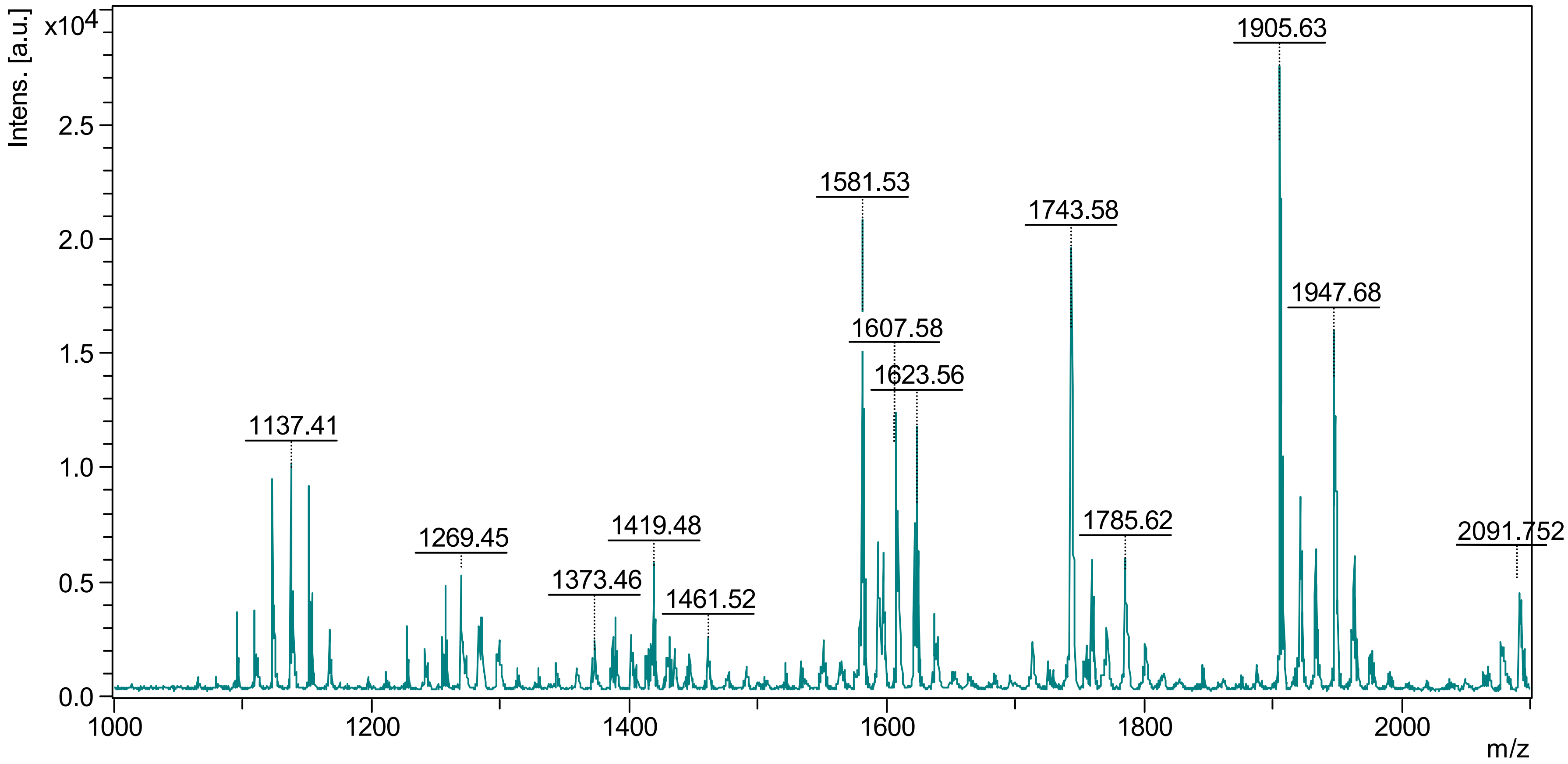

Pure Planet Apr19

Ori

Ori C-46

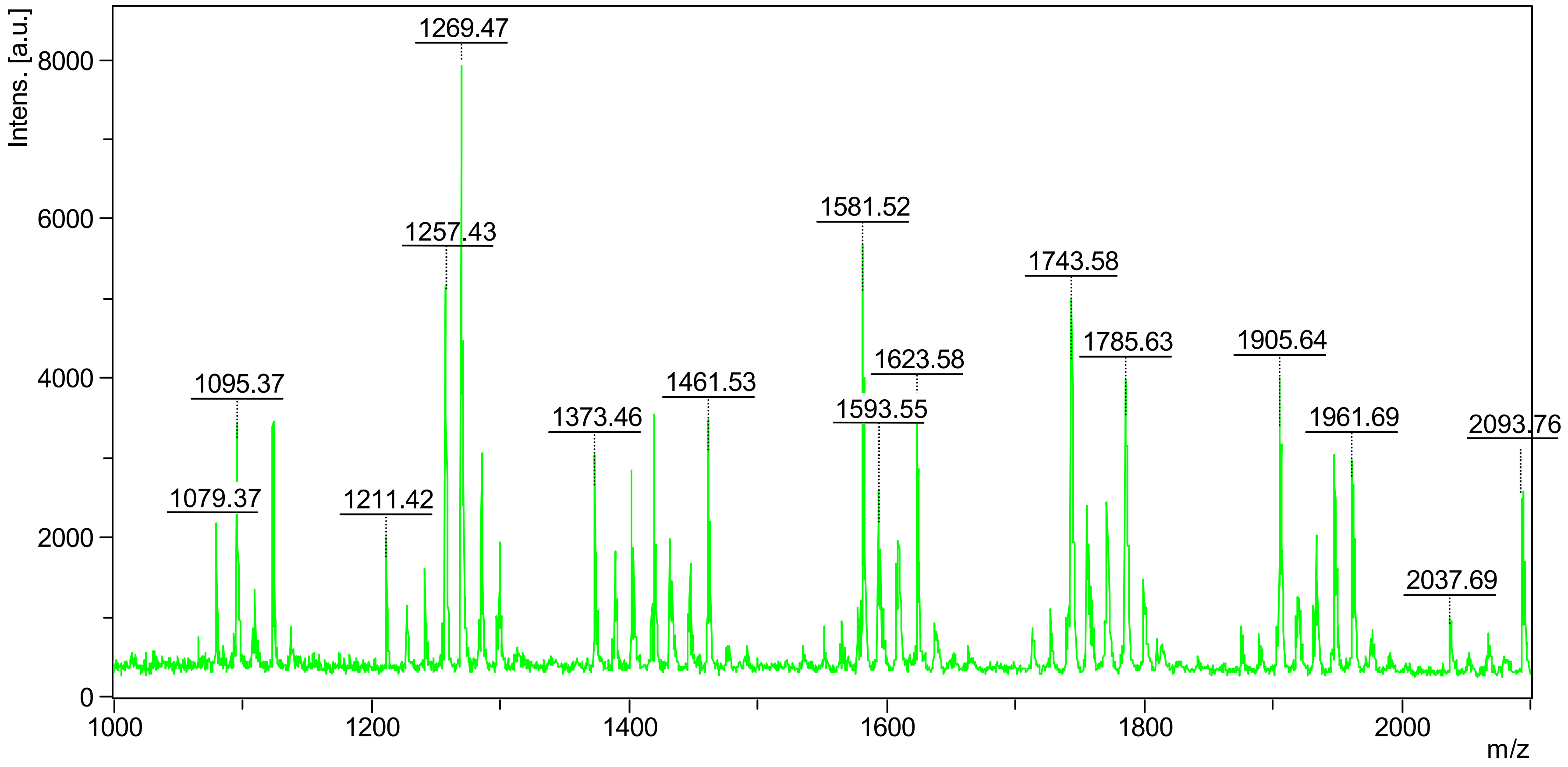

Healthy Origins LIFT

Ori

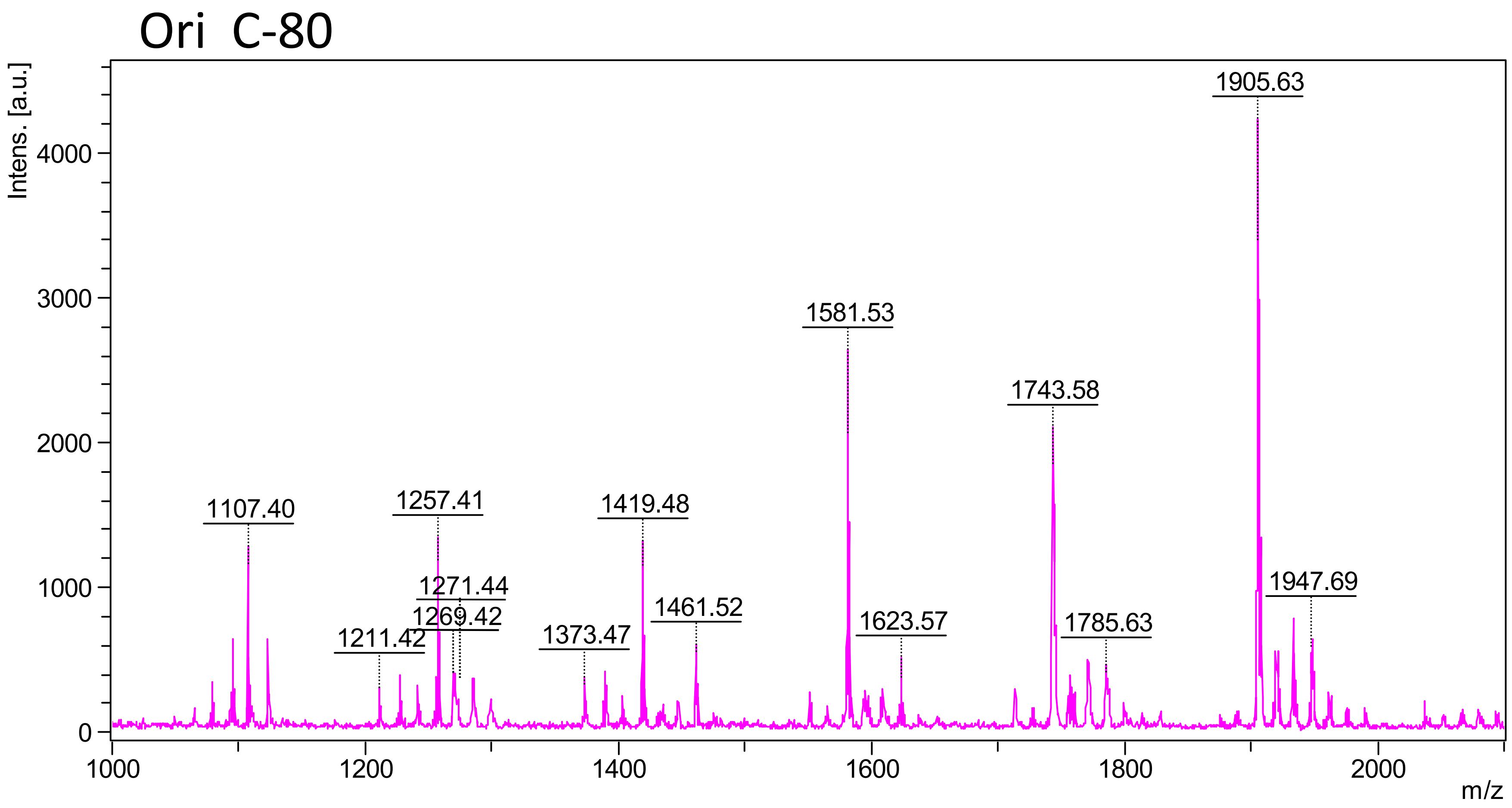

Alge 80 Sunday Natural

Ori

Ori C-82

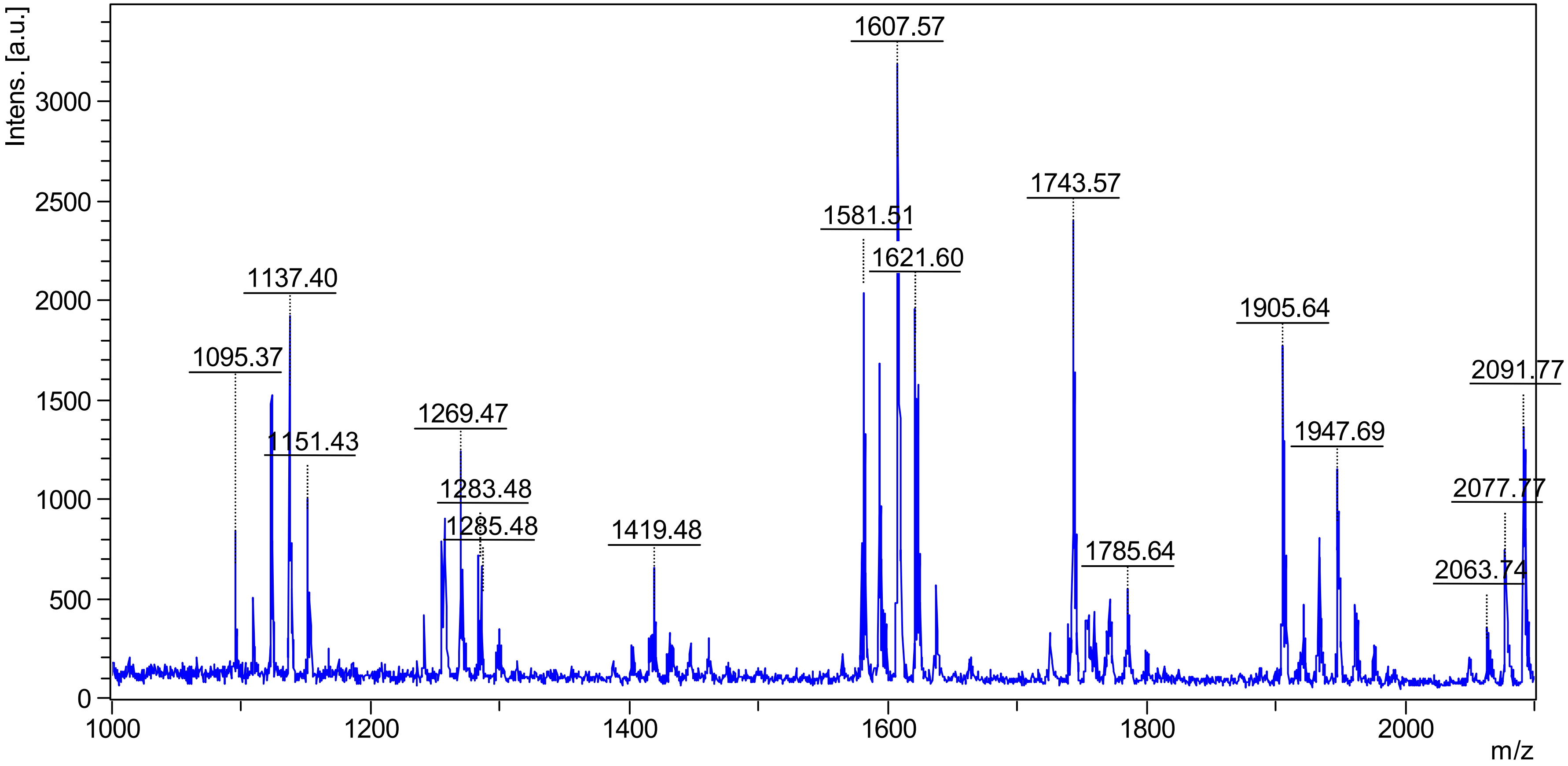

Alge 82b Vitalplan

Sol

Sol C-3

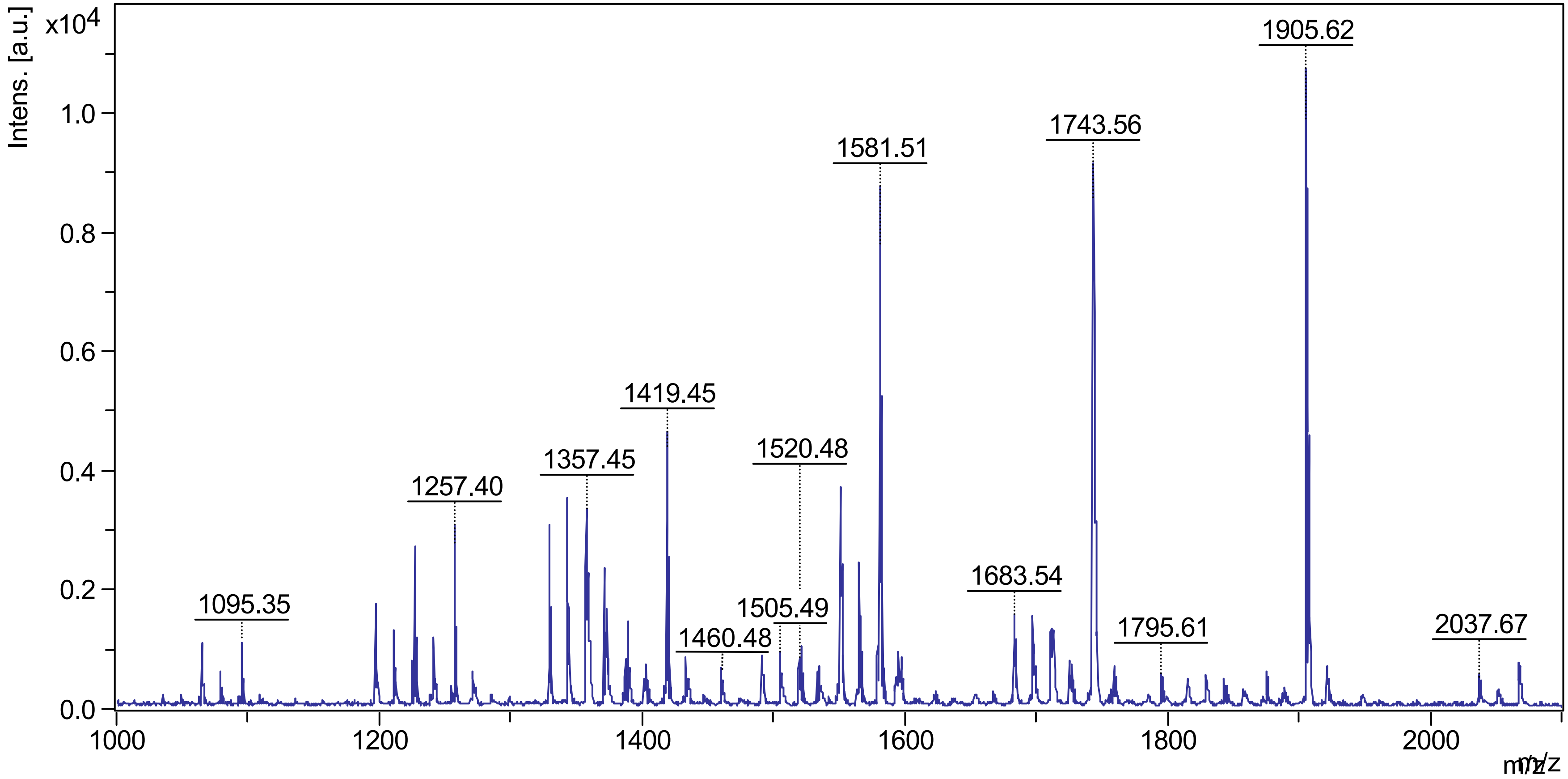

Natures way 1 to 25

Sol

Sol C-4

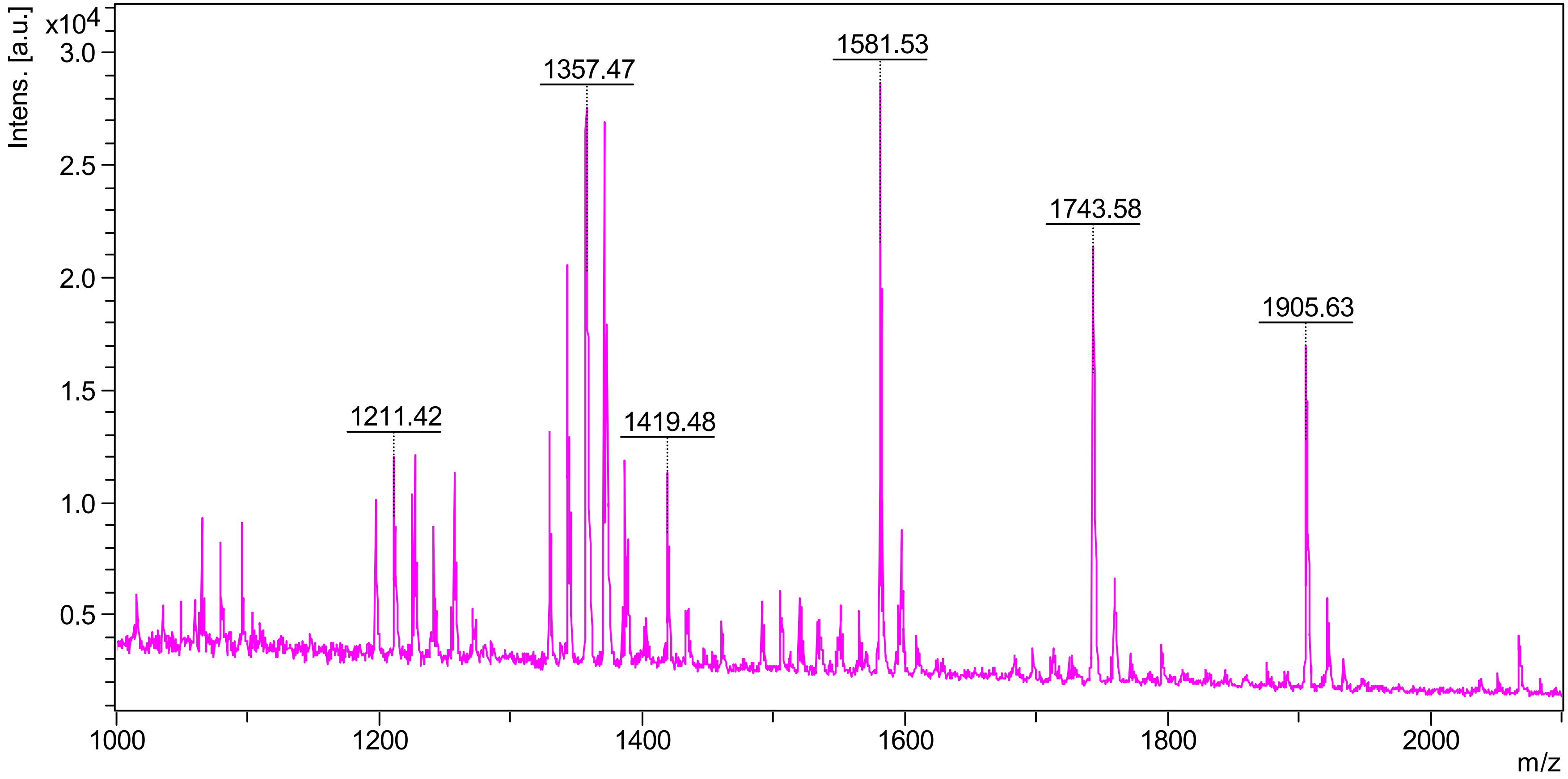

Source Natural from Mai Alge 4

Sol

Sol C-8

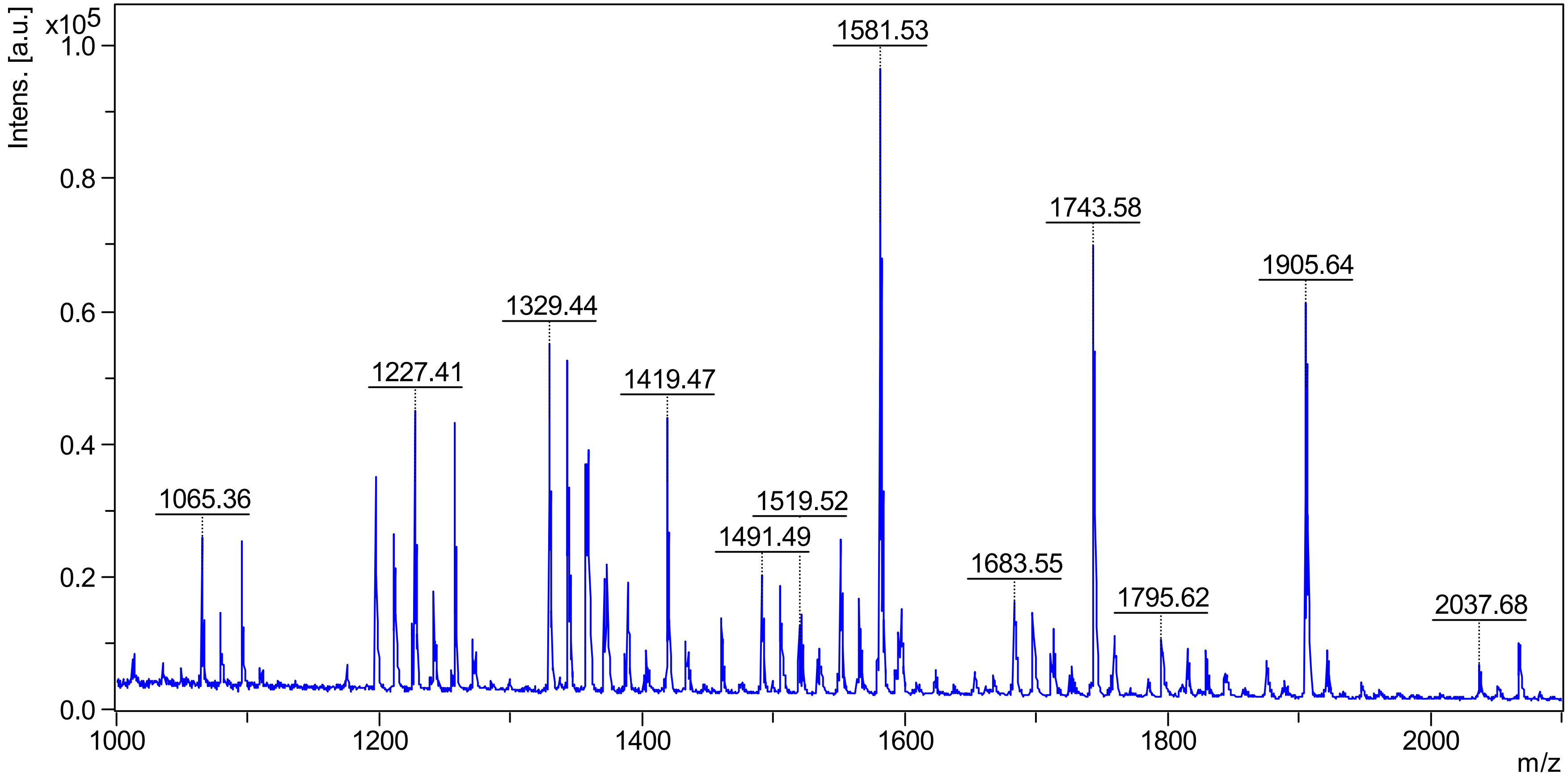

Solgar von Mai Alge 8

Sol

Sol C-10

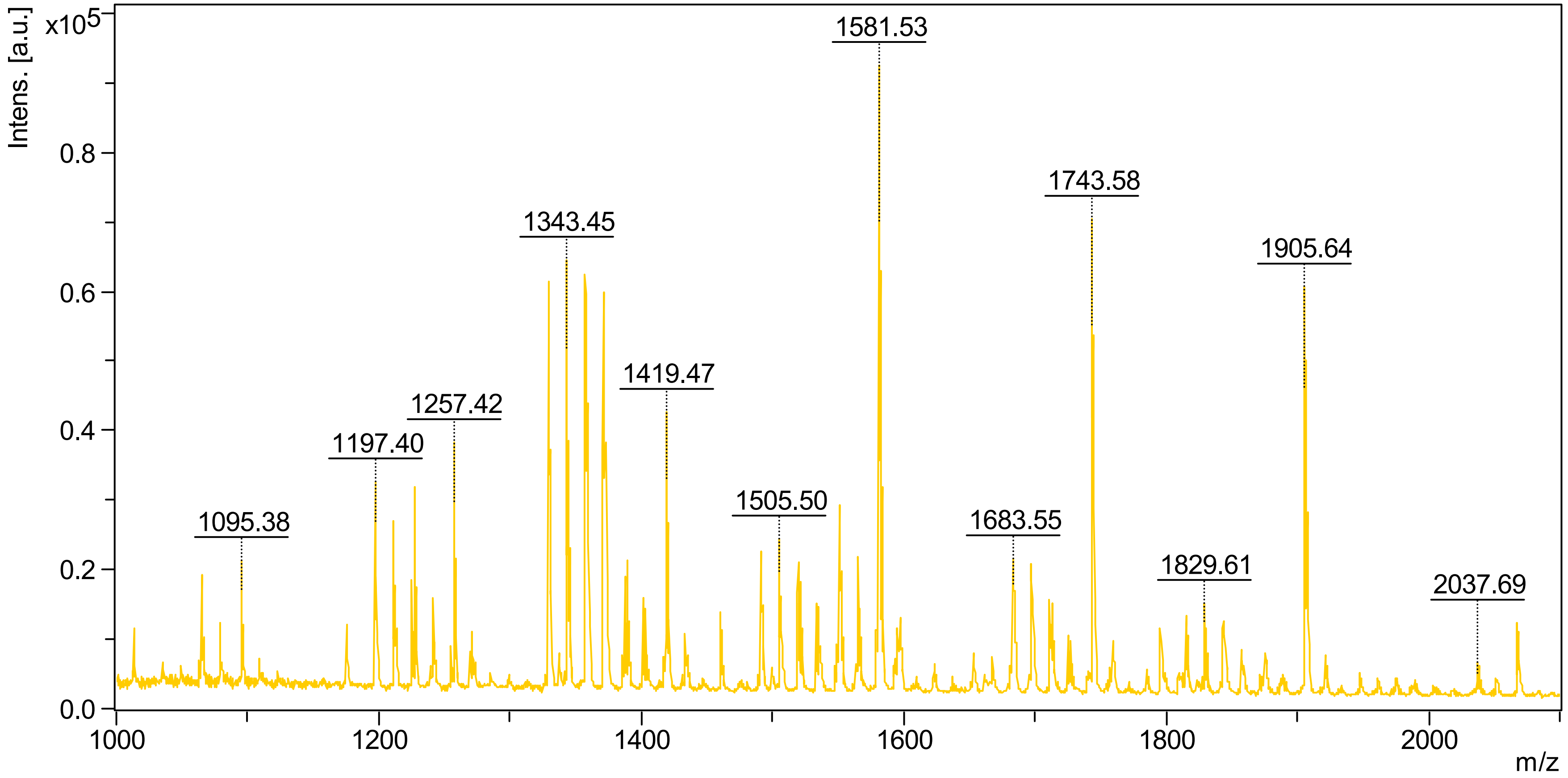

Earth Circle von Mai Alge 10

Sol

Sol C-21

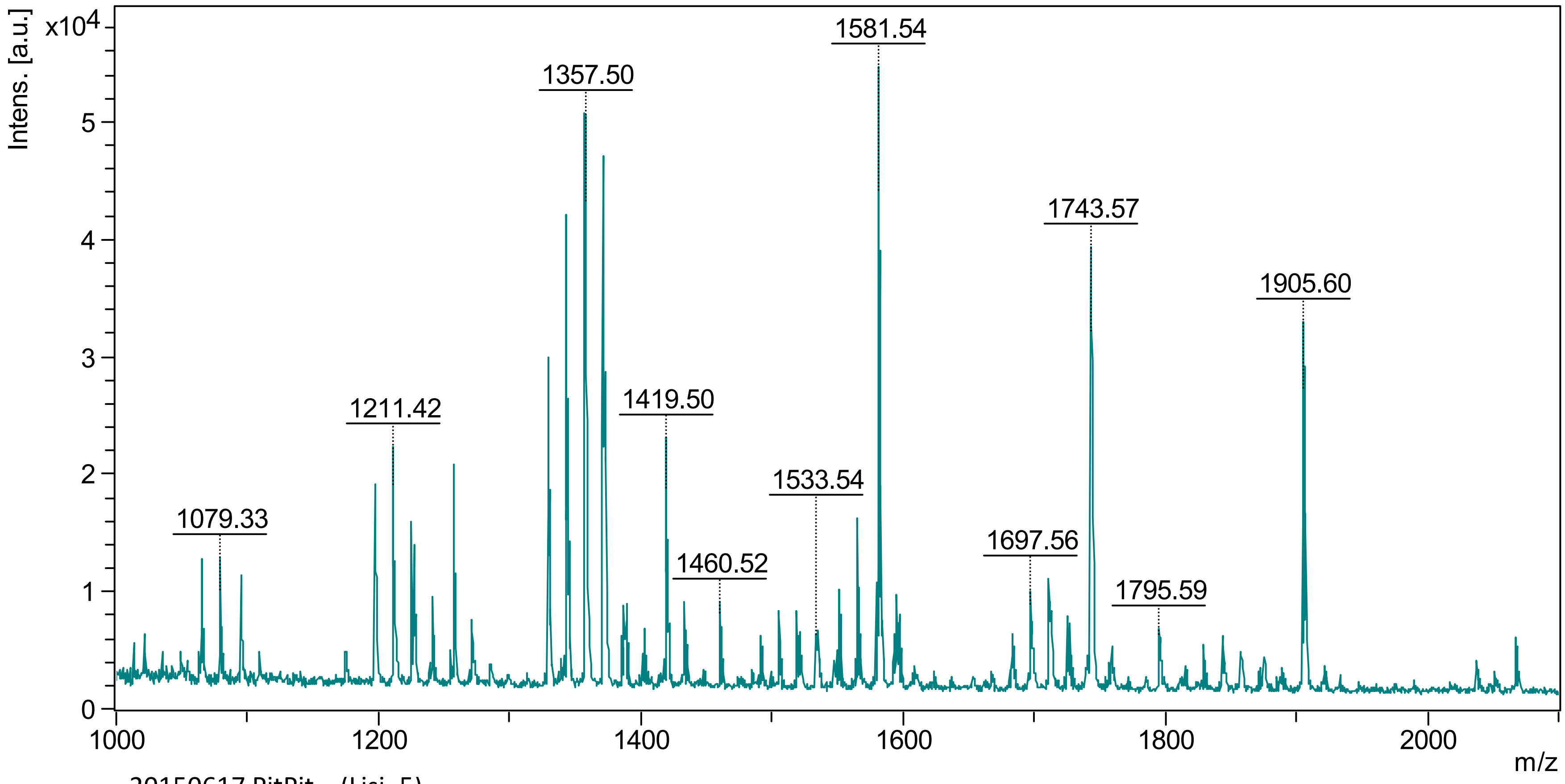

20150617 PitPit (Lisi_5)

Sol

Sol C-22

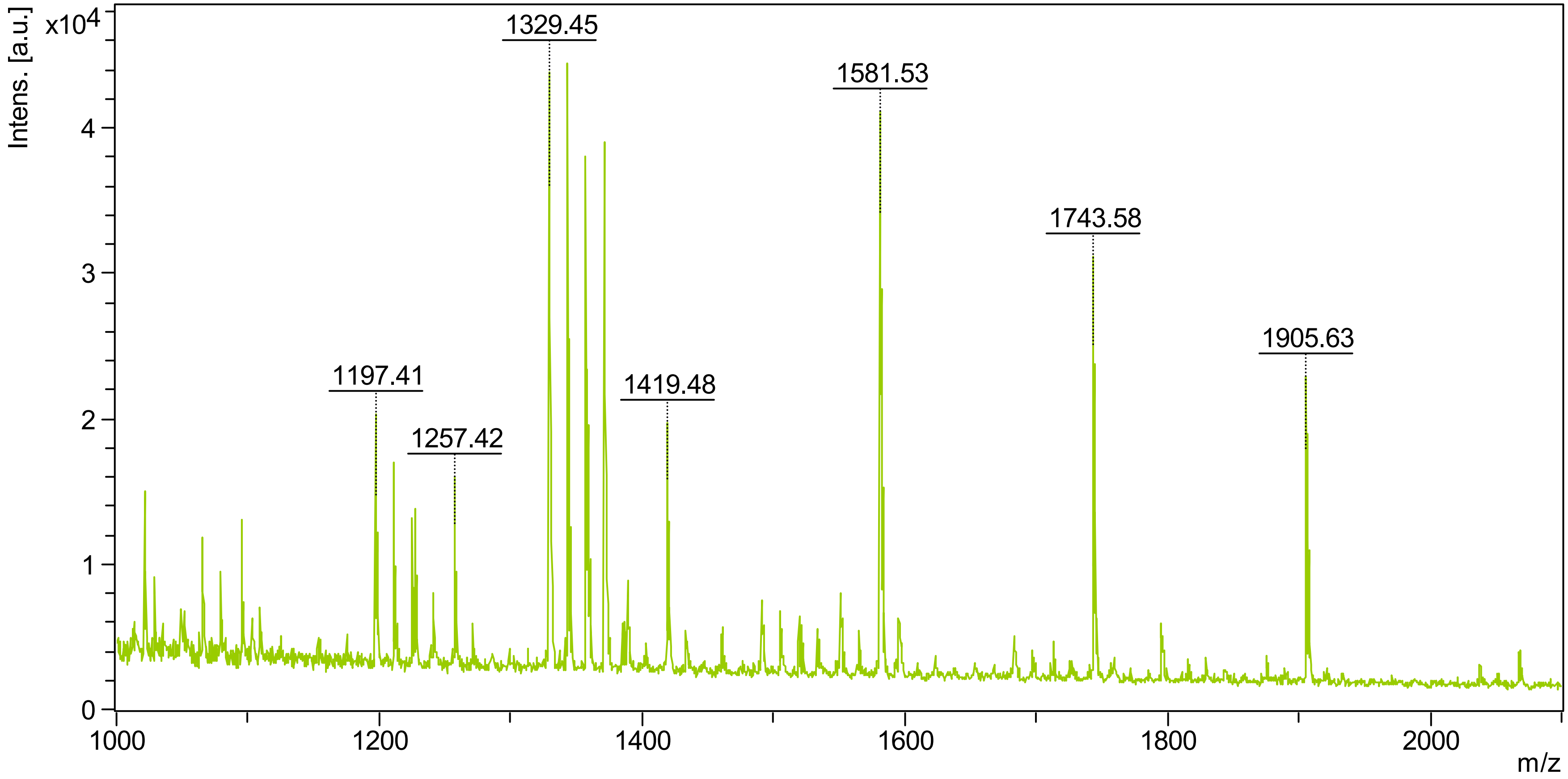

Vitagreen C.P. Protect A

Sol

Sol C-39

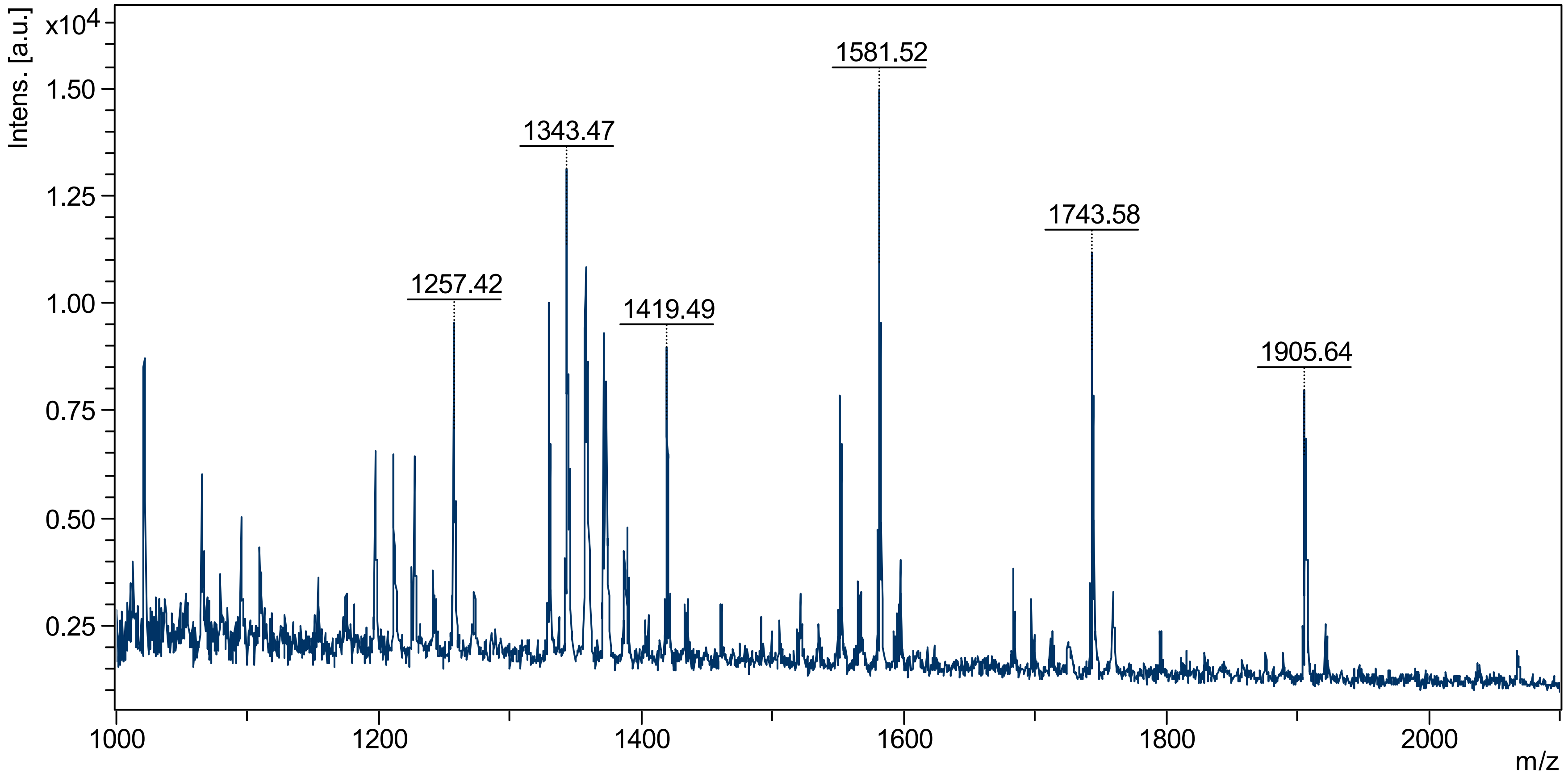

RM_171122_paperbag

Sol

Sol C-47

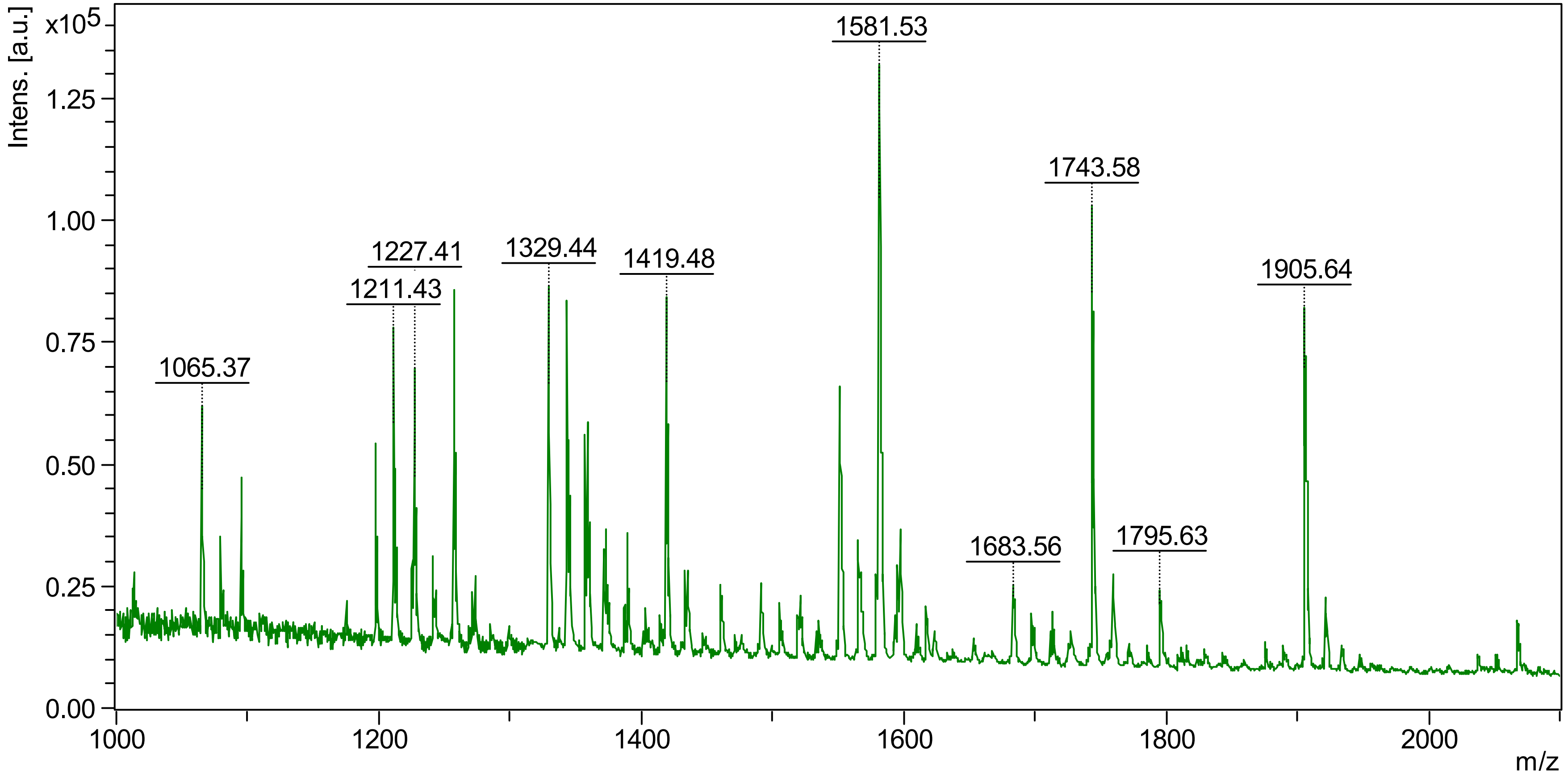

20180418 RF KP SunPortion

Sol

Sol C-53

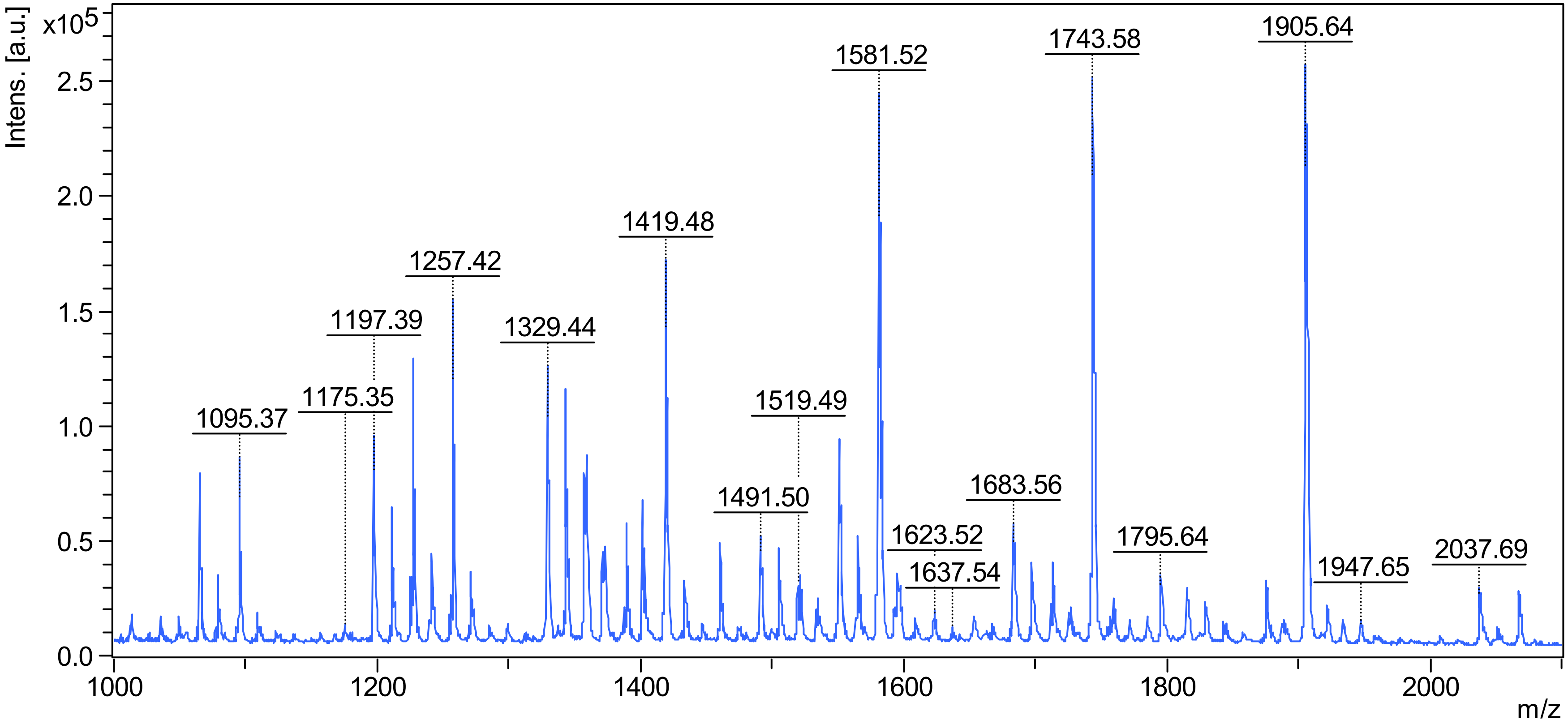

Aurica 53 von Alge 33

Sol

Sol C-69

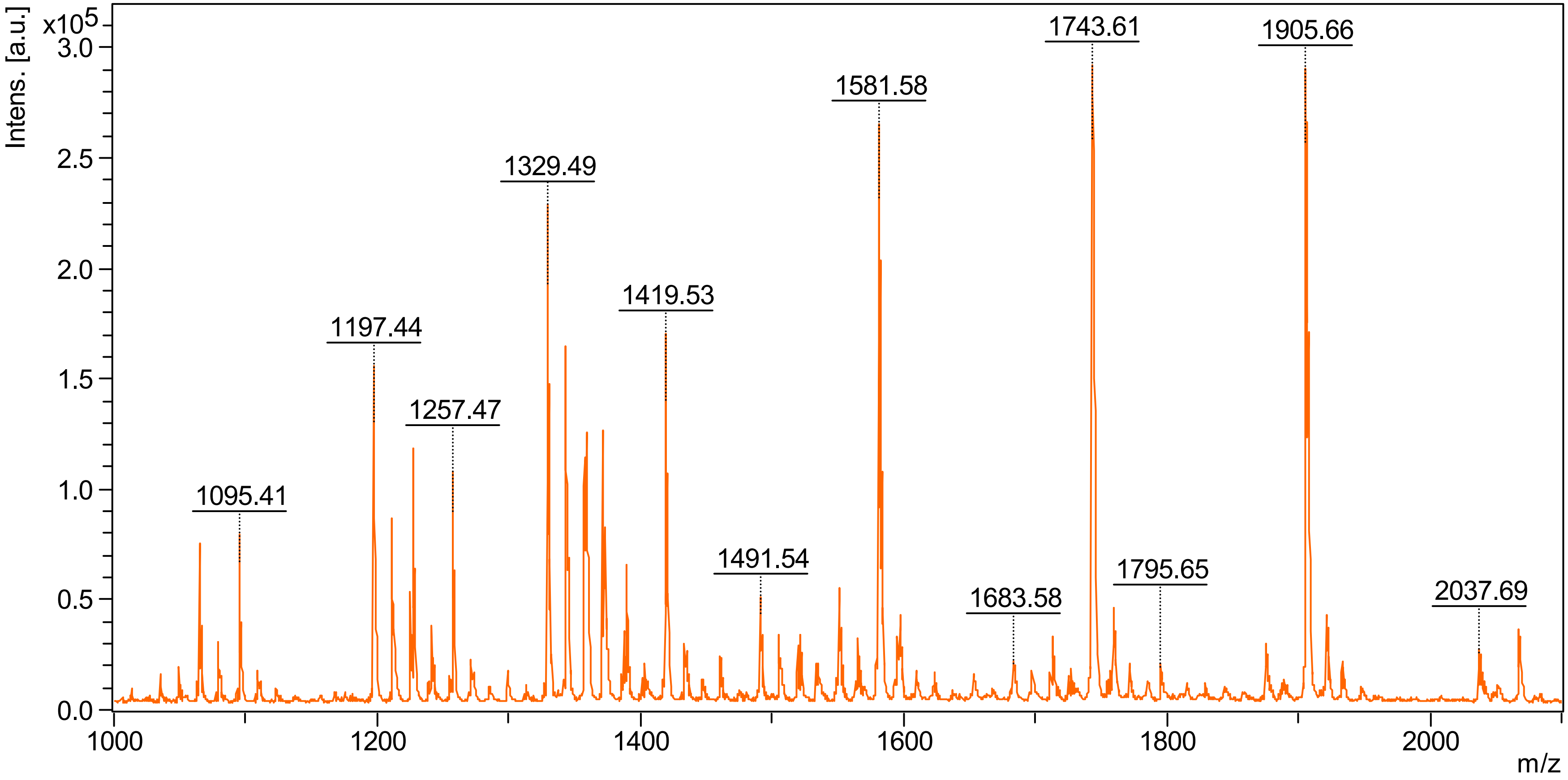

Vitagreen C.V. Protect 69 von Alge 49

Jar

Jar C-2

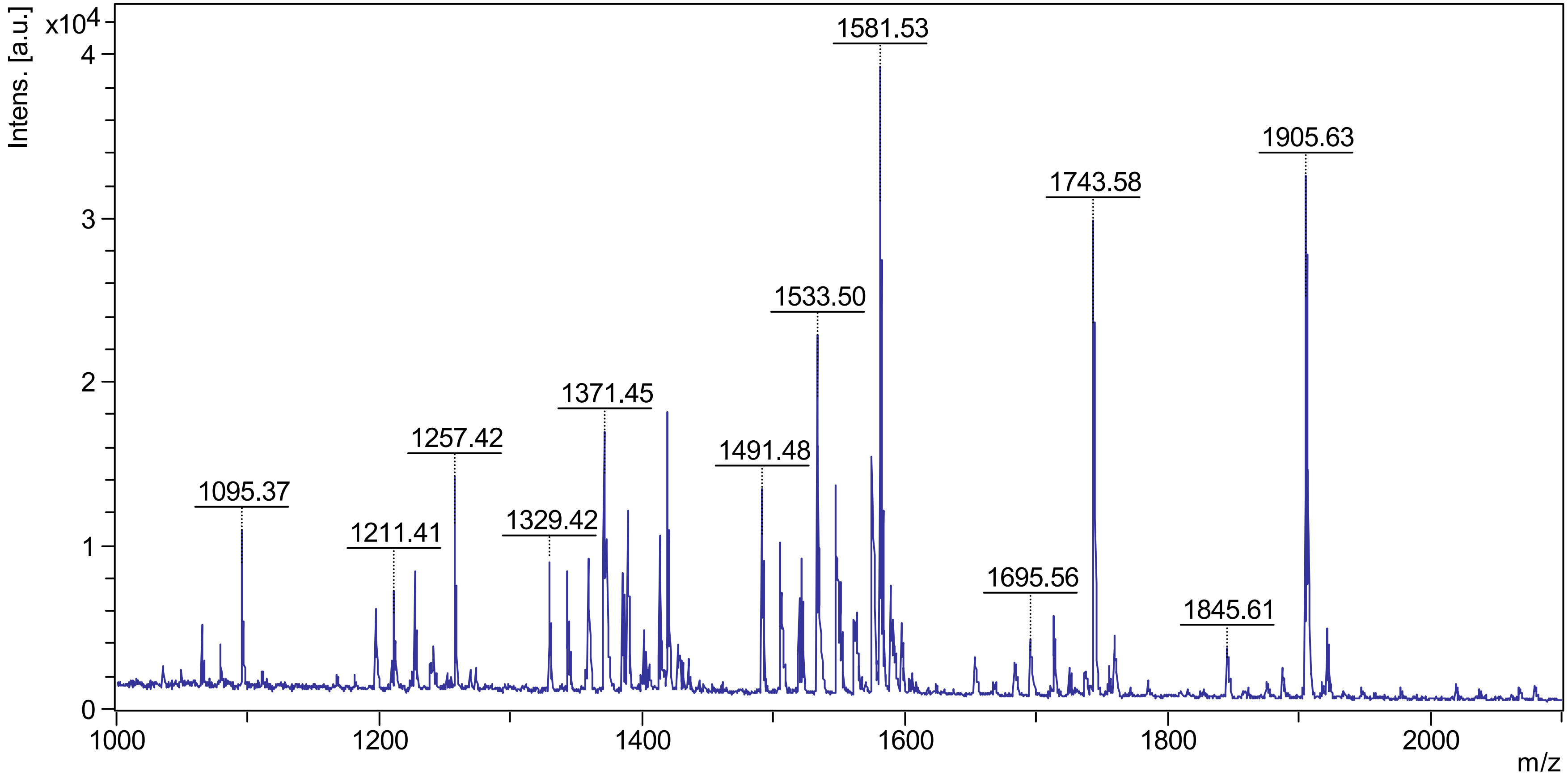

NutriCologg von Mai Alge 2

Jar

Jar C-7

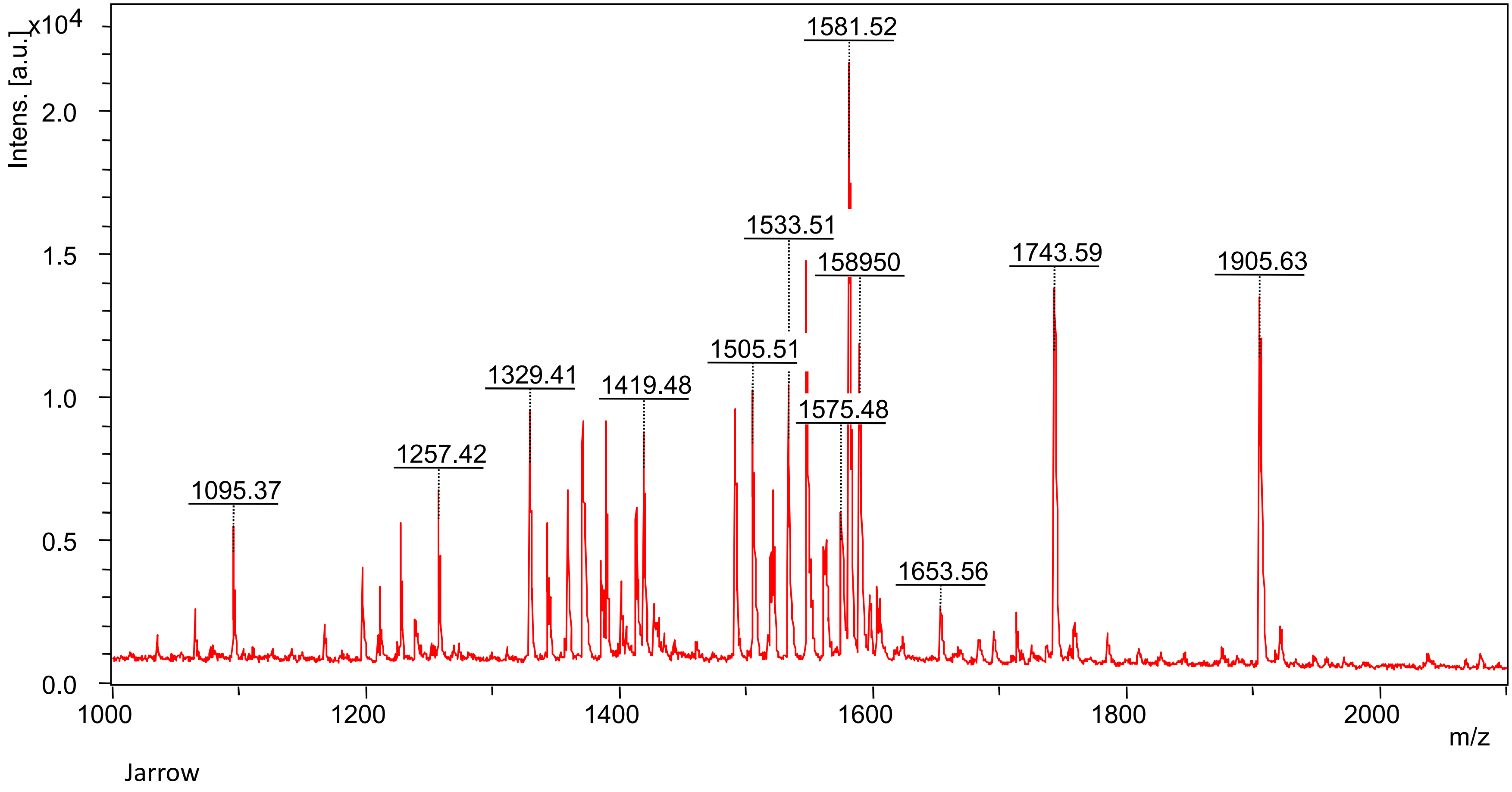
x10^4
Intens. [a.u.]
2.0
1.5
1.0
0.5
0.0
1581.52
1533.51
158950
1743.59
1905.63
1505.51
1329.41
1419.48
1575.48
1257.42
1095.37
1653.56
1000
1200
1400
1600
1800
2000
m/z
Jarrow

Jar

Jar C-34

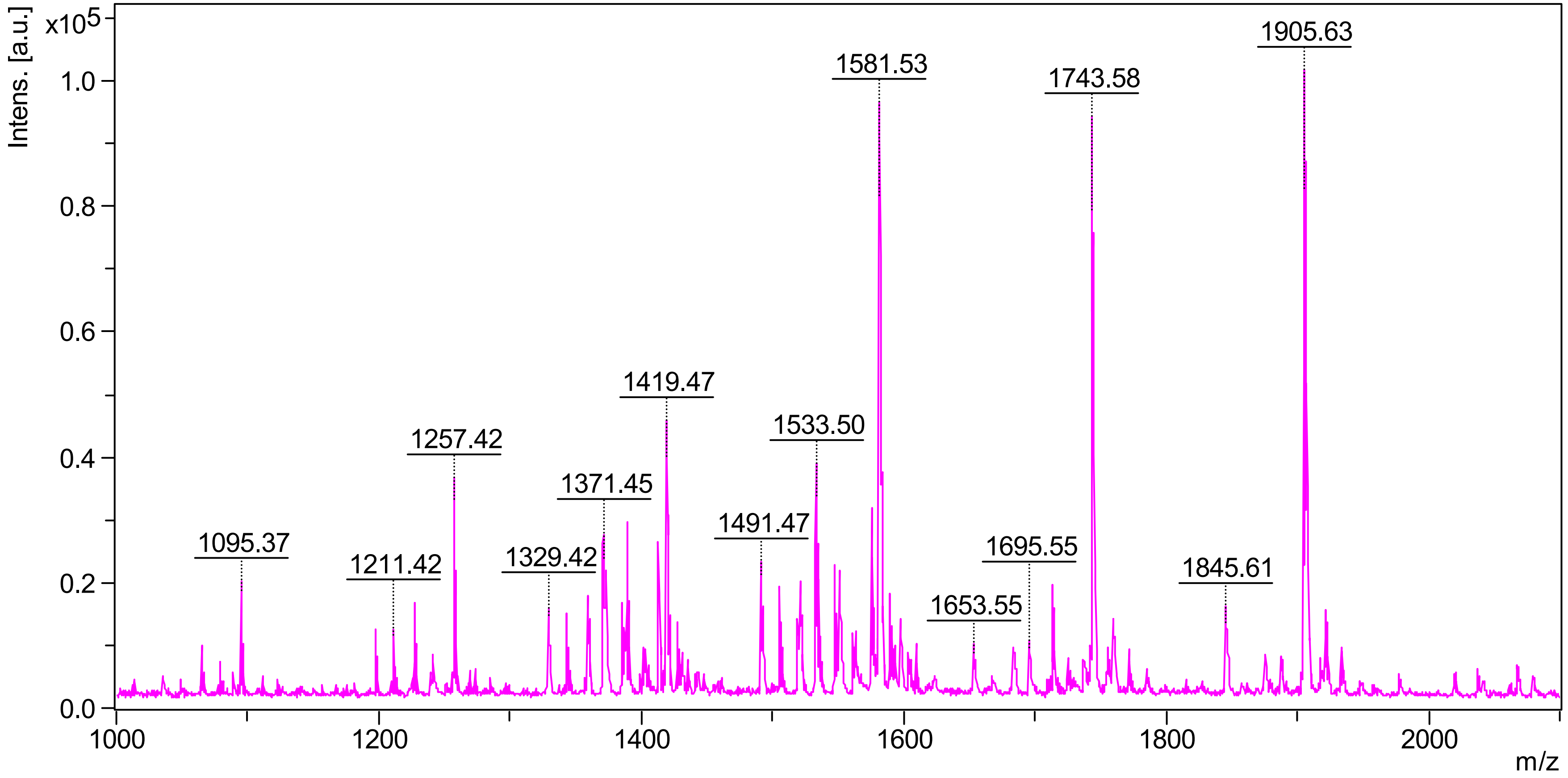

Alge 85 Feine Alge

Jar

Jar C-20

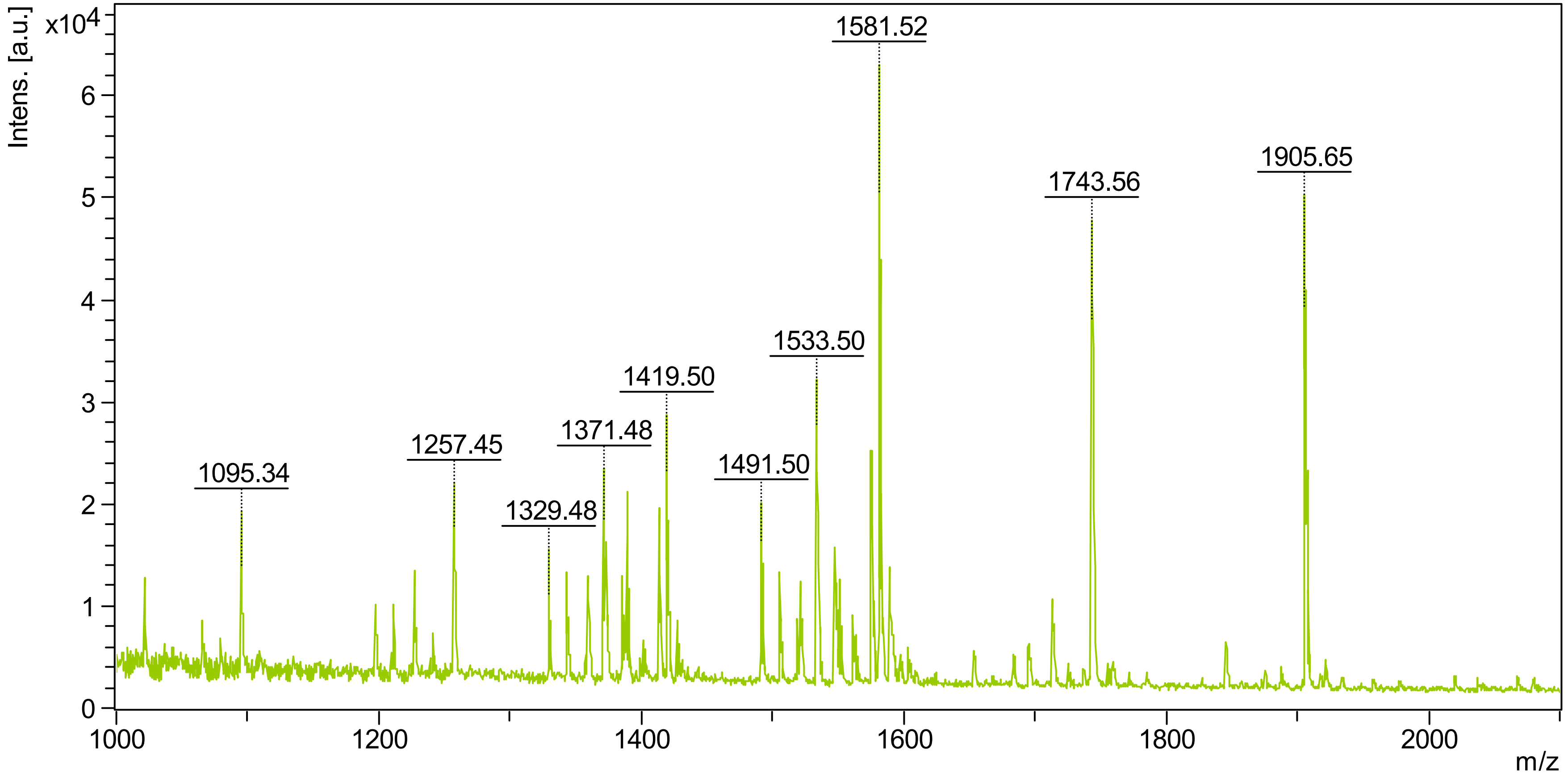

A Heide pyr

Jar

Jar C-45

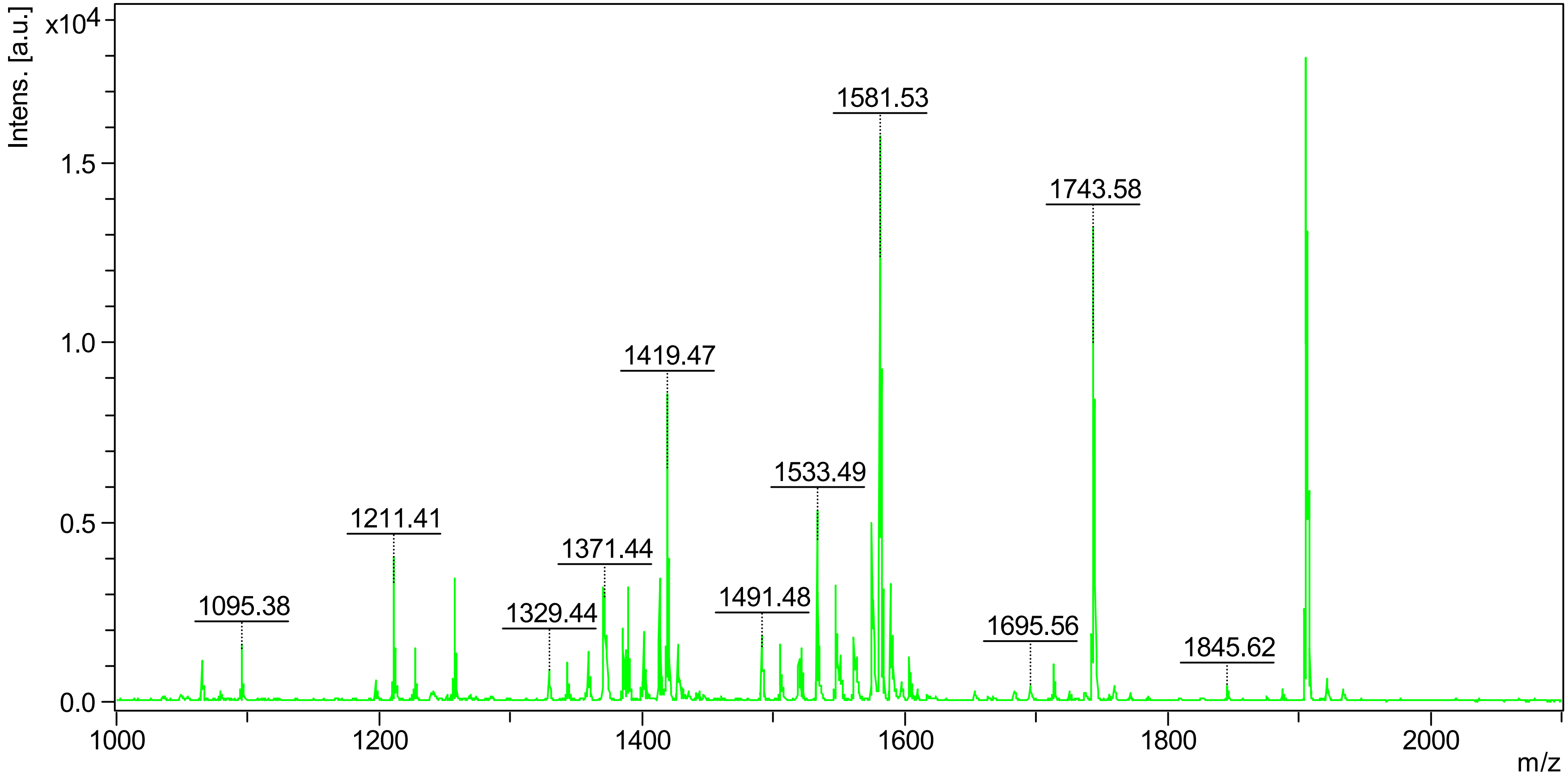

20180405 RF Alge MyProtein

Jar

Jar C-51

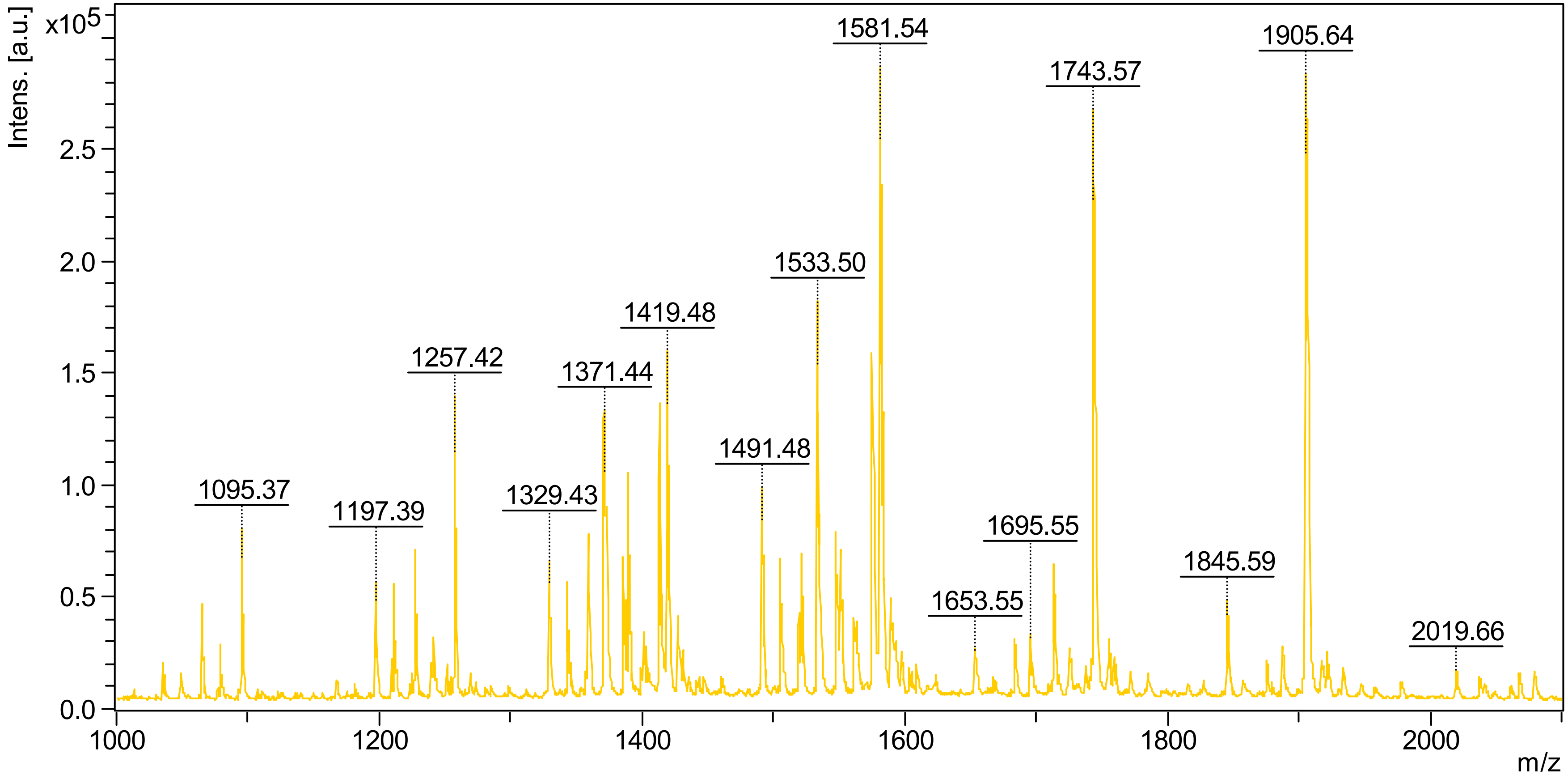

Warnke 51 von Alge 31

Jar

Jar C-52

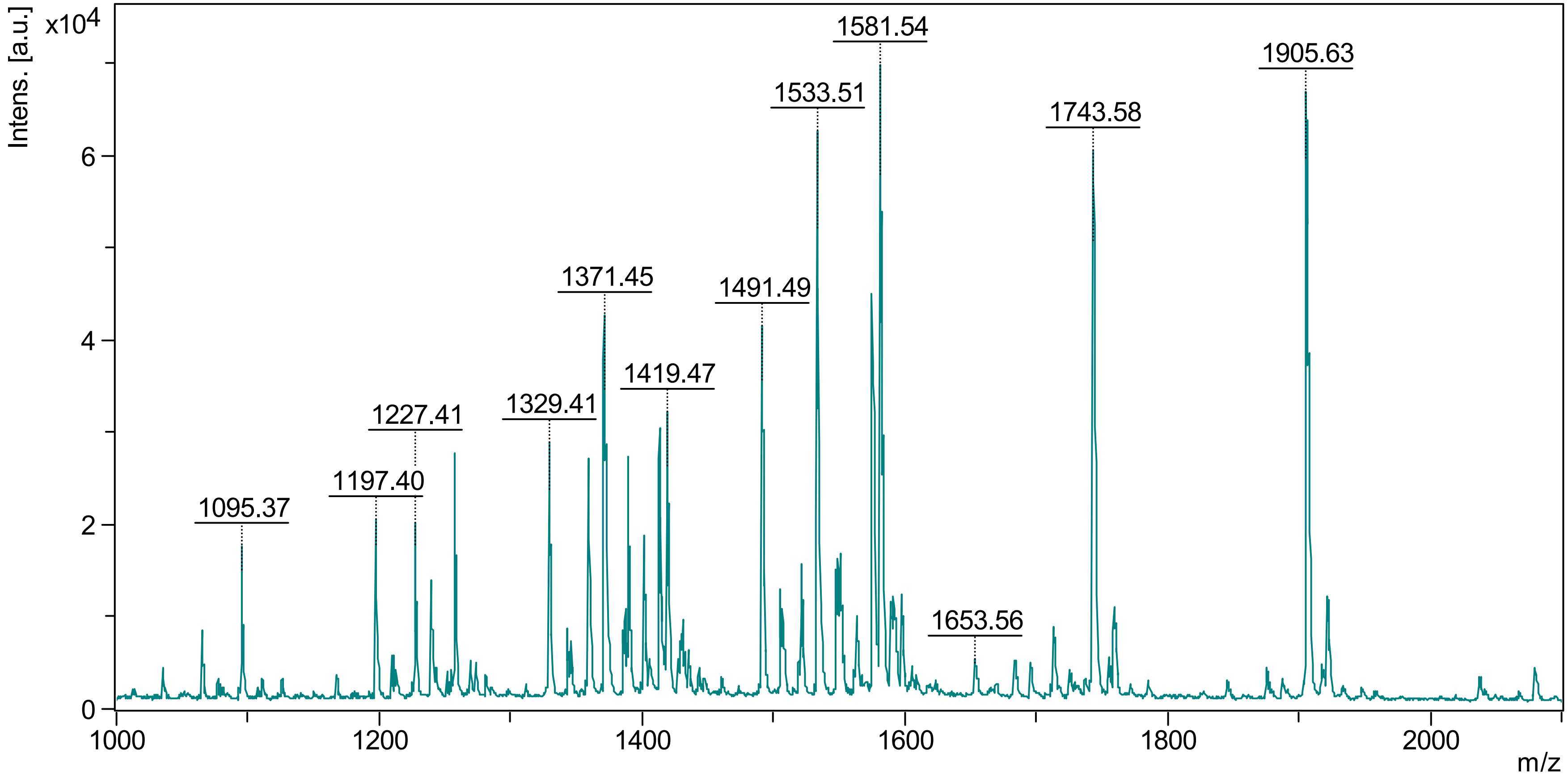

green valley

Jar

Jar C-58

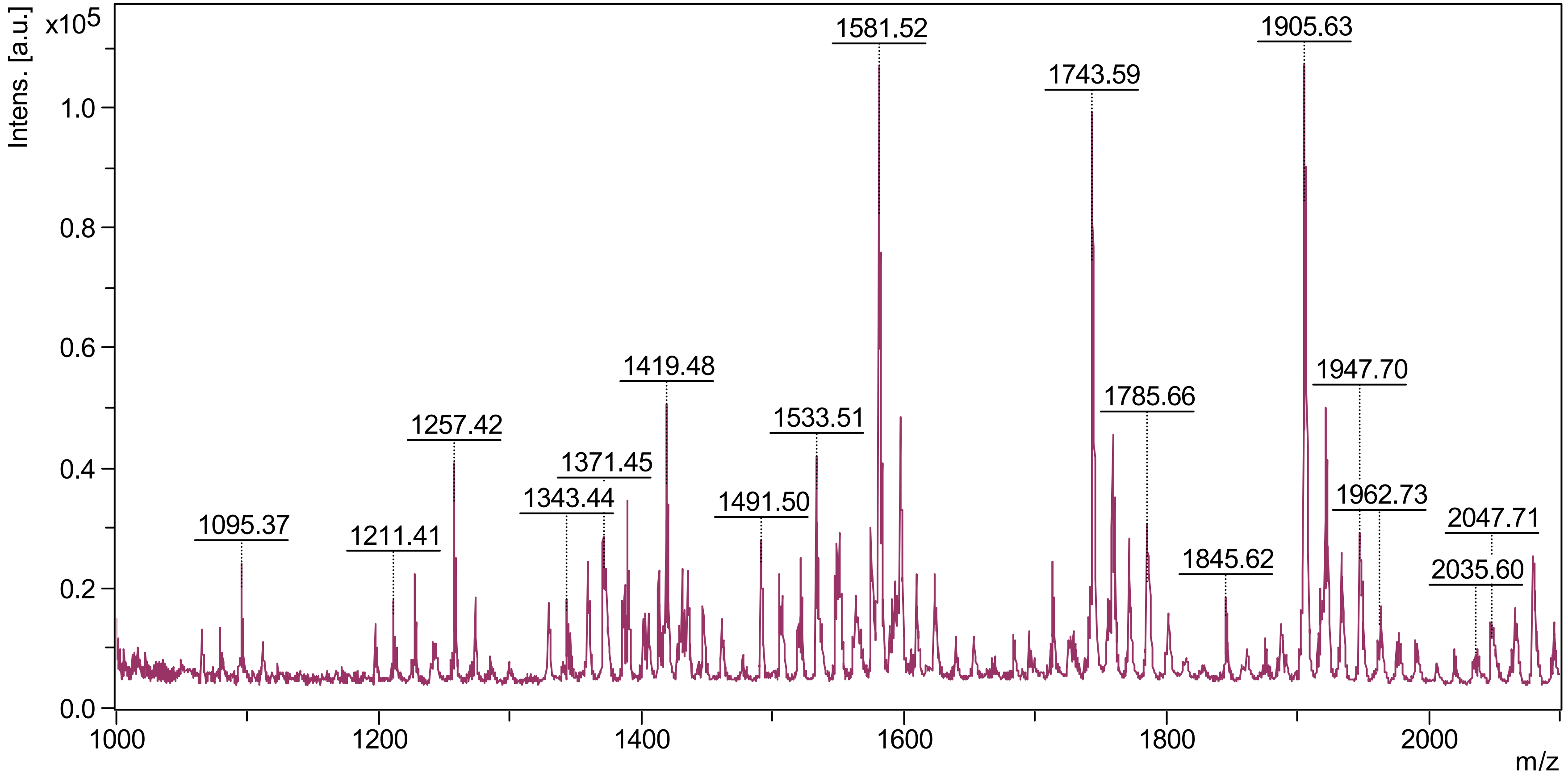

Vita World 58 von Mai Alge 38

Jar

Jar C-61

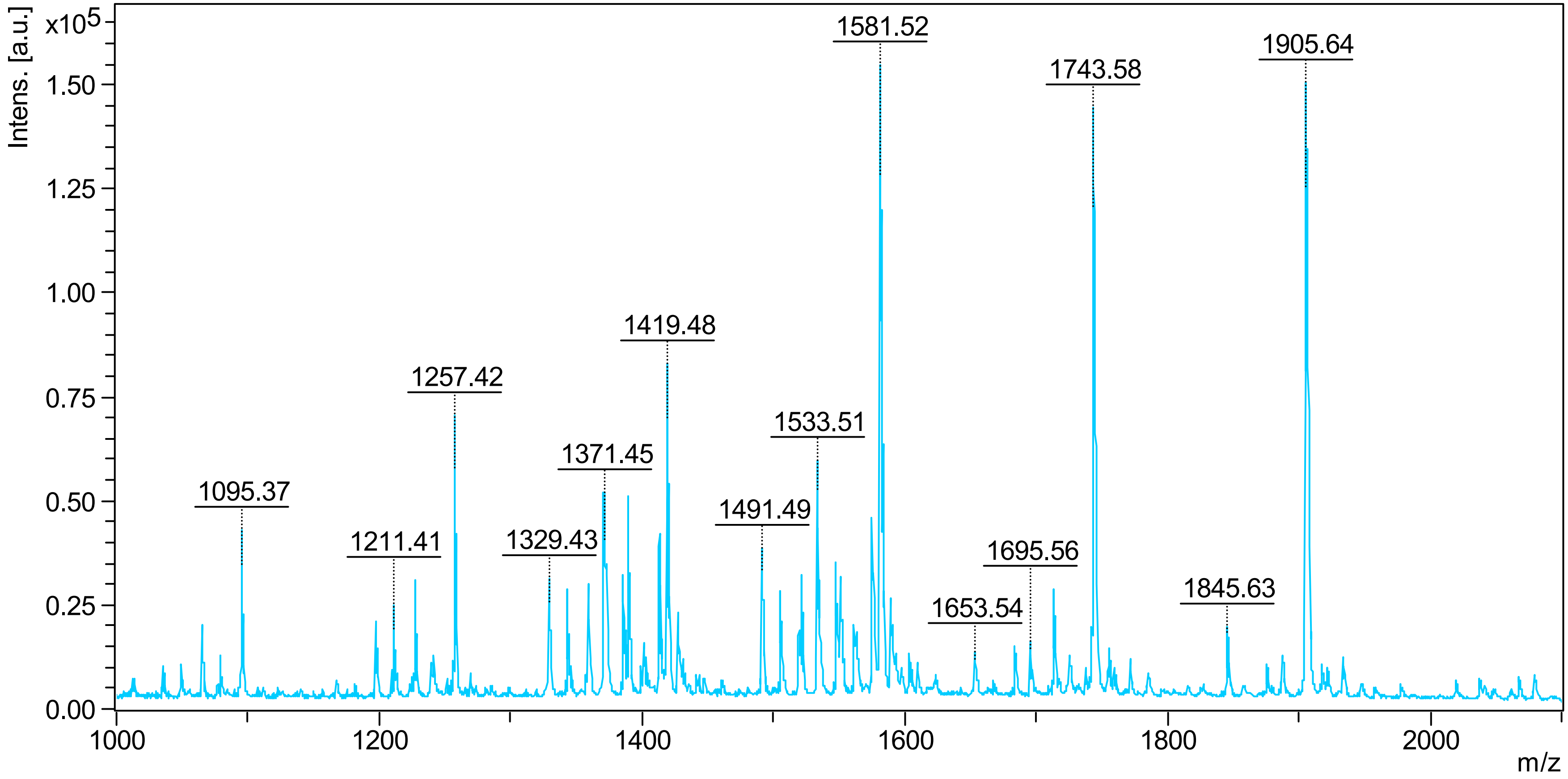

Aktiv organic 61 von 41

Jar

Jar C-66

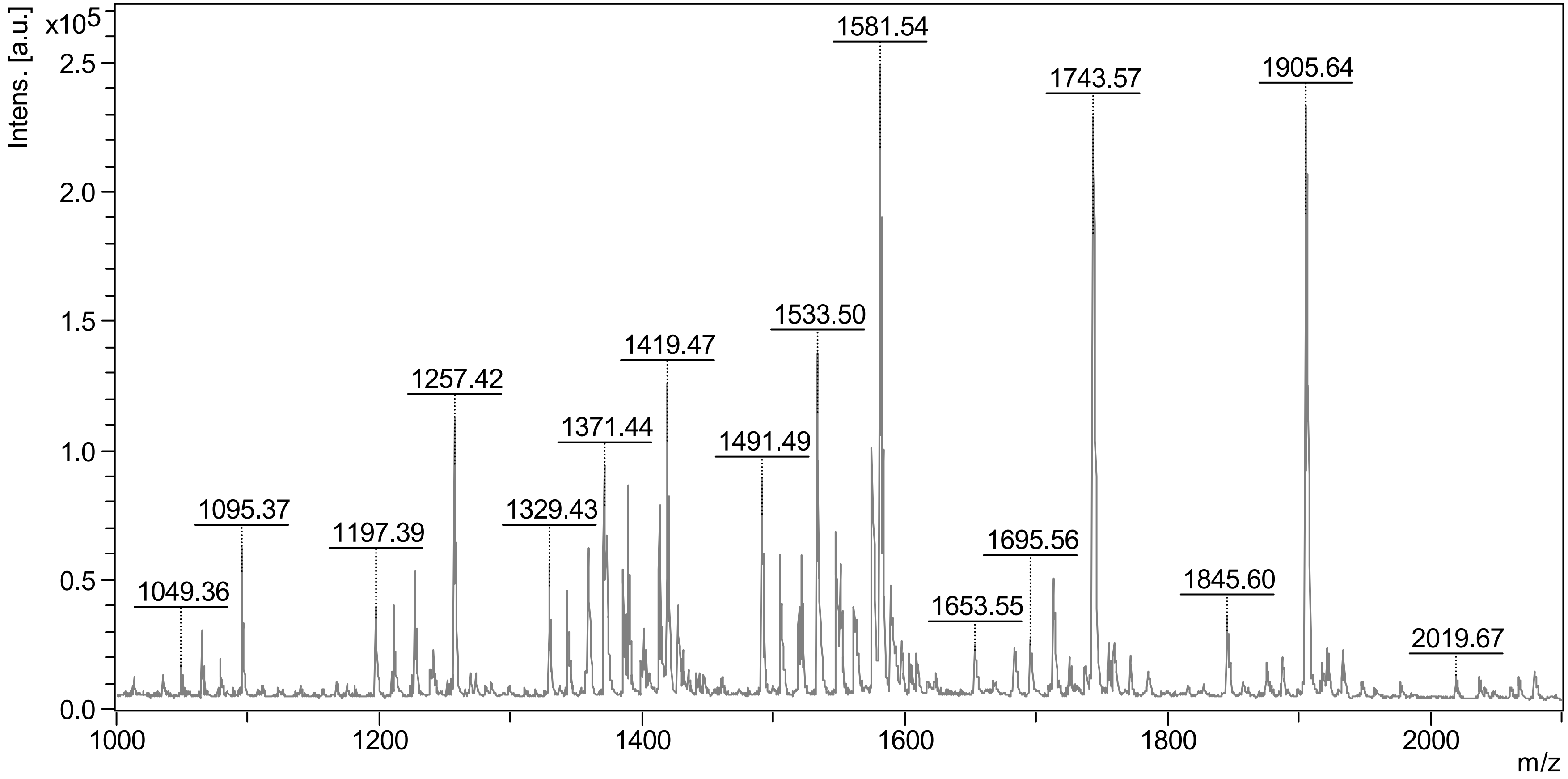

Biokin 66 von 46

Jar

Jar C-72

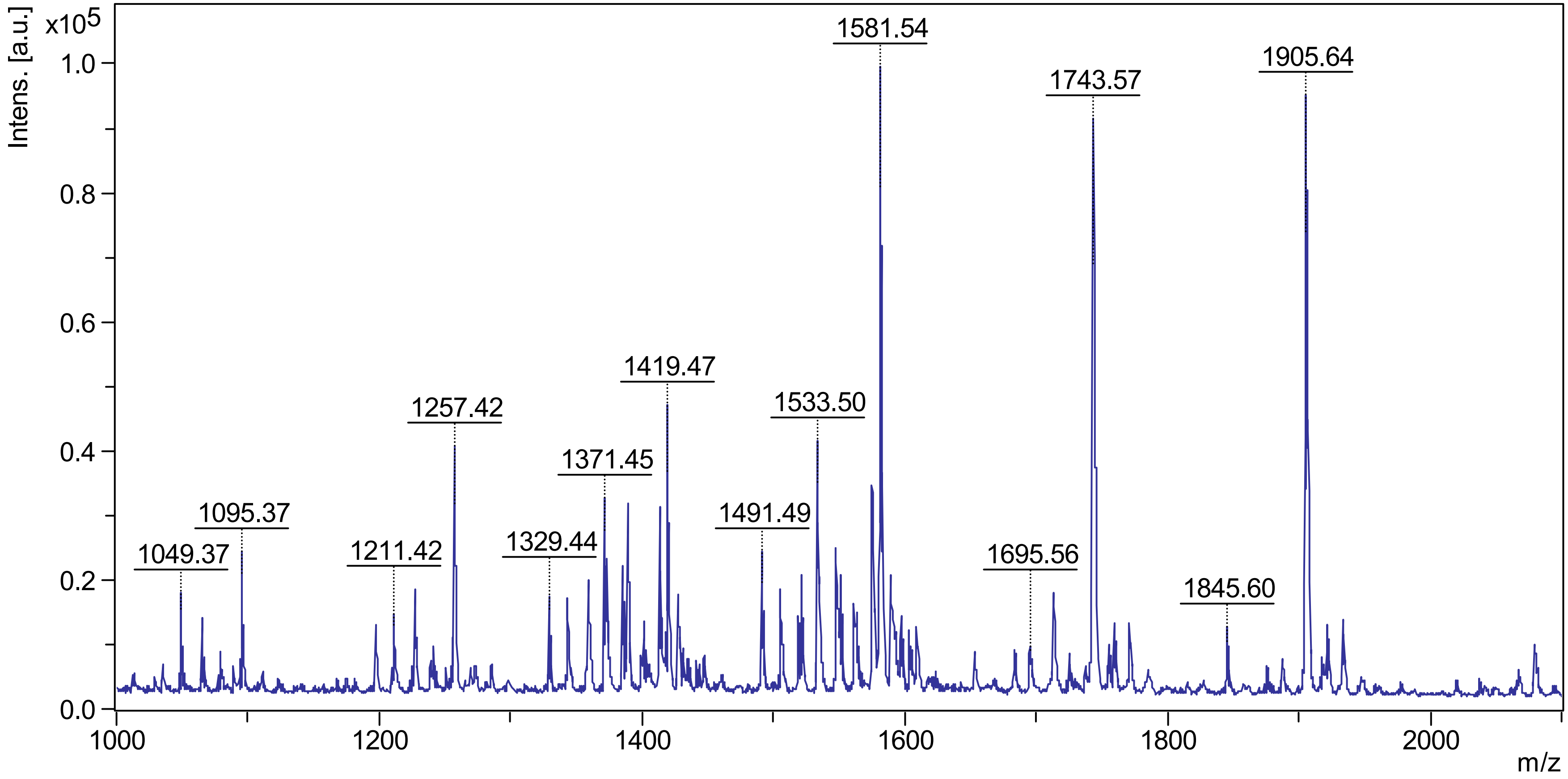

Healthy tree von Alge 72

Jar

Jar C-75

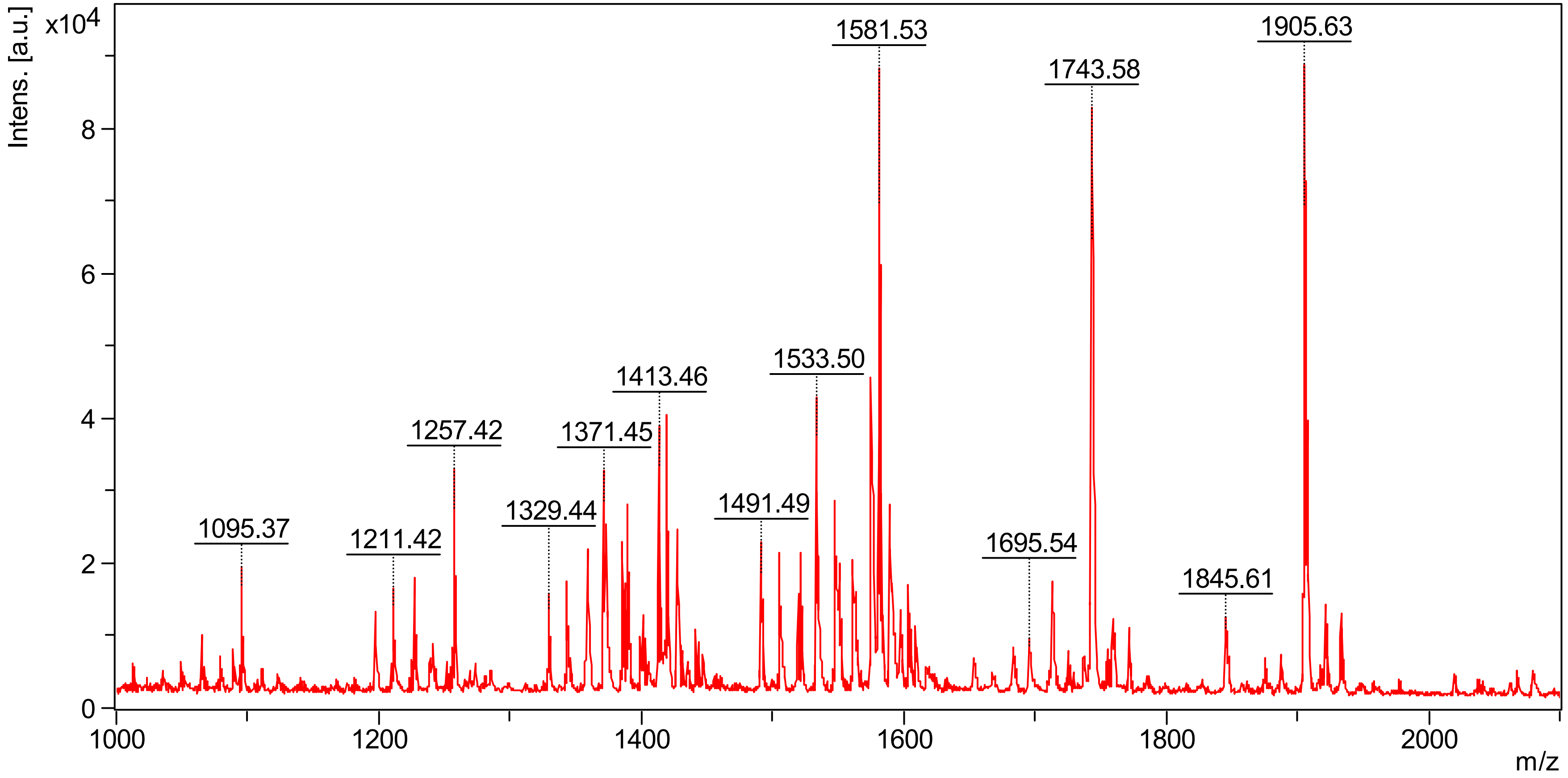

Nutri Superfood Alge 75

Jar

Jar C-78

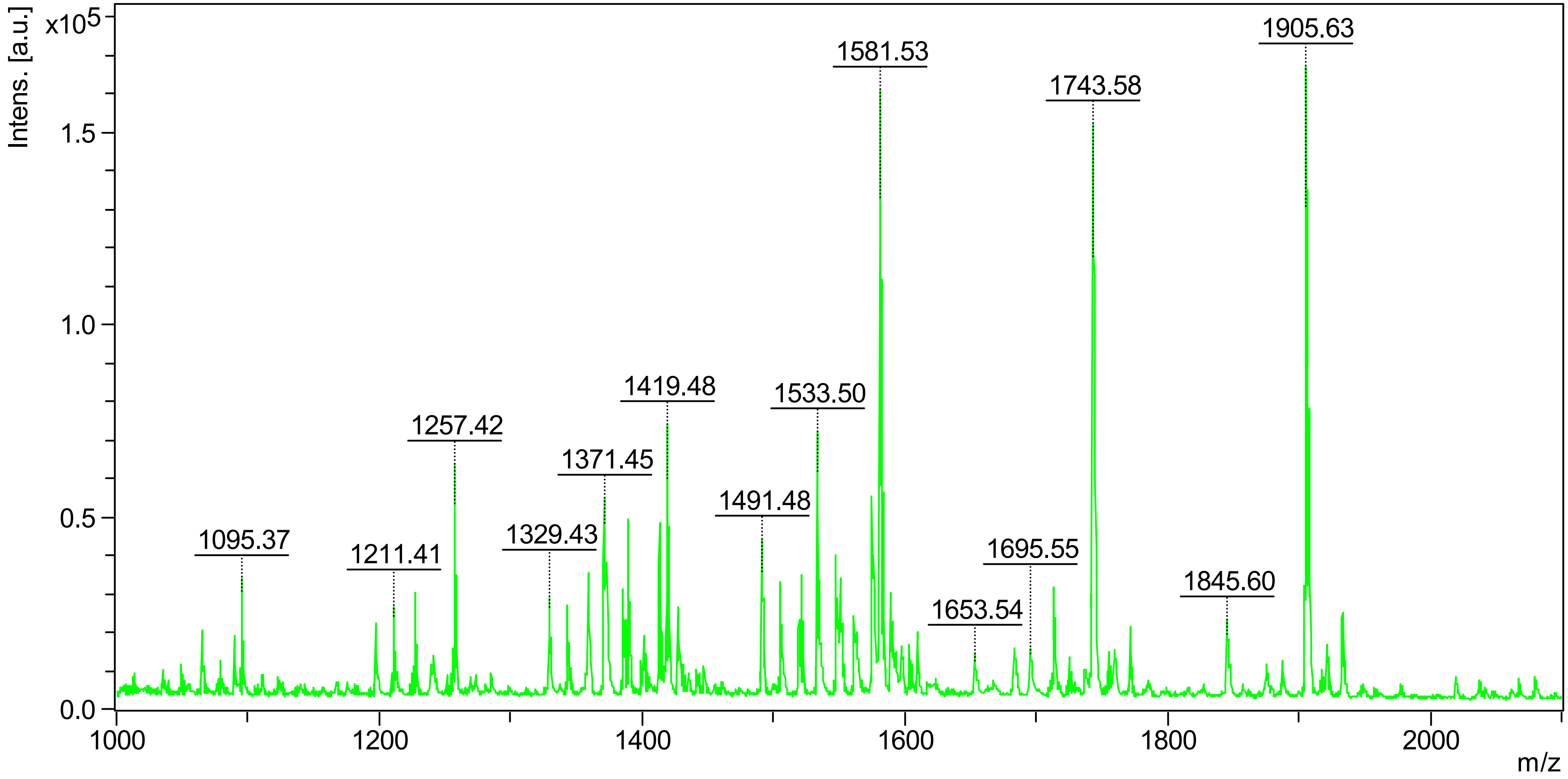

Sevenhills von Alge 78

Gov

Gov C-13

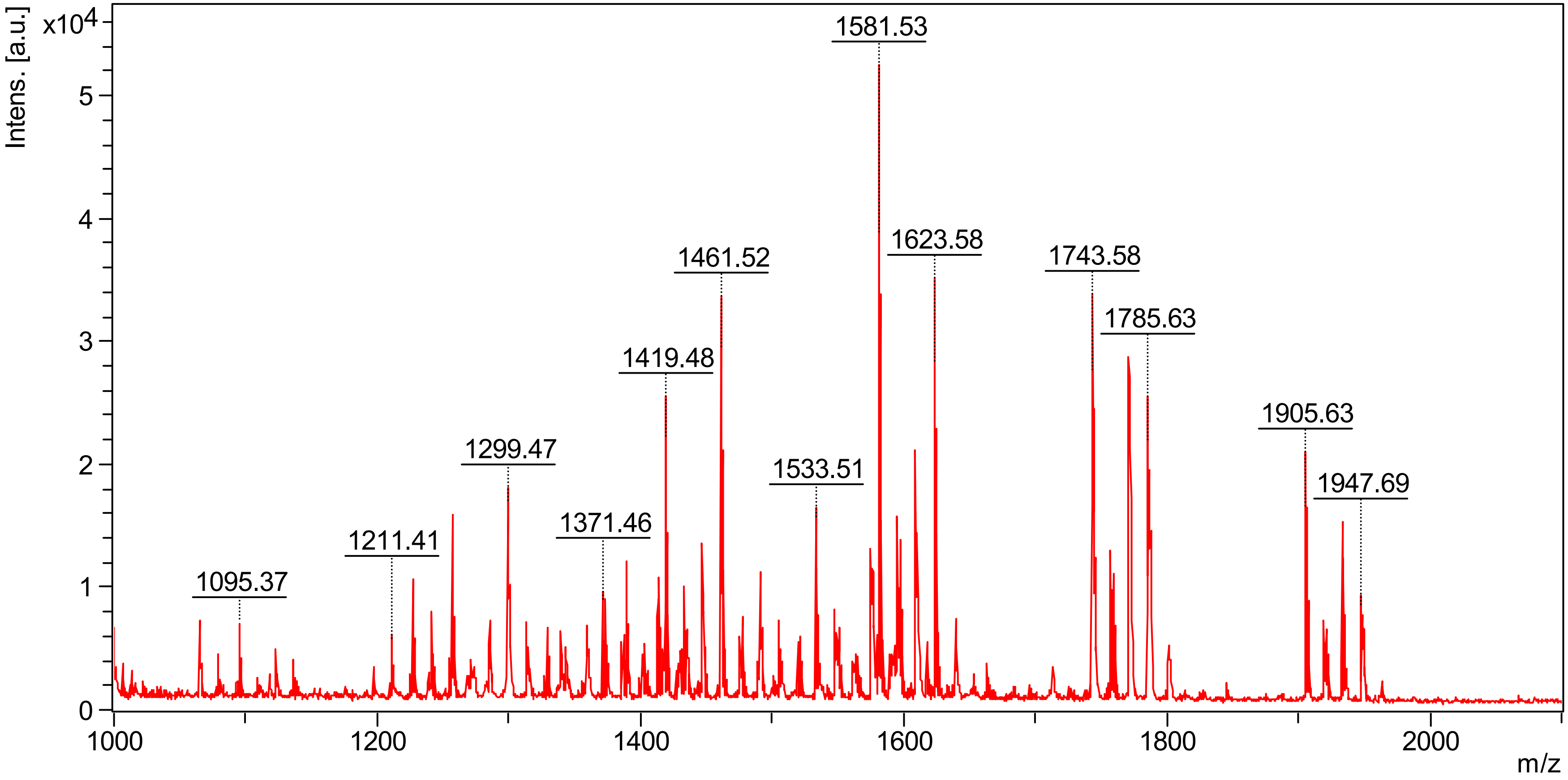

RM 20160914 C_pyr Rainforest cc

Gov

Gov C-30

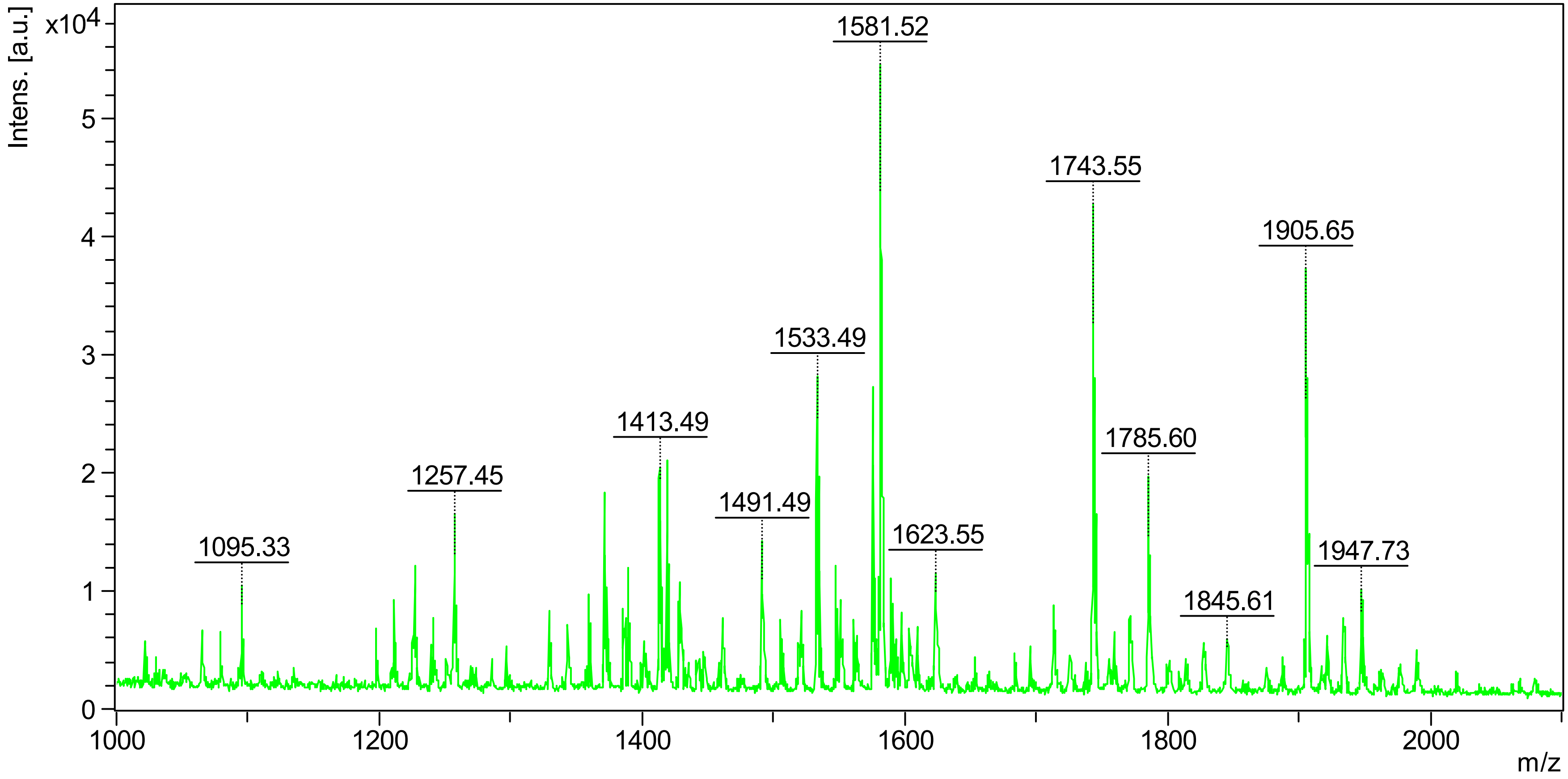

RM_170914 Dragonspice

Gov

Gov C-35

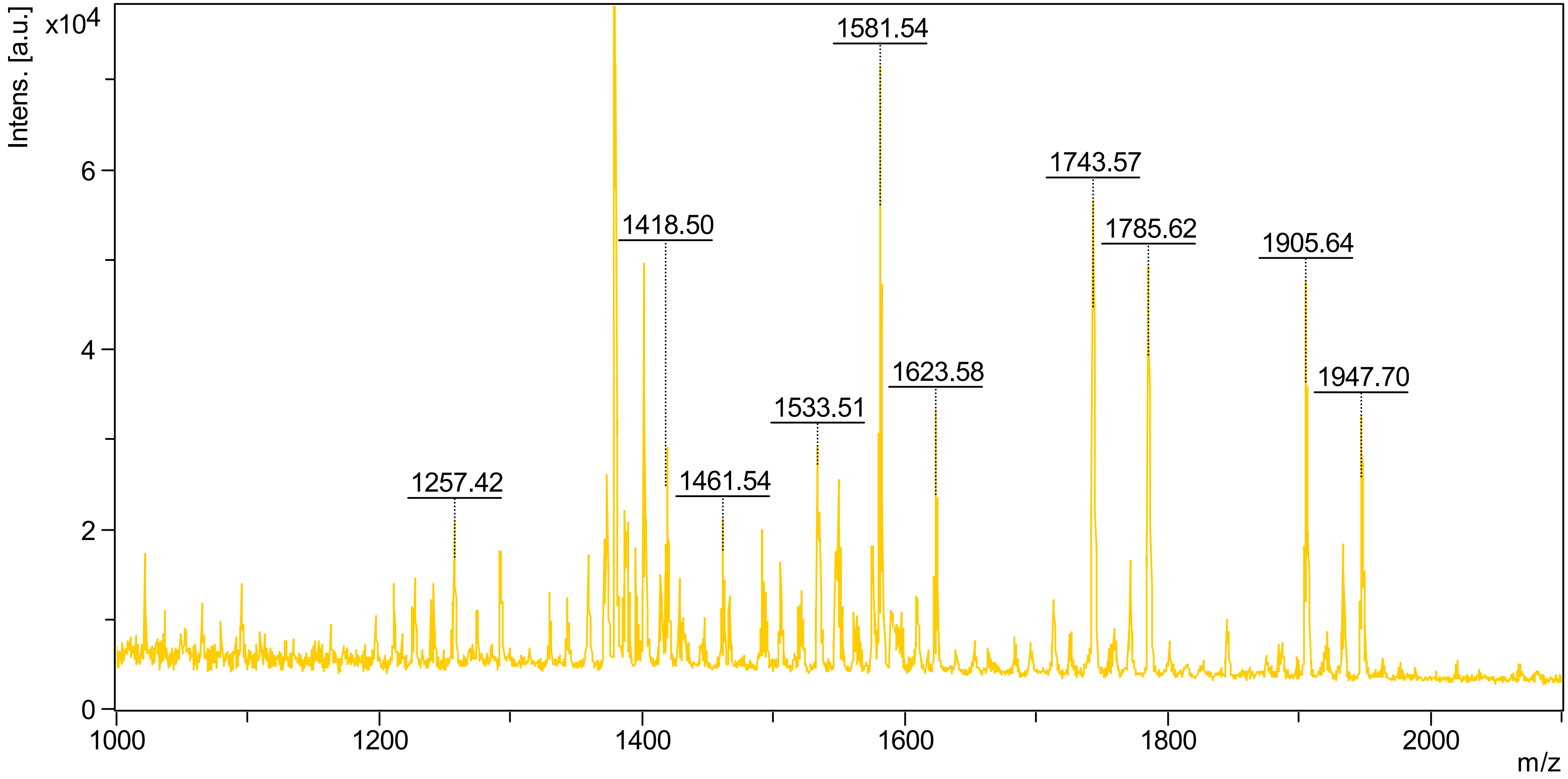

NU3

Gov

Gov C-37

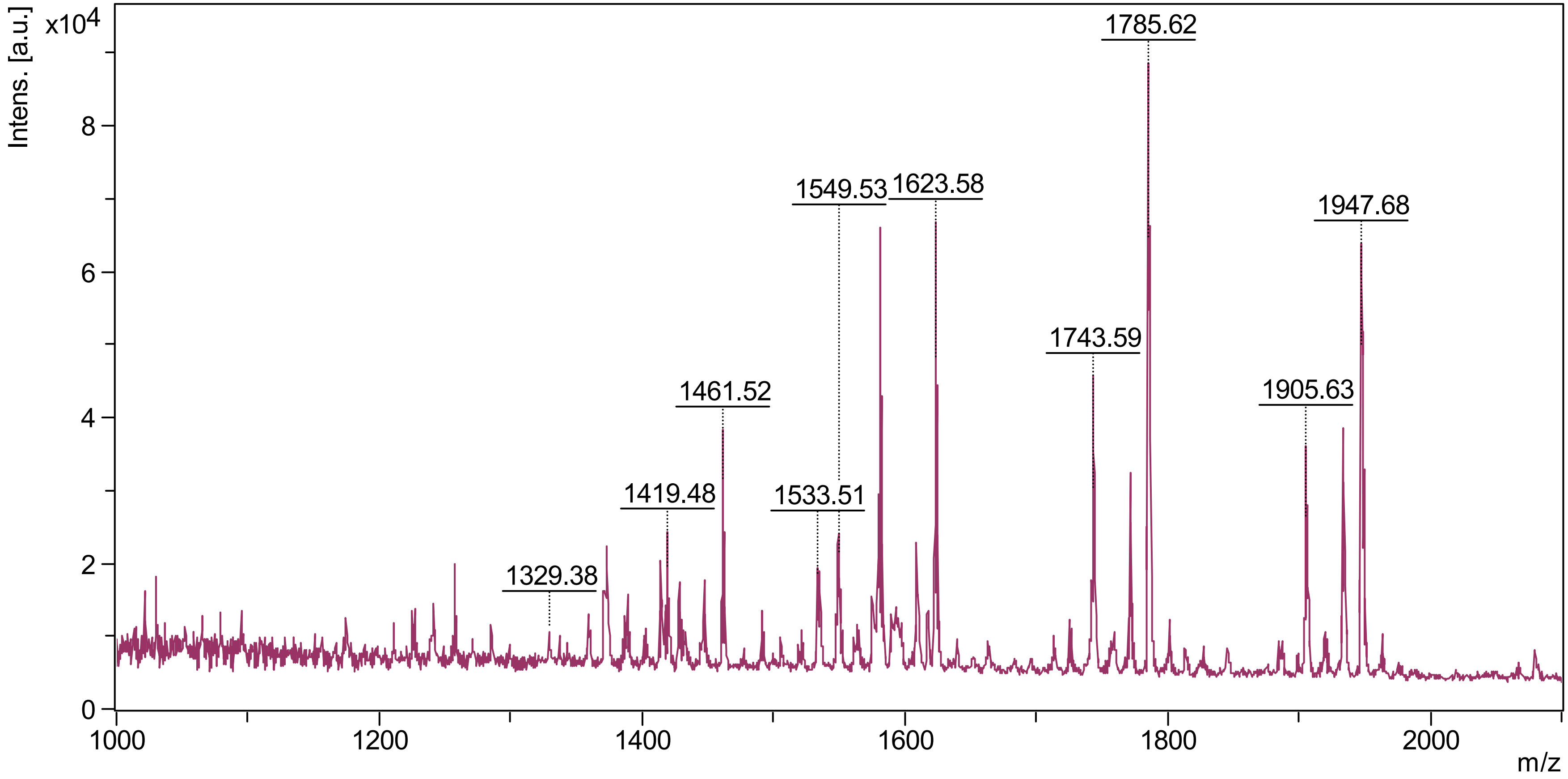

Wellnest (= Wellnest 171011)

Gov

Gov C-42

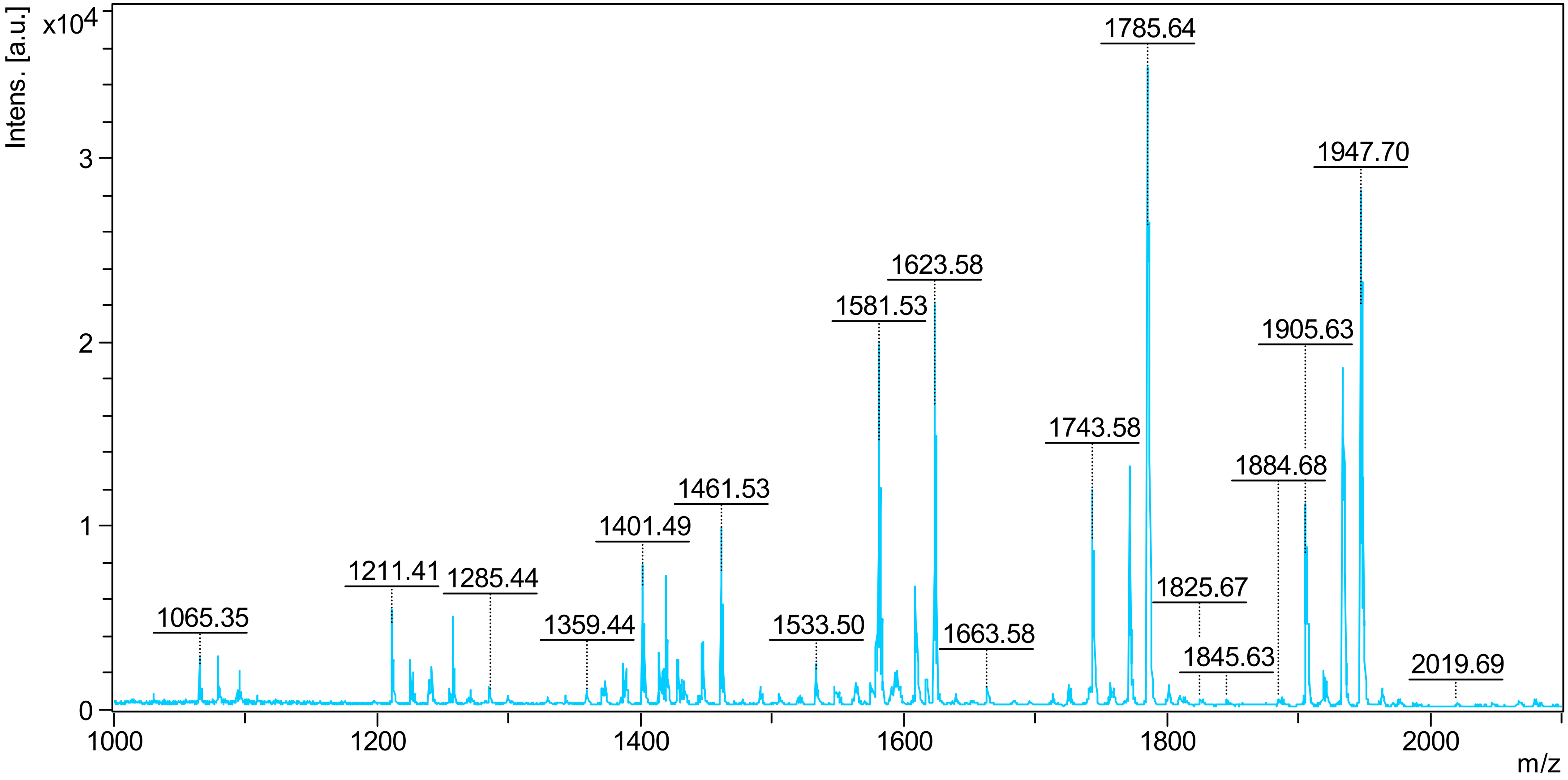

180328 TopNature

Gov

Gov C-43

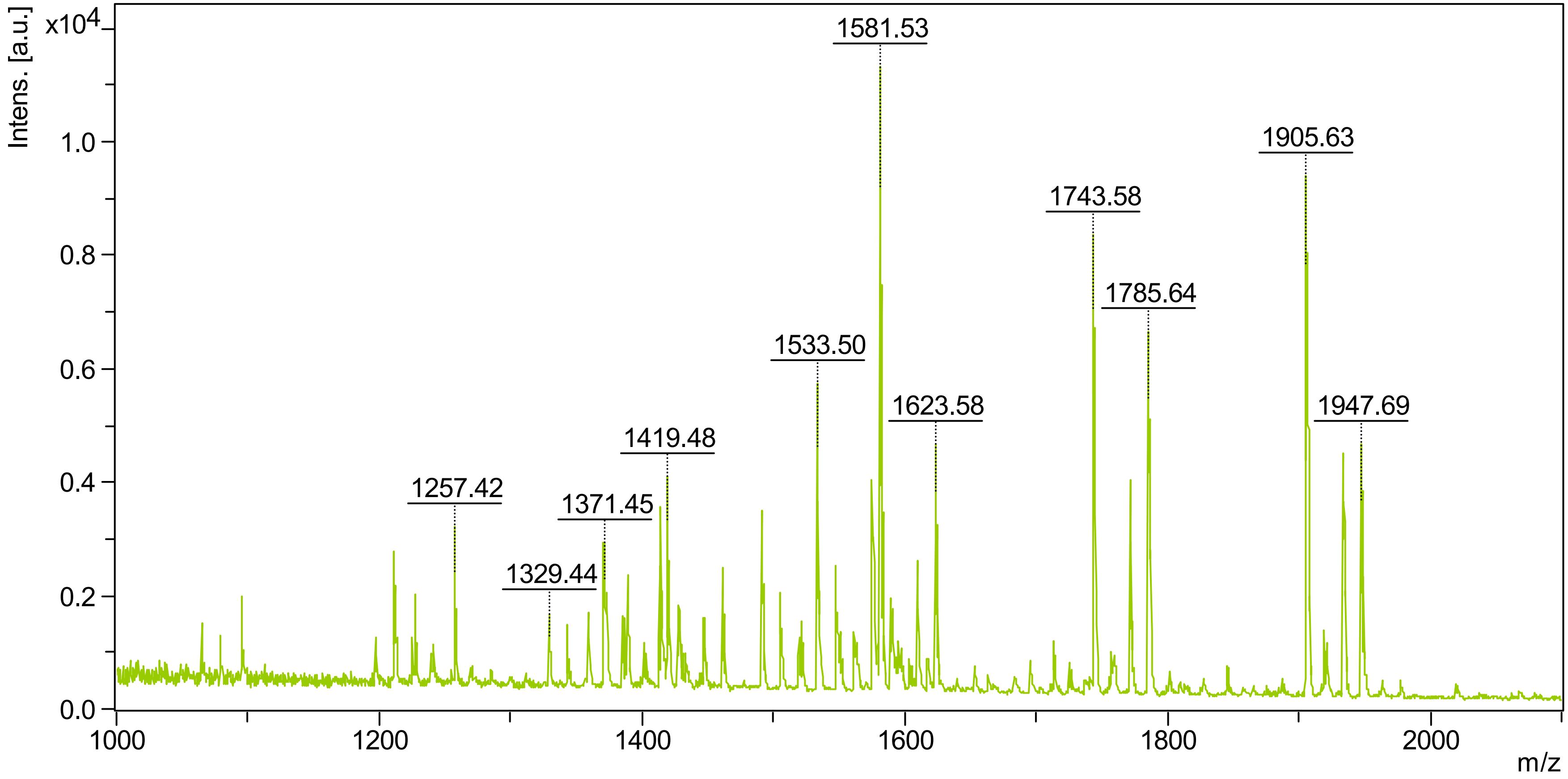

180328_hanoju

Gov

Gov C-44

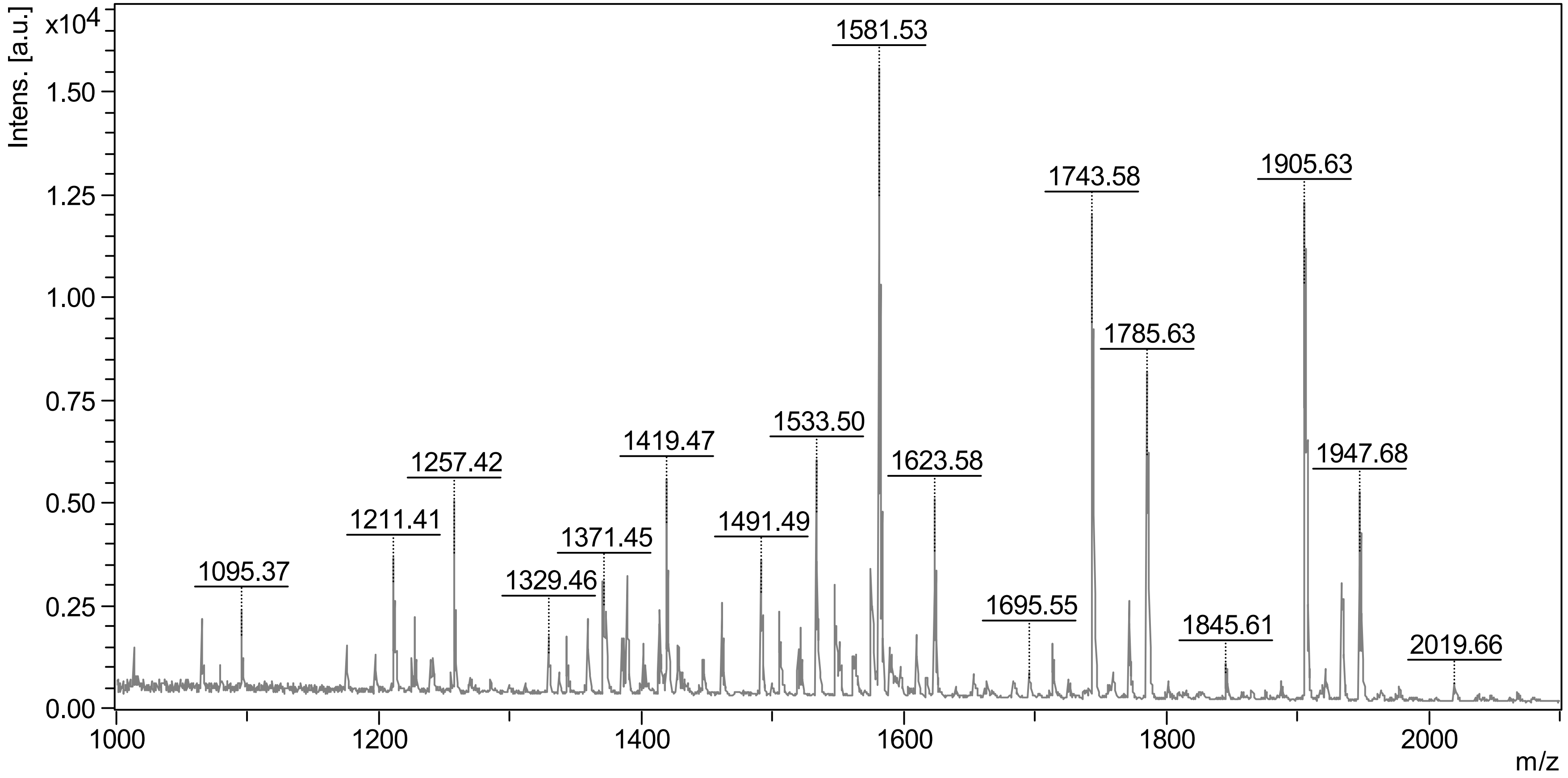

180328_govindal

Gov

Gov C-50

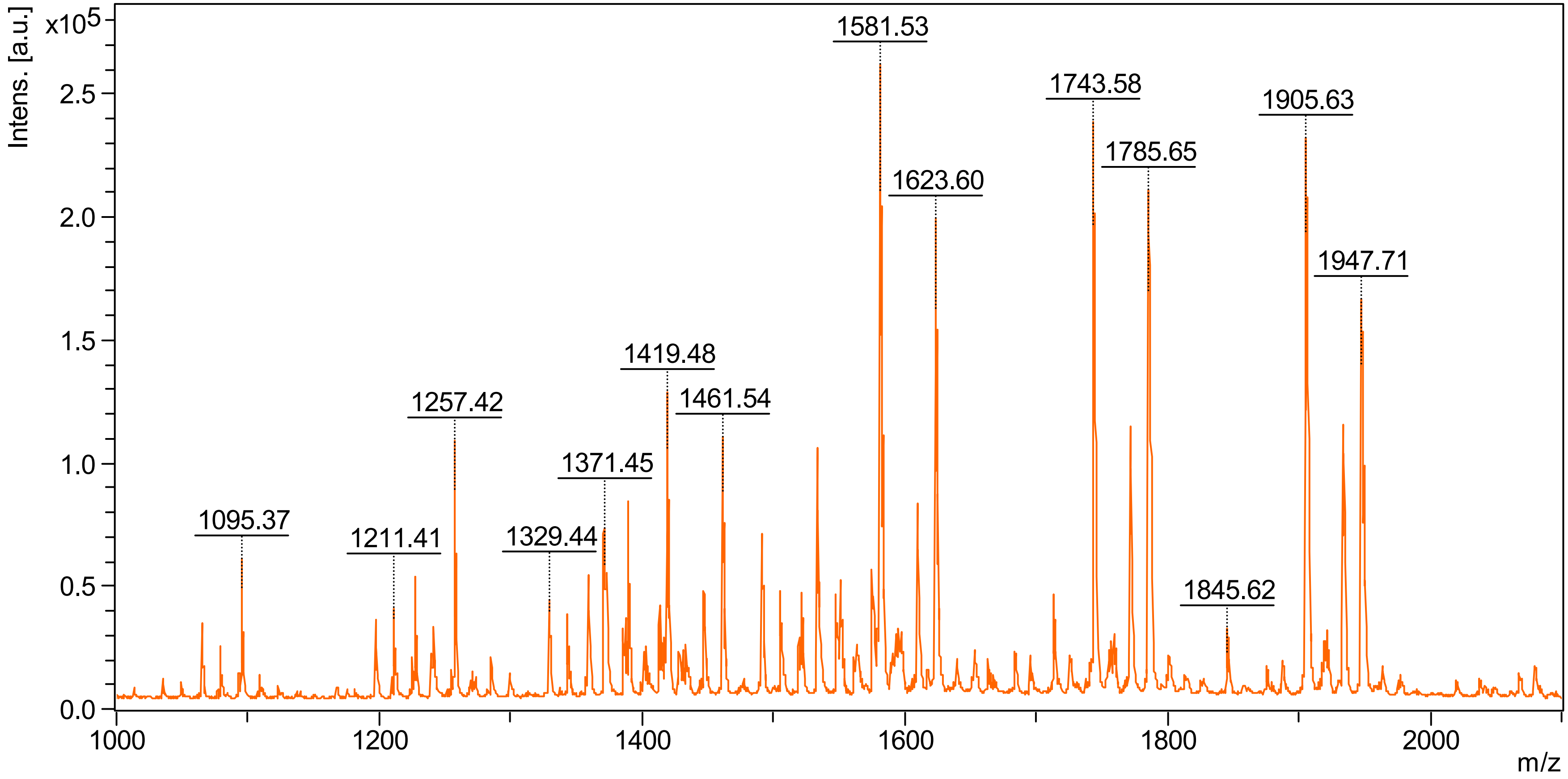

Alge 56a von 36a

Gov

Gov C-56

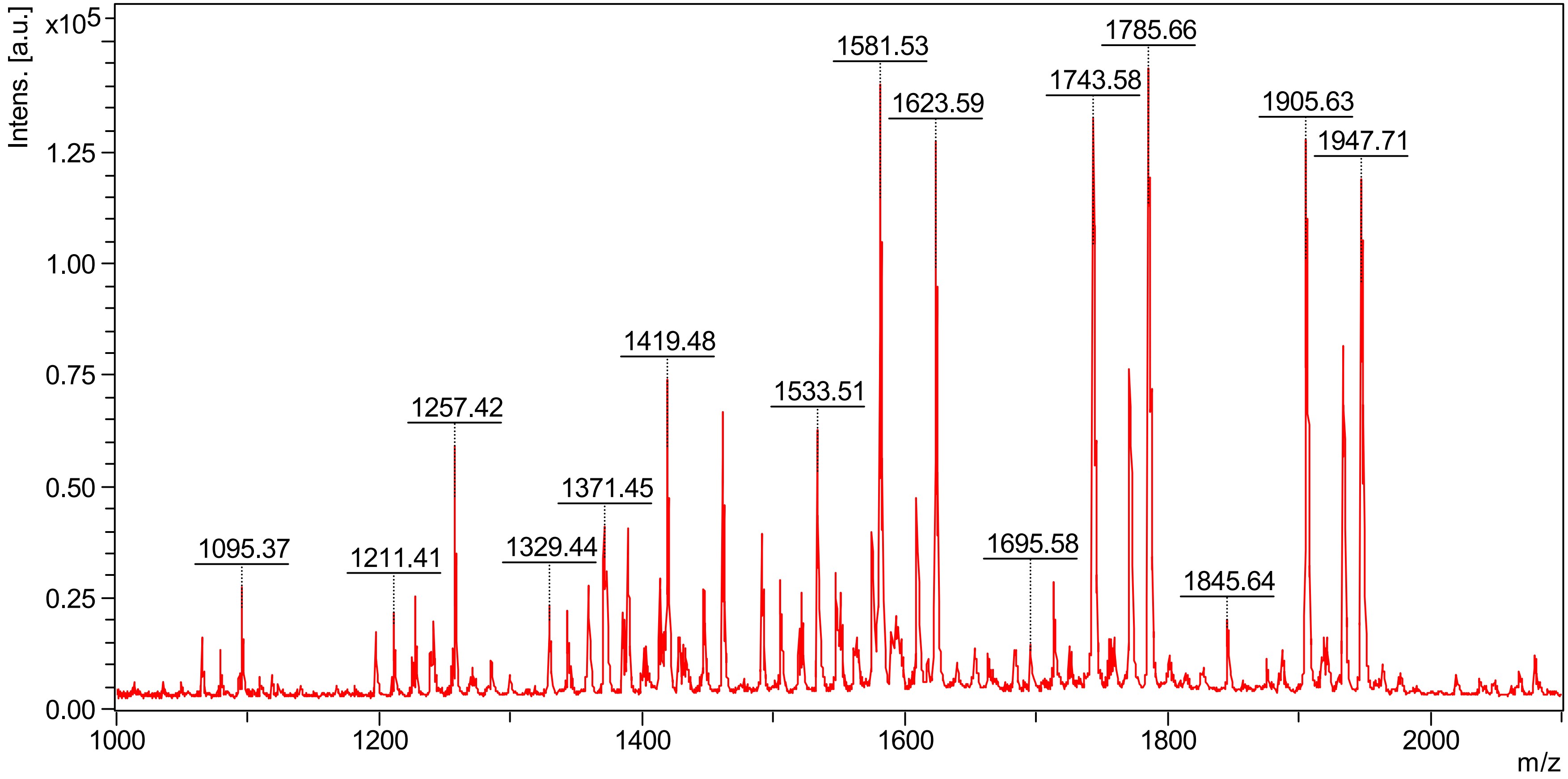

Alge 56b von 36b

Gov

Gov C-62

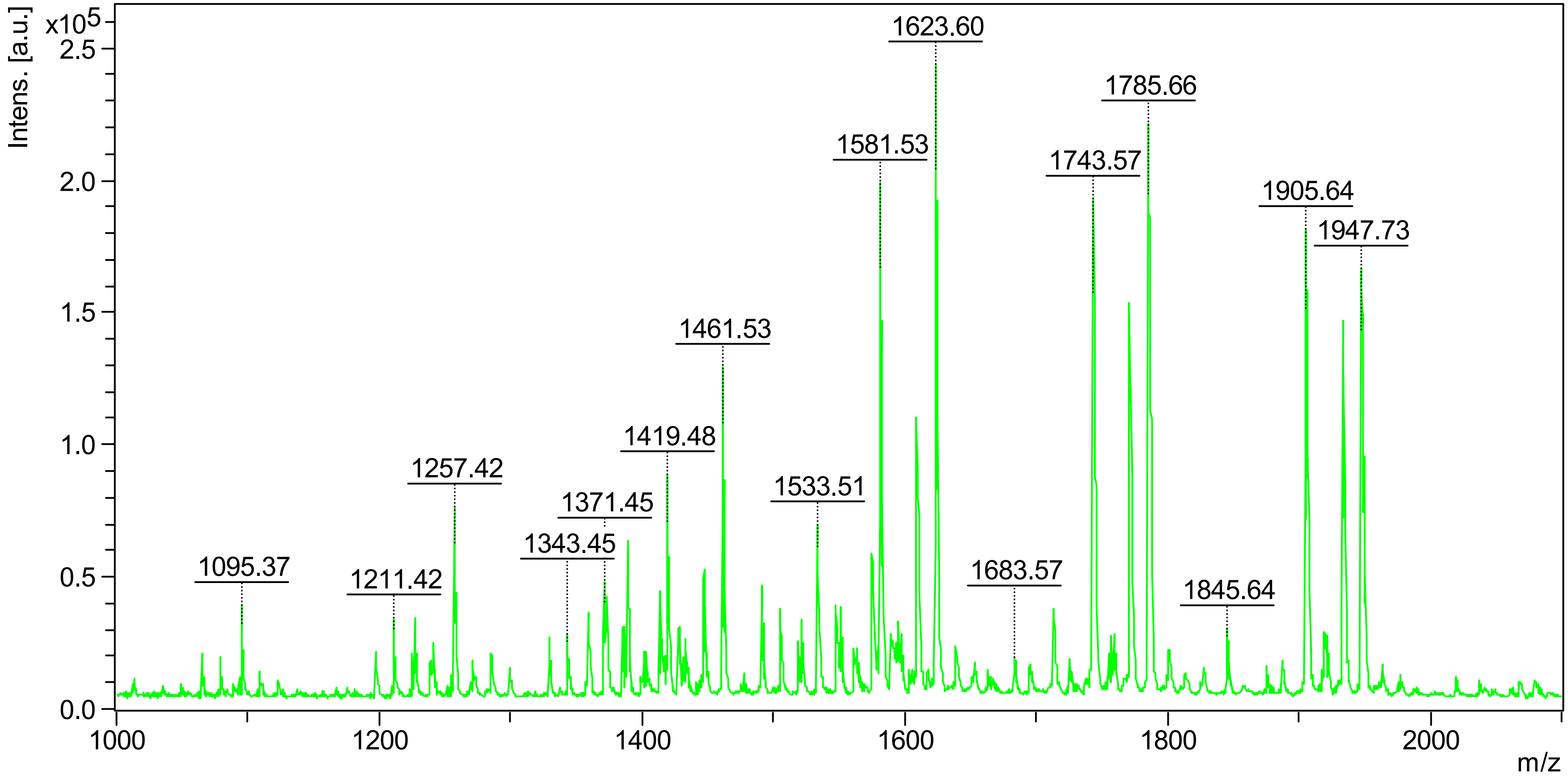

RM 20160914 C_pyr Rainforest cc

Asp

Asp C-25

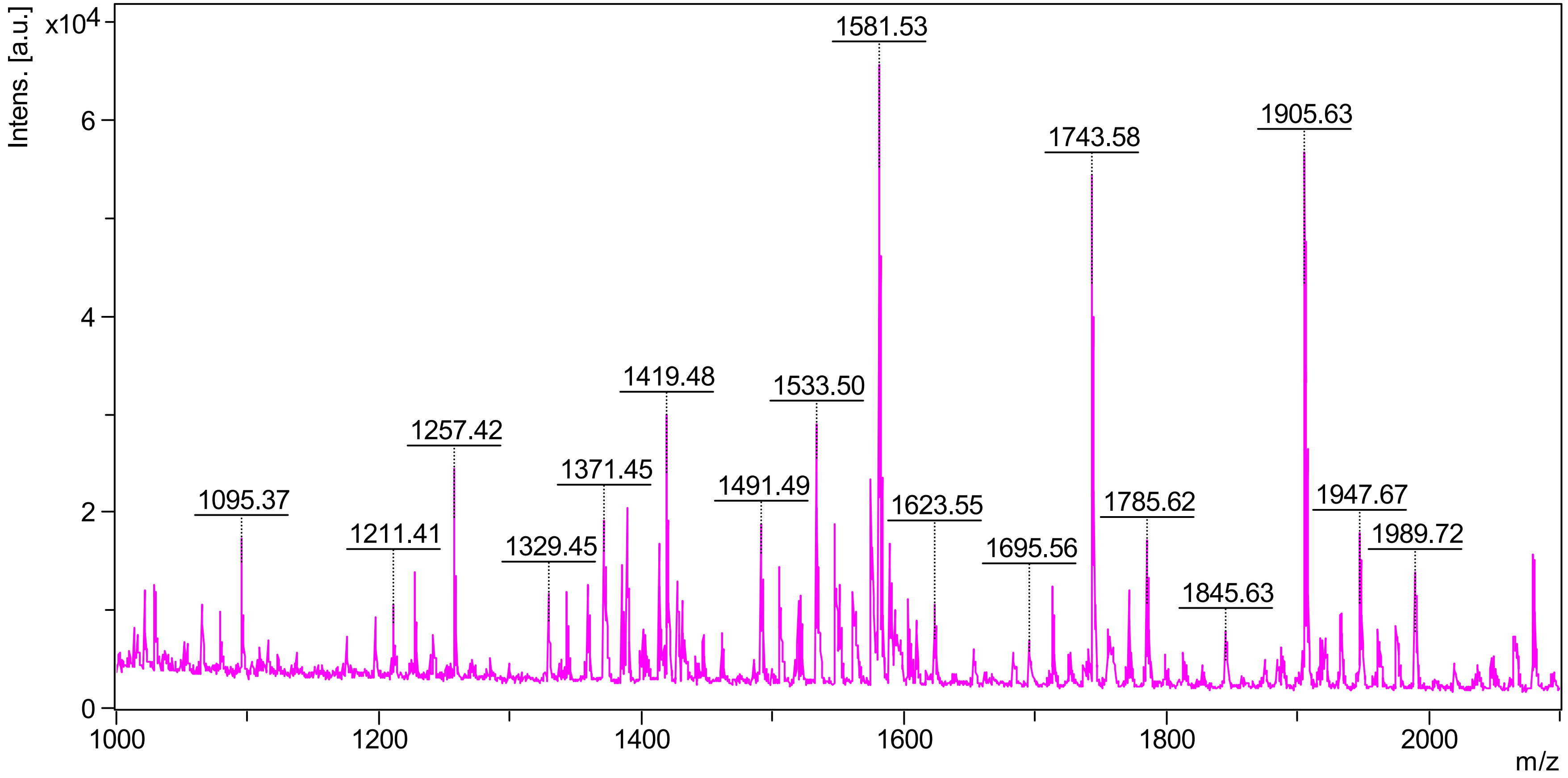

Now Foods LIFT

Asp

Asp C-26

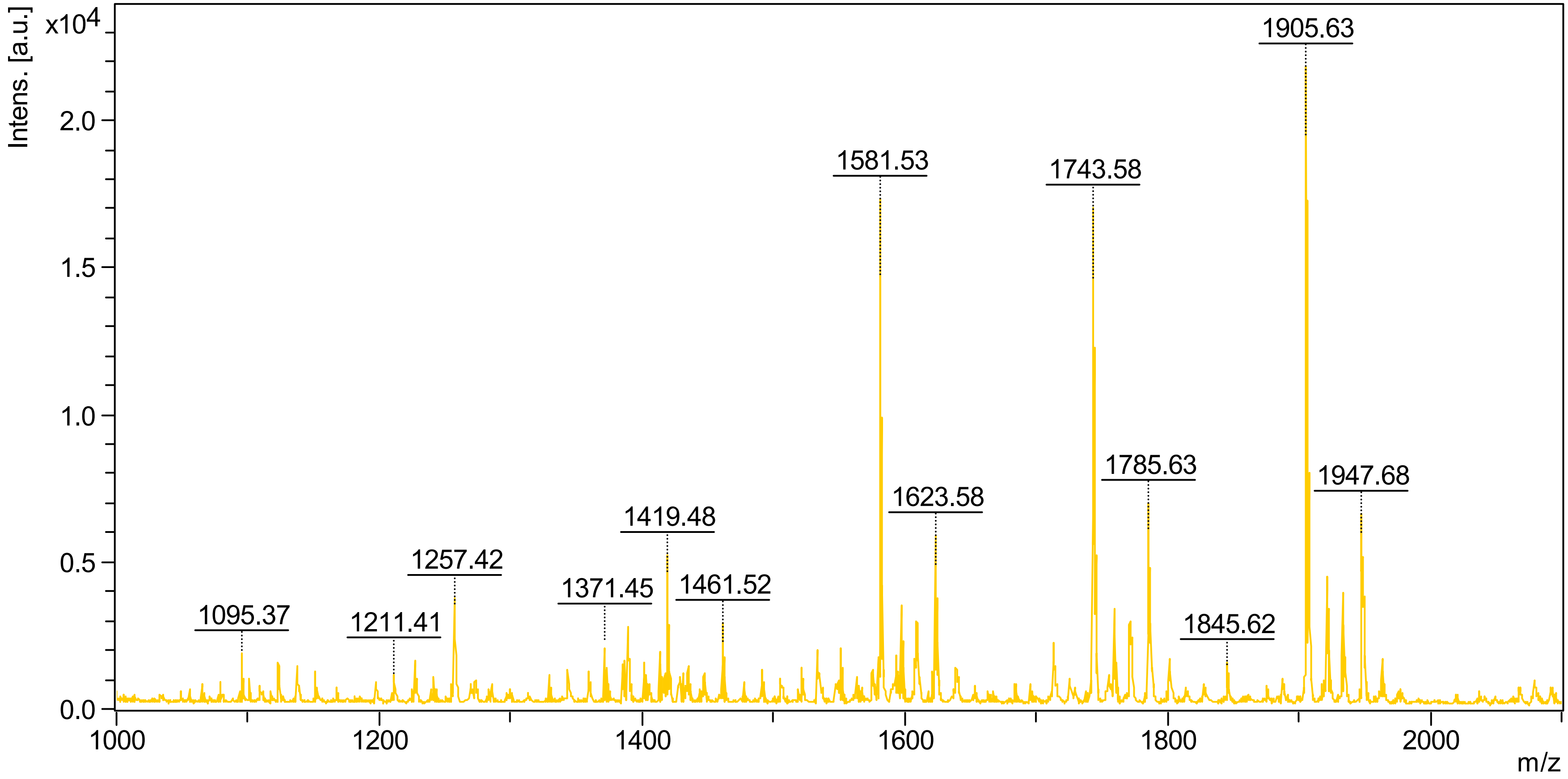

Bio Pure Apr19

Asp

Asp C-57

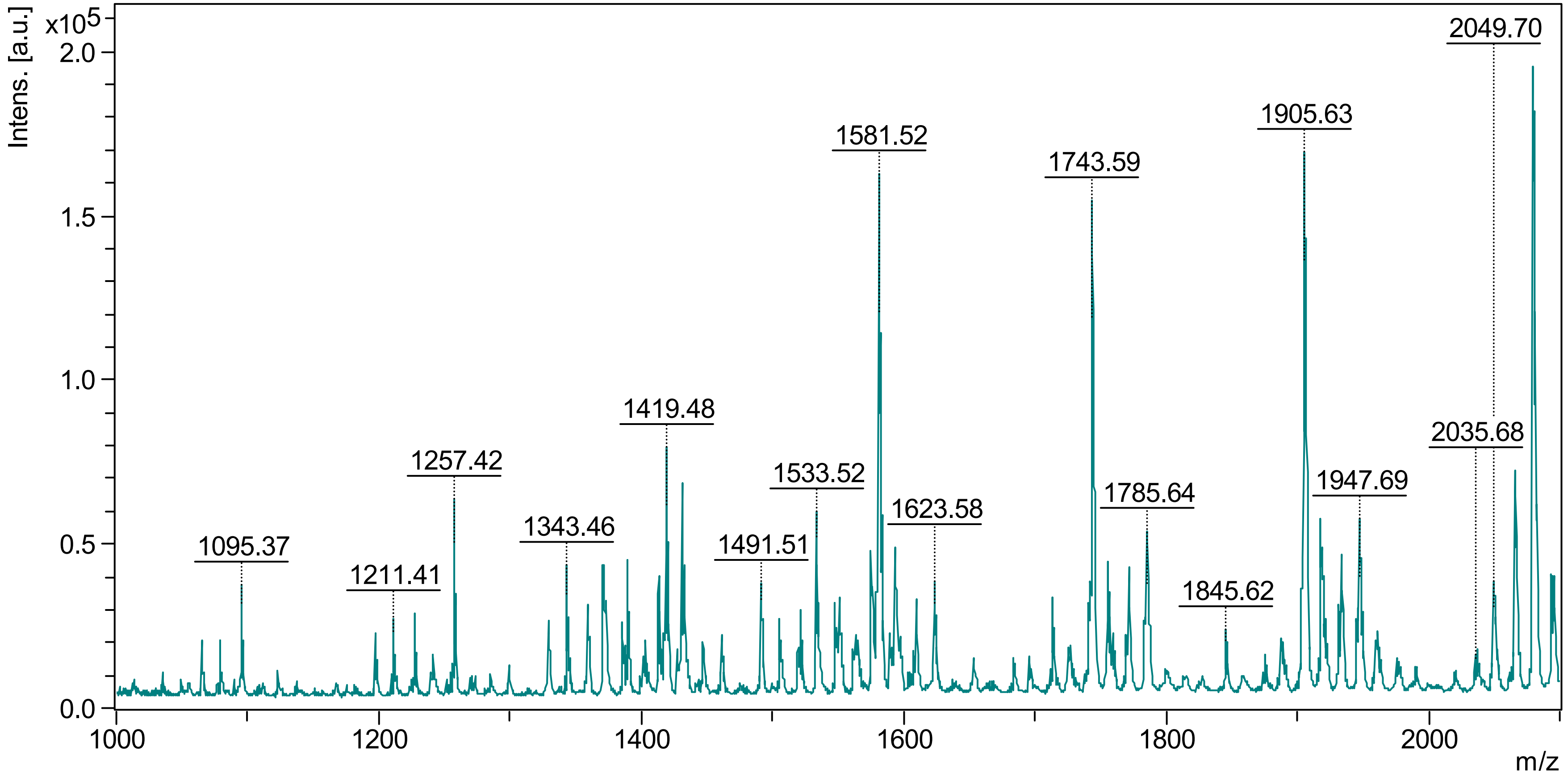

Alge 57 von 37 Bio-Hau

Asp

Asp C-59

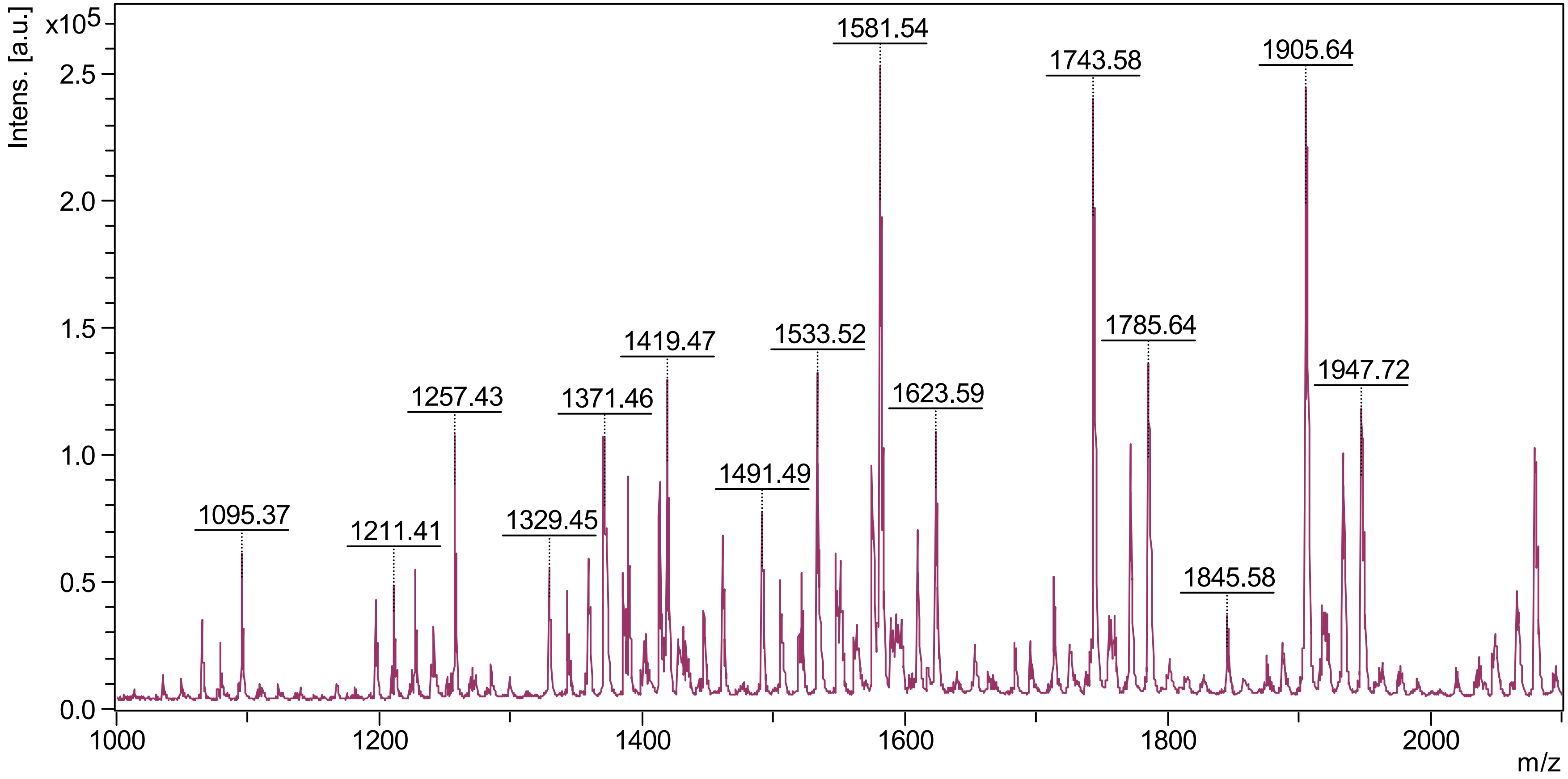

Alge 59 von 39 purewell

Asp

Asp C-71

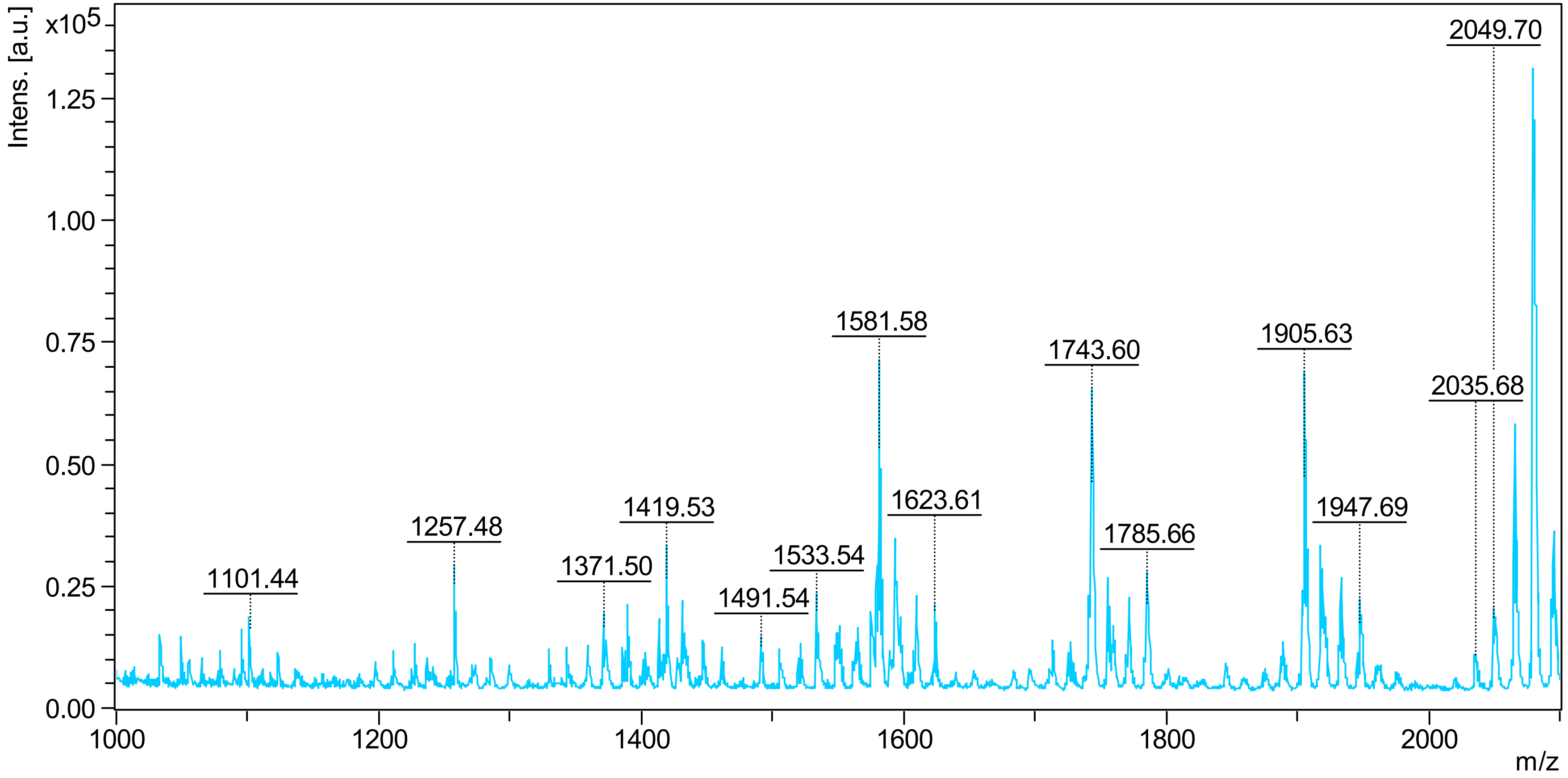

Alge 71 von 51 Biokräfte

Asp

Asp C-73

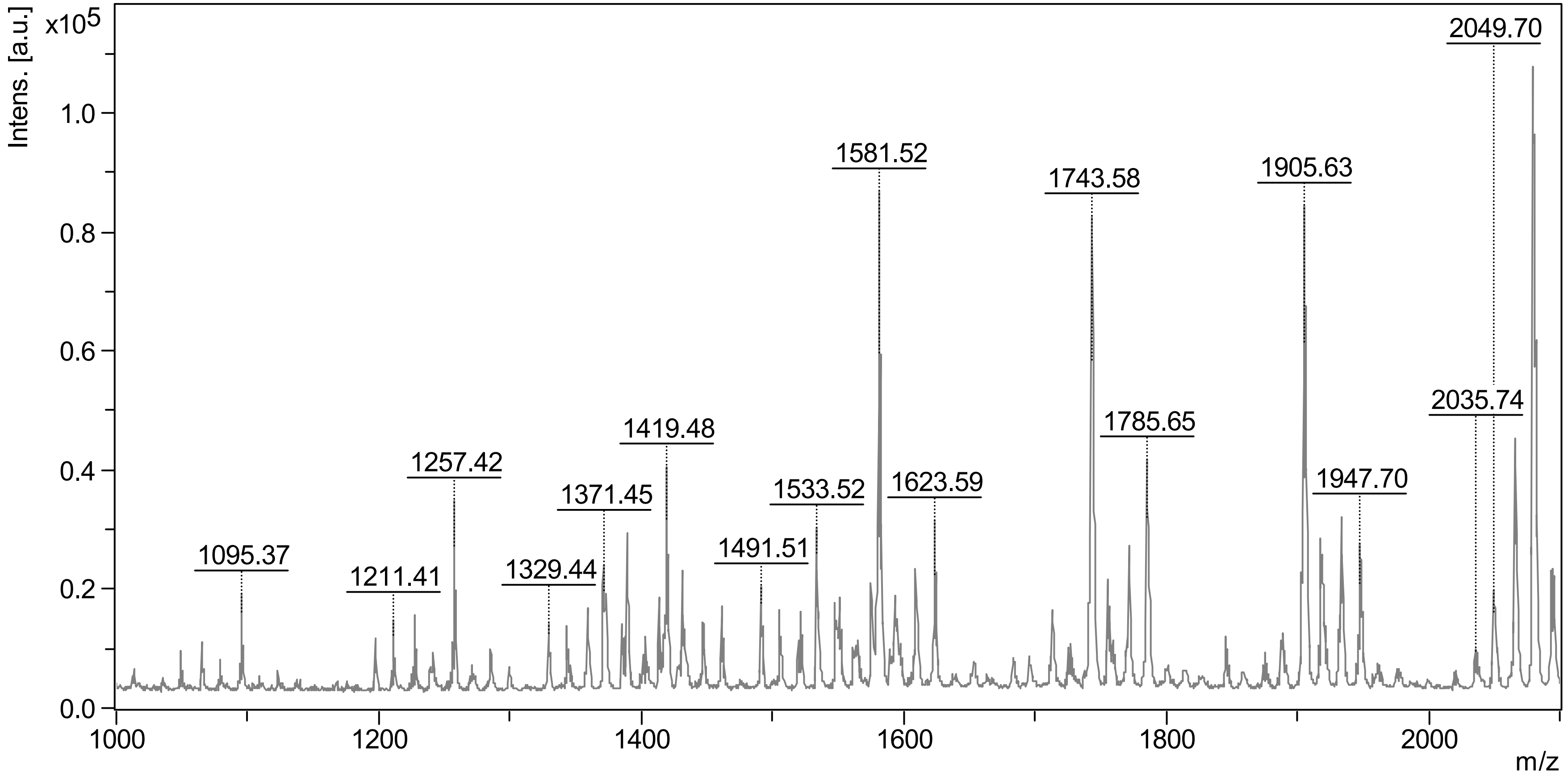

Alge 73

Asp

Asp C-74

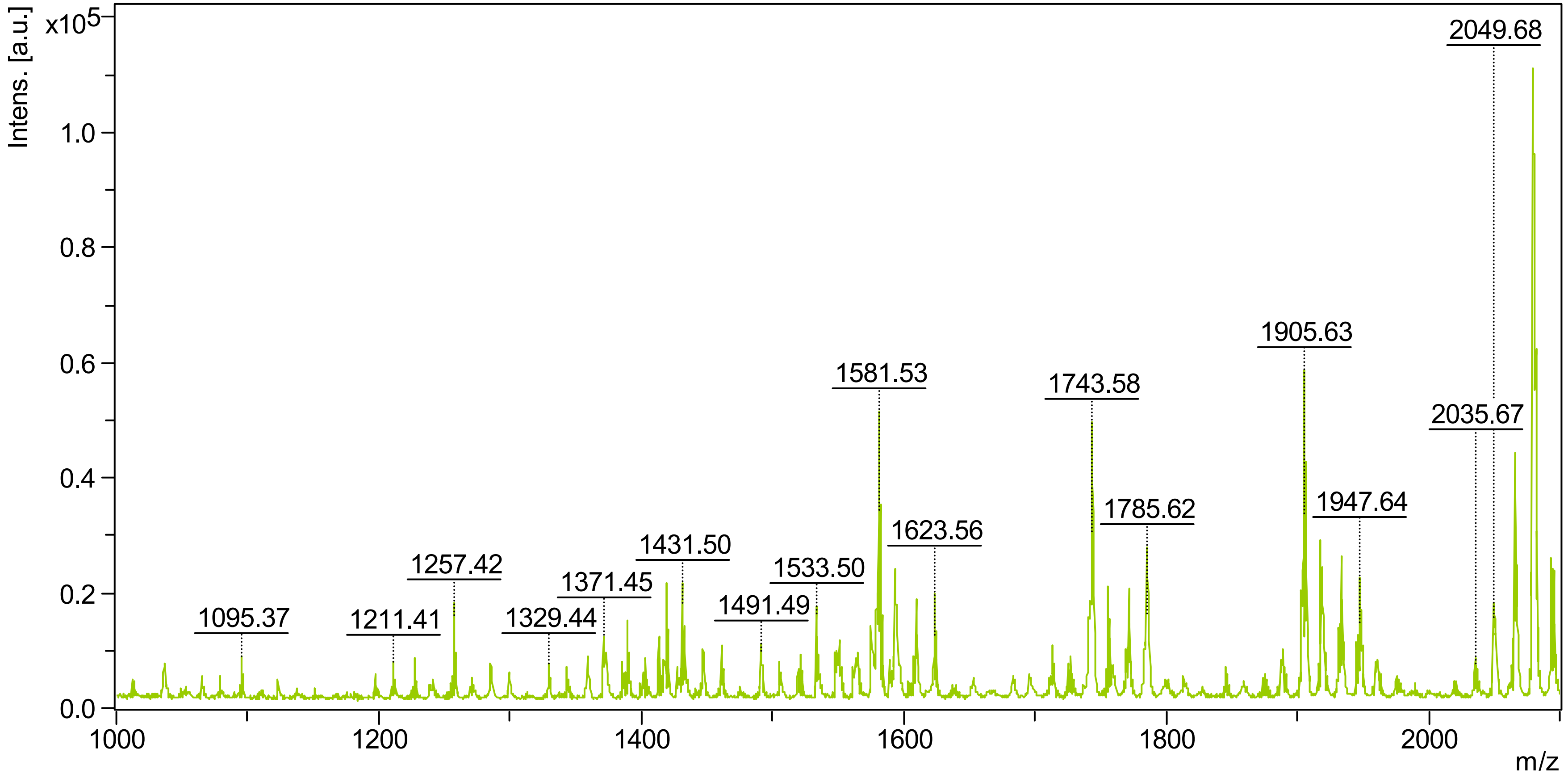

Alge 74

Asp

Asp C-77

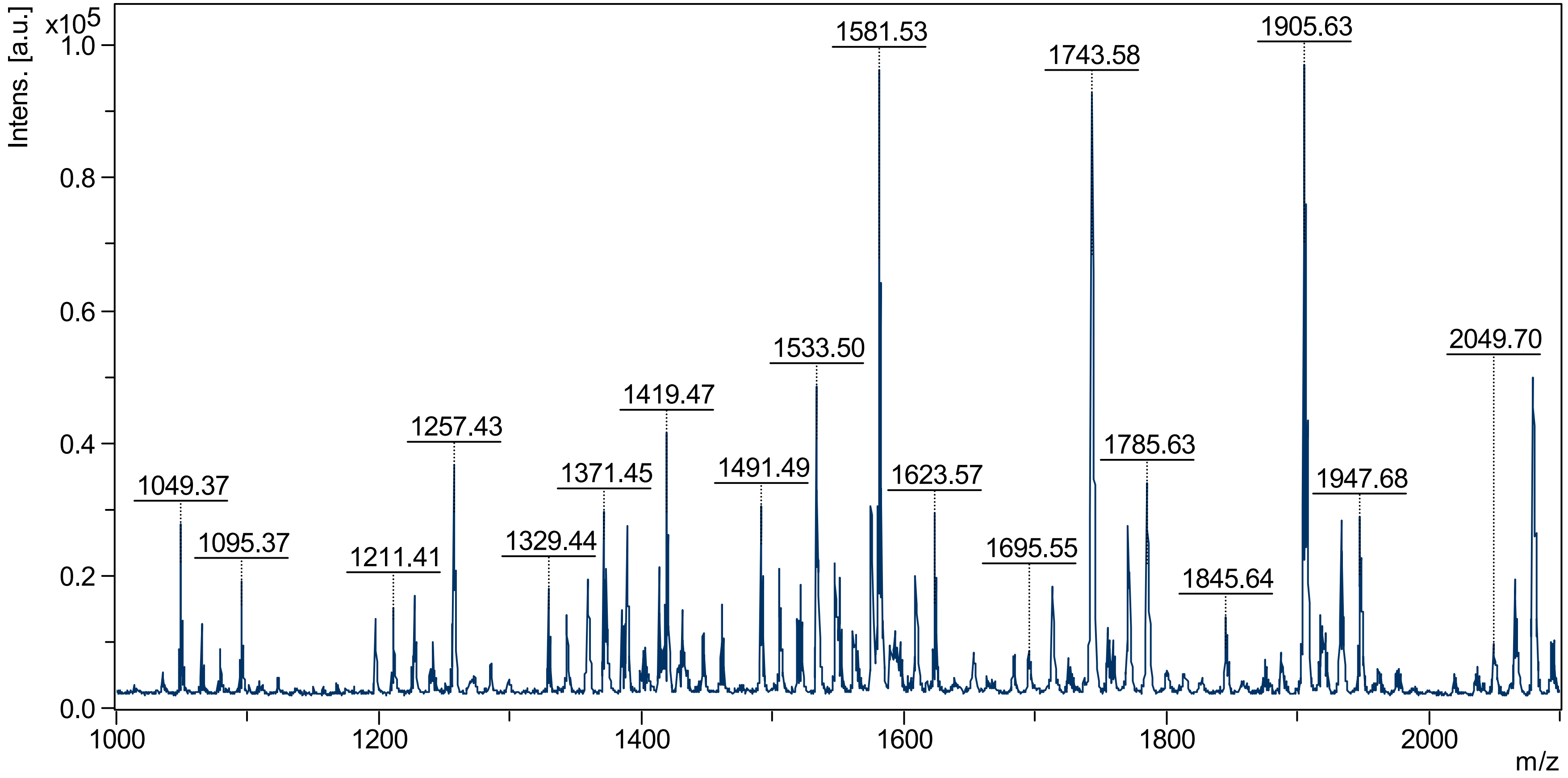

Alge 77

Asp

Asp C-81

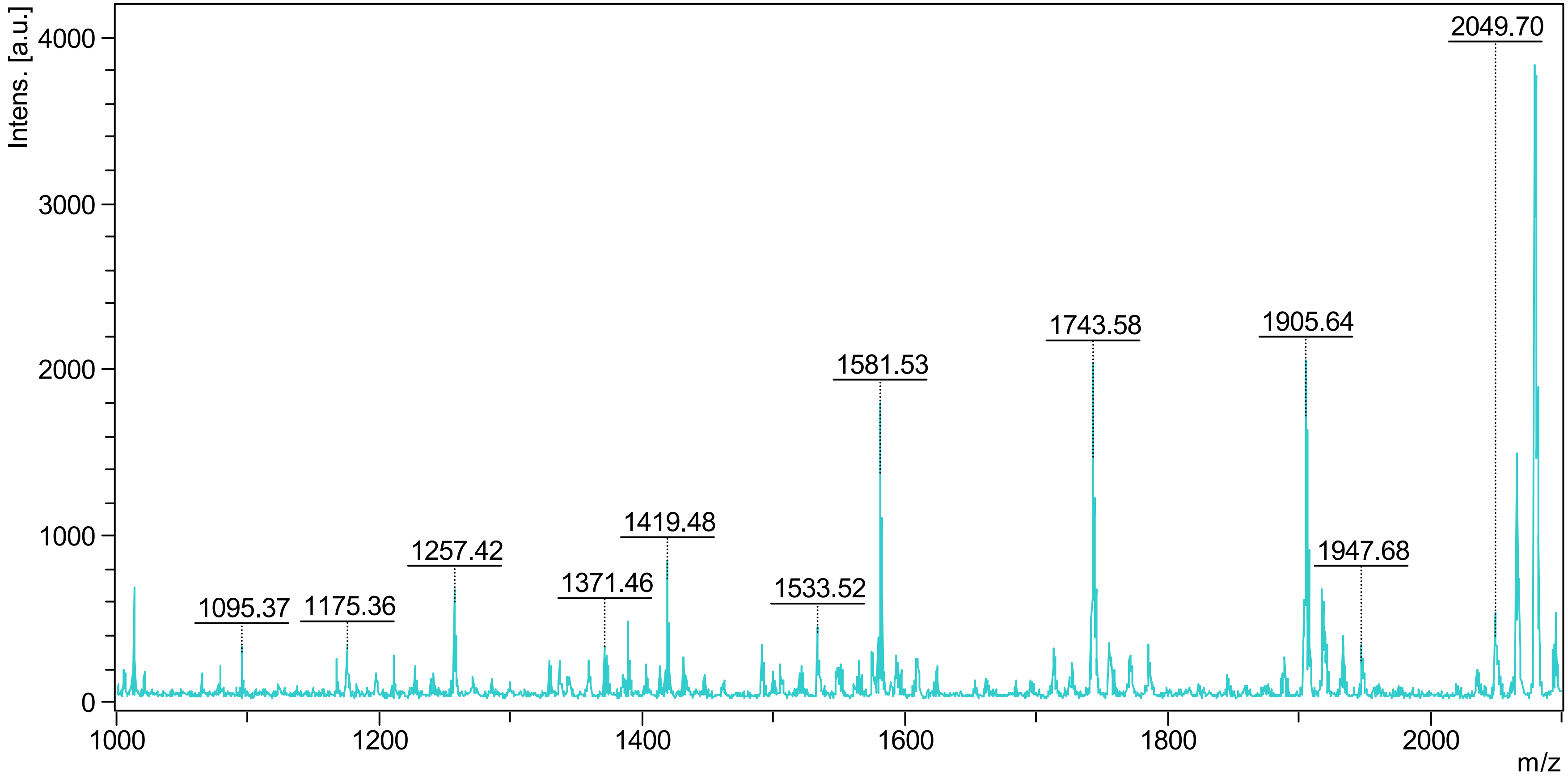

Alge 81 Vitawell

Ama

Ama C-38

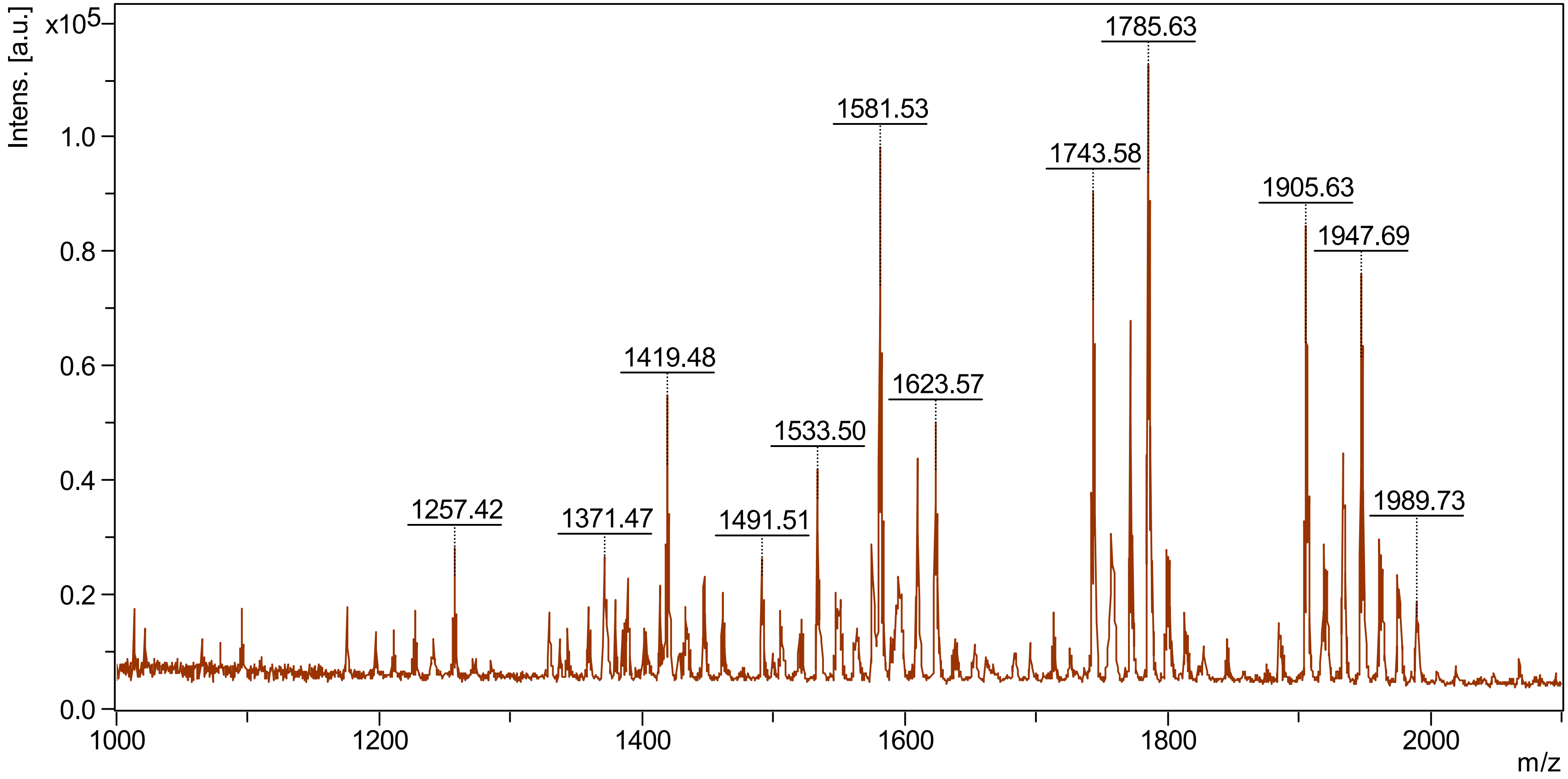

GSE

Ama

Ama C-41

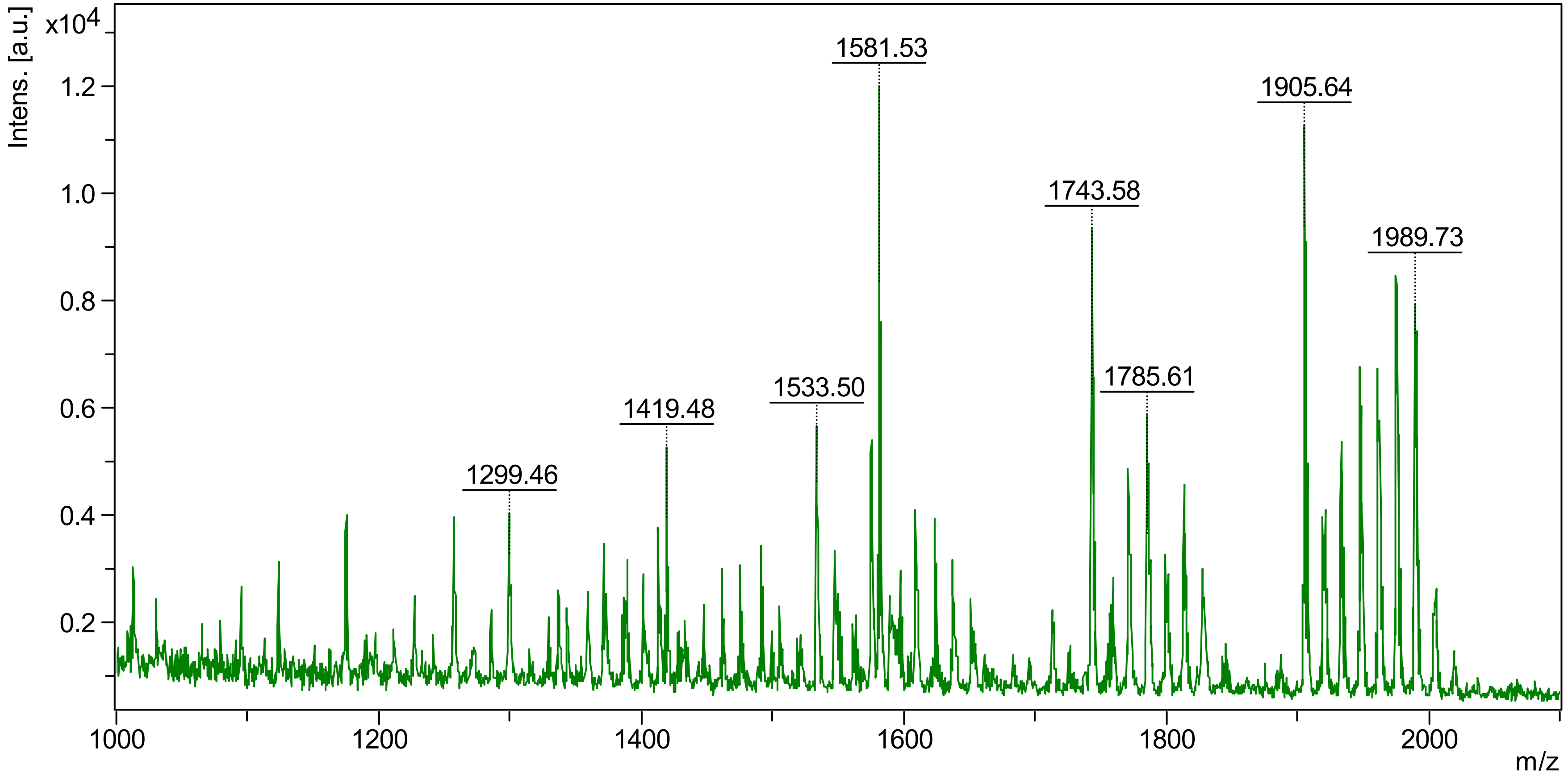

20180109 Weihnachtsmarkt D1

Pit

Pit C-15

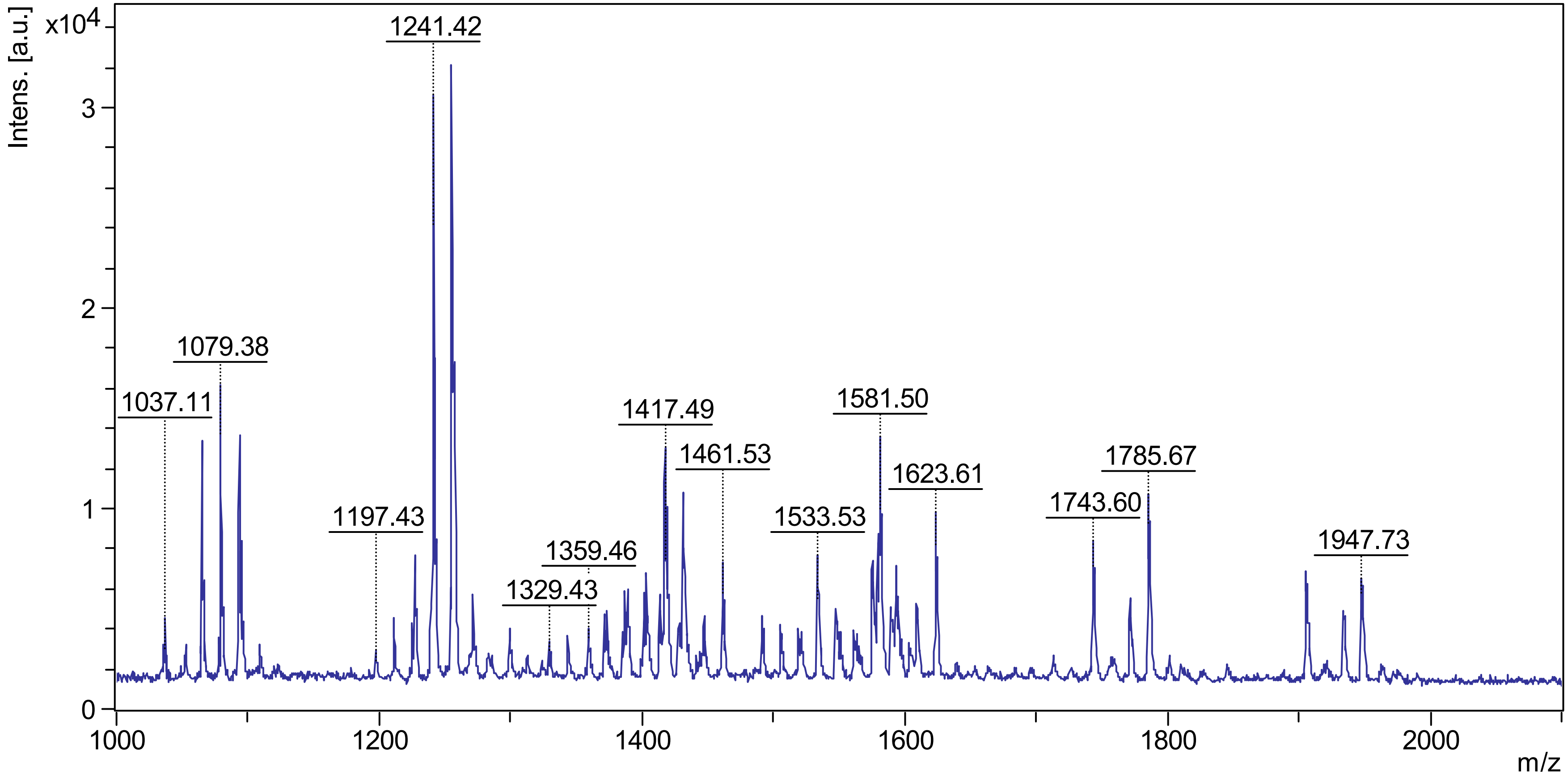

20150617 PitPit (Lisi_5)

Pit

Pit C-38

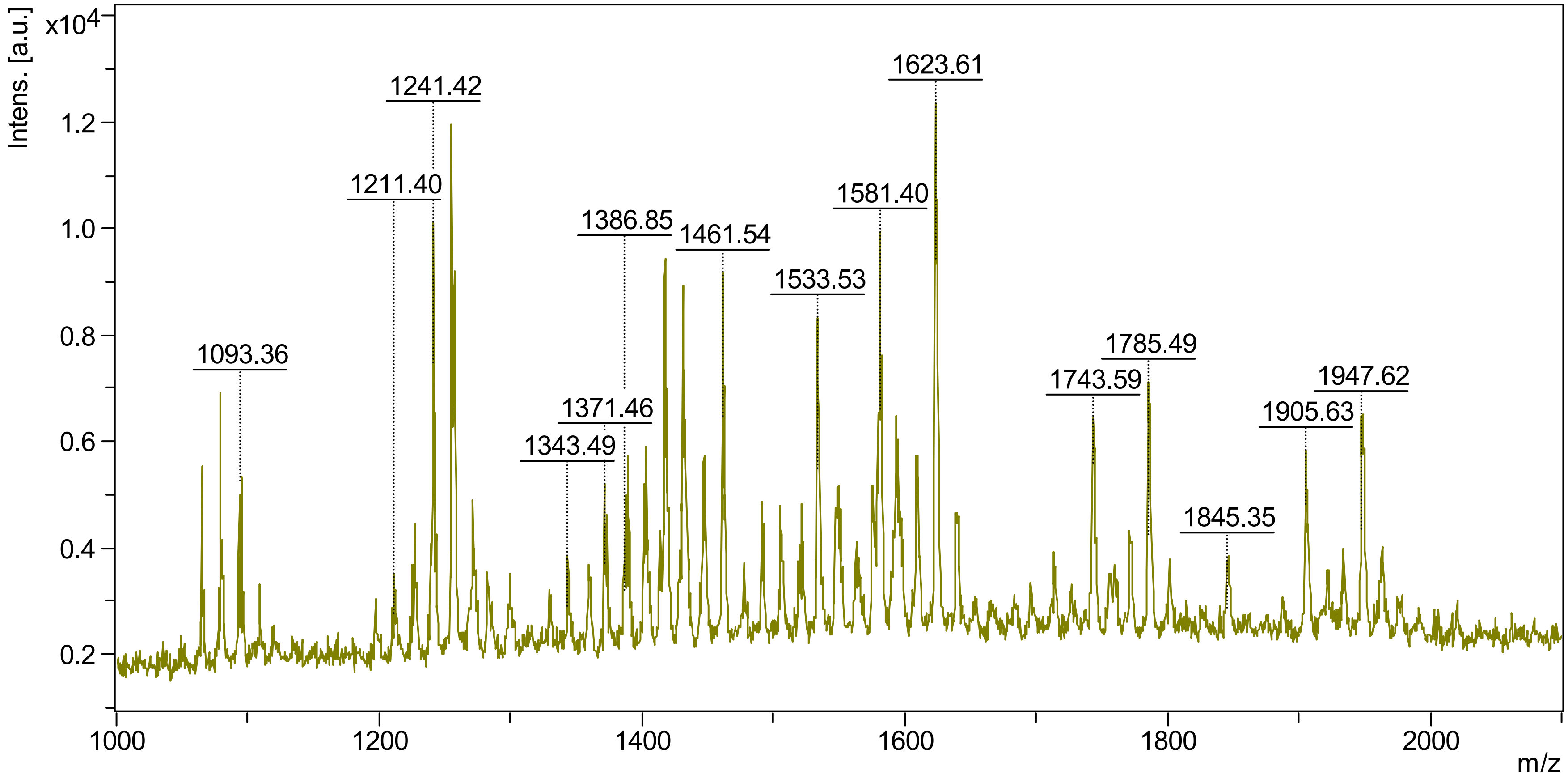

Superfood Tausendkraut
